# Between duty and constraint: a qualitative systematic review of healthcare providers' ethical challenges and moral stressors in caring for undocumented migrants

**DOI:** 10.1080/17482631.2026.2701615

**Published:** 2026-07-09

**Authors:** Fayez Abdulrazeq, Chris Gastmans, Nikola Biller-Andorno, Julian März

**Affiliations:** a Institute of Biomedical Ethics and History of Medicine (IBME), Faculty of Medicine, University of Zurich, Zurich, Switzerland; b Centre for Biomedical Ethics and Law (CBMER), Faculty of Medicine, Katholieke Universiteit Leuven (KU Leuven), Leuven, Belgium

**Keywords:** Systematic review, qualitative analysis, healthcare providers, undocumented migrants, ethical challenges, moral stress

## Abstract

**Purpose:**

Healthcare providers play a critical role in delivering care to undocumented migrants, who face systemic barriers to healthcare access. While research has documented undocumented migrants’ legal and policy barriers, less is known about the ethical challenges and moral stressors experienced by providers. This systematic review synthesizes qualitative studies on providers' experiences when delivering care to undocumented migrants.

**Methods:**

The review followed PRISMA guidelines. PubMed, Embase, CINAHL, and the Cochrane Library were searched for relevant qualitative studies. Studies were screened by title, abstract, and full text using predefined inclusion and exclusion criteria. Quality assessment was conducted using the CASP checklist. Data were synthesized using the Qualitative Analysis Guide of Leuven (QUAGOL), integrating Graneheim and Lundman’s approach to qualitative content analysis.

**Results:**

The systematic search identified 37 qualitative studies. Analysis revealed 58 themes and subthemes, organized under five key concepts: experiences, perceptions, attitudes, practices and coping mechanisms, and ethical challenges. Providers reported moral distress, emotional exhaustion, and professional dilemmas arising from legal constraints, resource limitations, and conflicting obligations. Ethical tensions centered on beneficence vs. non-maleficence, professional duty vs. legal compliance, and the moral dilemma of deservingness. Across these findings, the synthesis identifies ethical burden-shifting as a central analytical contribution: restrictive systems transfer the moral and practical consequences of exclusionary arrangements onto providers and undocumented migrants at the point of care.

**Conclusion:**

This review shows that providers’ ethical challenges are not only individual clinical dilemmas but structurally generated moral stressors. Addressing these challenges requires structural interventions, including policy reforms that reconcile professional ethics with legal constraints and institutional support to mitigate moral distress.

## Introduction

The provision of healthcare to undocumented migrants raises complex ethical questions for healthcare providers, particularly when efforts to deliver care and advocate for patients' needs occur within restrictive legal and sociopolitical environments often marked by stigma and discrimination toward undocumented migrants (Abdulrazeq et al., 2024; Abubakar et al., 2018; Suphanchaimat et al., 2015). This complexity is closely linked to the heightened vulnerability of undocumented migrants themselves. Many are compelled to leave their countries of origin due to severe hardships, undocumented migrants often face exclusion from healthcare services beyond emergency care, including maternal and child health services, chronic disease management, mental health services, and preventive care, primarily as consequence of their irregular legal status^1^ (Kisa & Kisa, 2024; Moezzi et al., 2024; Ruiz-Casares et al., 2013; Woodward et al., 2014). This exclusion reflects a political climate in which access to healthcare is framed as a citizenship-based entitlement rather than a universal human right (Dwyer, 2004). Within this context, policies shaped by anti-immigration rhetoric and political agendas often portray undocumented immigrants as responsible for broader economic and social pressures, thereby reinforcing processes of marginalisation and social exclusion (Martinez et al., 2015).

Against this backdrop, a central ethical conflict emerges between healthcare providers' professional duty to deliver care based on medical need and policy frameworks that restrict access to healthcare for undocumented migrants (Brenner et al., 2021; Suphanchaimat et al., 2015). This conflict is further intensified by the absence of coherent and consistent legal frameworks governing healthcare delivery for this population (Moezzi et al., 2024). Together, these conditions illustrate the dynamic interplay between healthcare policy, immigration law, and societal attitudes, producing persistent barriers to equitable care for undocumented migrants, including restricted access to essential medicines, administrative obstacles that disrupt continuity of care across the migration trajectory, and health inequalities that are further exacerbated during public health crises and pandemics (Aljadeeah et al., 2024; El Arab et al., 2023). While prior scholarship has documented barriers to access and the legal–policy conditions shaping migrants’ entitlements (Abubakar et al., 2018; Juárez et al., 2019; Kisa & Kisa, 2024; Martinez et al., 2015; Suphanchaimat et al., 2015), the ethical challenges and moral stressors experienced by healthcare providers in this context remain dispersed across qualitative studies and have not been systematically synthesised in a way that integrates their interrelationships, practice-level consequences, and implications for policy and institutional support.

At their core, healthcare providers are guided by the ethical principles of beneficence, non-maleficence, and justice, which obligate them to provide care to all patients, irrespective of immigration status (Brenner et al., 2021). However, these principles frequently clash with systemic barriers, compelling providers to adopt “workarounds” to secure necessary care for their patients within restrictive institutional and legal environments. While these strategies are well-intentioned, they may raise ethical concerns and prove unsustainable, as they rely on individual discretion rather than formalised entitlements, further intensifying the challenges faced by providers over time (Berlinger, 2019).

Moreover, negative stereotypes and biases about undocumented migrants can unconsciously shape healthcare providers' decisions and behaviours, potentially leading to prejudice and discrimination. For instance, providers may perceive undocumented migrants as misusing healthcare resources or disproportionately burdening the system. Such perceptions can result in explicit or implicit rationing of care and less compassionate treatment (Woofter & Sudhinaraset, 2022). Additionally, healthcare providers often grapple with tensions between their professional obligation to uphold patient confidentiality and external pressures to report undocumented patients to immigration authorities. This conflict undermines trust, deters migrants from seeking care, and adversely impacts health outcomes (Kim et al., 2019; Papageorgiou et al., 2020). In navigating these circumstances, healthcare providers may feel complicit in enforcing policies they perceive as unjust, further intensifying their ethical and moral stress (Berlinger, 2019).

A systematic understanding of the ethical challenges and moral stressors that healthcare providers encounter when caring for undocumented migrants is essential. Such understanding is critical for advancing health equity and advocating for undocumented migrants’ health entitlements, informing policy and institutional responses, and supporting provider well-being (Abdulrazeq et al., 2024; Suphanchaimat et al., 2015). Although existing literature has shed light on some of these challenges, notable gaps persist. For example, comprehensive ethical guidelines specifically designed to assist providers in navigating the complexities of caring for undocumented migrants are largely absent (Jawed et al., 2021; Onarheim et al., 2021). In the absence of such guidelines, providers are often left to independently address complex legal and ethical dilemmas, leading to inconsistent care practices and heightened moral distress (Jawed et al., 2021). Furthermore, while the prevalence of moral distress among healthcare providers has been acknowledged in the literature, further research is needed to examine its specific impacts, underlying causes, and the coping mechanisms employed by providers.

To contribute to addressing these gaps, this review consolidates qualitative evidence from diverse studies examining healthcare providers' experiences across a range of healthcare settings and policy contexts. Qualitative research is particularly valuable in this context, as it provides nuanced insights into providers' perspectives and the ethical dilemmas they face within structurally constrained environments (Abdulrazeq et al., 2024). Unlike quantitative designs, which can estimate the prevalence or correlates of moral distress and burnout, qualitative studies can illuminate how providers interpret conflicting obligations, make sense of perceived injustice, and negotiate constraints in everyday clinical encounters. Qualitative evidence is also well suited to capturing relational dynamics—such as trust, tensions surrounding confidentiality and reporting, experiences of stigma, and the ethical tensions that shape provider–patient interactions—that are central to ethical analysis but are not readily observable through administrative data or survey measures. The central research question guiding this systematic review is: What ethical challenges and moral stressors arise for healthcare providers when delivering care to undocumented migrants?

By synthesising findings from various healthcare contexts, including governmental and humanitarian settings, this review contributes to the existing literature by offering a systematically derived overview of the ethical challenges surrounding healthcare provision for undocumented migrants. We argue that these challenges are not merely isolated dilemmas located within individual clinical encounters. Rather, they are structurally generated, institutionally and socially shaped moral stressors that emerge when restrictive entitlement regimes, institutional constraints, resource limitations, and social judgements about migrant deservingness are translated into everyday healthcare practice. In this sense, the synthesis identifies a pattern of ethical burden-shifting, whereby restrictive systems displace moral responsibility onto healthcare providers, requiring them to absorb and negotiate the ethical consequences of exclusionary policies in daily practice. Through an integrative conceptual scheme, we show how healthcare providers’ experiences, perceptions, attitudes, practices and coping mechanisms, and ethical challenges interact to shape both provider well-being and the care made available to undocumented migrants. This synthesis therefore clarifies pathways through which structural constraints become moral stress.

To translate these findings into actionable outputs, we also propose stakeholder-specific recommendations and future research trajectories that build directly on the synthesis and indicate priority areas for policy, institutional support, and qualitative enquiry. By shedding light on the experiences of healthcare providers, this review contributes to raising awareness, promoting ethical reflection, and encouraging the development of ethical guidelines and institutional support mechanisms to reduce the burden placed on individual providers while strengthening ethically responsive care for undocumented migrants.

## Healthcare policy contexts for undocumented migrants across the included countries

The studies included in this review encompass diverse healthcare contexts across thirteen countries, reflecting a wide range of migrant health policy environments. These span North America (United States); Western Europe (Spain, Belgium, France, Germany); Northern Europe (Denmark, England, the Netherlands, Norway, Sweden); Central America (Costa Rica); Western Asia (Israel); and Southeast Asia (Thailand). Across these countries, healthcare systems and the scope of entitlements for undocumented migrants differ markedly—ranging from highly restrictive frameworks, such as in Denmark and the United States, where access is largely confined to emergency services, to more inclusive approaches, as observed in Spain and Thailand, which extend to primary care and beyond.

However, even in countries that grant broader healthcare entitlements to undocumented migrants, challenges often emerge in the practical implementation of these rights. While legal frameworks may, in principle, ensure comprehensive access, the translation of these rights into effective practice is frequently impeded by a combination of structural, administrative, and individual-level barriers—including those associated with fear of deportation, social marginalisation, and precarious living conditions. For example, in Costa Rica and the Netherlands, despite policies supporting broad access, practical obstacles continue to constrain the full realisation of these entitlements.

Another critical issue within this landscape is the tendency to treat undocumented migrants as a homogeneous population—an approach that often obscures the diverse vulnerabilities and specific needs that exist within this group. Some countries have adopted more nuanced policy frameworks, extending healthcare access to particular subgroups such as children and pregnant women, while others maintain a uniform approach that fails to recognise these distinctions. In countries such as Germany and Israel, where such differentiation is absent, the lack of tailored policies can exacerbate the challenges faced by especially vulnerable subgroups. Failing to account for the heterogeneity among undocumented migrants ultimately limits the effectiveness of healthcare entitlements and perpetuates inequities within broader health policy frameworks.

To orient readers to the scope of healthcare entitlements afforded to undocumented migrants, the supplementary material provides a concise, country-by-country overview of the healthcare policy contexts within which healthcare providers operate when delivering care to this population. This overview is intended solely as descriptive background to clarify the policy settings relevant to the included studies, without modifying the aims of the systematic review or the methods of data analysis. A formal comparative policy analysis lies beyond the scope of this qualitative evidence synthesis and was not used to inform data coding or theme development. Readers are encouraged to consult this supplementary section for contextual reference rather than to interpret it as an explanatory framework for the themes.^2^

## Methods

We conducted this systematic review in accordance with the Preferred Reporting Items for Systematic Reviews and Meta-Analyses (PRISMA) guidelines (Moher et al., 2009); a detailed checklist outlining how each item was addressed is provided in the supplementary material.

### Search strategy

We conducted two rounds of systematic searches for relevant qualitative studies that met our inclusion criteria: the first in March 2023 and the second in May 2024, using the same search strategy across the same databases. This approach ensured our systematic review remained up-to-date. We searched four electronic databases—PubMed/Medline, Embase, CINAHL, and the Cochrane Library—without any limitations on the publication date. The searches were restricted to studies published in English.

Our broad search strategy combined three major search blocks using the Boolean operator “AND”: (1) undocumented migrants, (2) healthcare providers or healthcare delivery, and (3) ethical challenges. Within each search block, we employed both Medical Subject Headings (MeSH) and keyword-based (free-text) search strings, linked by the Boolean operator “OR”. This approach provided optimal coverage by ensuring the capture of articles indexed with MeSH terms, as well as those using relevant keywords but not yet fully indexed or using different terminology. Given that not all databases utilise standardised MeSH terms, we first identified the relevant MeSH terms in PubMed/Medline and then adapted these terms for the other three databases.

Prior to the systematic review, we performed an exploratory scoping exercise to test the relevance of our search terms and refine our search strategy to accurately capture the three search blocks. We trialled various keyword combinations to achieve an appropriate balance between sensitivity and specificity. To enhance the second search block, we expanded the search string to include additional terms such as physician-patient relations, health services accessibility, and other related terms. This resulted in a manageable set of less than 3,000 records for manual screening.

To identify additional citations of interest, we performed a snowball search by reviewing the reference lists of included studies. For replicability, an example of the search strategy used for the PubMed/Medline database is provided in Table I.

**Table I.** Search strings used for the PubMed/Medline database, stratified across three search blocks.

Search block	Search string
First: Undocumented migrants	exp undocumented immigrants/or undocumented.ab, kw, ti. or unauthorised immigrant$.ab, kw, ti. or unauthorised migrant$.ab, kw, ti. or unauthorised worker$.ab, kw, ti. or illegal immigrant$.ab, kw, ti. or illegal migrant$.ab, kw, ti. or illegal worker$.ab, kw, ti. or irregular migrant$.ab, kw, ti. or irregular immigrant$.ab, kw, ti. or irregular worker$.ab, kw, ti. or rejected asylum seeker$.ab, kw, ti.
Second: Healthcare delivery by healthcare providers	exp “delivery of health care”/or exp health services accessibility/or exp healthcare disparities/or exp right to health/or exp patient advocacy/or exp physician-patient relations/or exp health personnel/or exp physicians/or exp allied health personnel/or personnel.ab, kw, ti. or physician$.ab, kw, ti. or doctor$.ab, kw, ti. or nurse$.ab, kw, ti. or health provider$.ab, kw, ti. or health worker$.ab, kw, ti. or professionals.ab, kw, ti. or healthcare.ab, kw, ti. or care.ab, kw, ti. or therapy.ab, kw, ti. or treatment.ab, kw, ti.
Third: Ethical challenges	exp ethics/or exp bioethics/or exp moral obligations/or exp ethics, clinical/or exp ethics, institutional/or exp jurisprudence/or exp ethics committees, clinical/or exp “codes of ethics”/or exp ethics consultation/or exp principle-based ethics/or exp ethics committees/or exp ethics, professional/or exp ethics, nursing/or exp ethics, medical/or exp ethical theory/or ethic$.ab, kw, ti. or bioethic$.ab, kw, ti. or moral$.ab, kw, ti. or “professional obligation$”.ab, kw, ti. or responsabilit$.ab, kw, ti. or issue$.ab, kw, ti. or challenge$.ab, kw, ti. or dilemma$.ab, kw, ti.

### Inclusion and exclusion criteria

We established inclusion and exclusion criteria to capture qualitative studies most relevant to our research question: What ethical challenges and moral stressors arise for healthcare providers when delivering care to undocumented migrants? The full criteria are outlined in Table II.

**Table II.** The inclusion and exclusion criteria used in our systematic review.

Inclusion	Empirical qualitative studies with no restrictions on data collection techniques. Studies focusing on healthcare delivery for undocumented migrants that explore challenges from the perspective of healthcare providers. Studies published in English, with no geographic limitations on where the study was conducted
Exclusion	Quantitative studies, theoretical papers, reviews, commentaries, opinion articles, editorial notes, conference abstracts without full texts, case reports, guidelines, policy papers, content analysis papers, books, book chapters, and dissertations. Quantitative studies, theoretical papers, reviews, commentaries, opinion articles, editorial notes, conference abstracts without full texts, case reports, guidelines, policy papers, content analysis papers, books, book chapters, and dissertations.2. Studies focusing on healthcare delivery for other migrant or vulnerable groups, rather than undocumented migrants.

Our inclusion criteria focused on qualitative studies due to their ability to explore the complex and multifaceted nature of healthcare delivery for undocumented migrants. These methods provide deep insights into healthcare providers' lived experiences, allowing for a rich exploration of the personal, professional, and situational contexts that are essential for addressing our research question. By focusing on qualitative research, we aimed to capture the ethical challenges and moral stressors healthcare providers face when delivering care to undocumented migrants.

We adopted a broad definition of health that encompasses physical, mental, and social well-being, aligned with the World Health Organisation’s perspective on the interconnectedness of these dimensions. Based on this understanding, we included qualitative studies examining healthcare delivery in any of these areas from the perspective of healthcare providers. Furthermore, we did not restrict studies by the type of healthcare setting, recognising that different environments—whether humanitarian, governmental, or private—may generate distinct ethical challenges.

Our inclusion criteria were specifically limited to studies focused on healthcare delivery for undocumented migrants. This decision reflects our interest in understanding the unique ethical challenges that healthcare providers face when working with this vulnerable group, whose legal status creates specific barriers such as fear of deportation, lack of legal protection, and social marginalisation. By narrowing our focus to undocumented migrants—defined as individuals who have crossed international borders without legal authorisation to enter or remain—we aimed to generate evidence directly relevant to healthcare providers working with this specific population, rather than generalising findings across all migrant groups. This definition includes subgroups such as rejected asylum seekers, undocumented workers, and individuals who have overstayed their visas.

Using these inclusion and exclusion criteria, we conducted a systematic screening of candidate articles based on title, abstract, and full text. The first author performed the initial screening, and exclusions were verified by the last author. We then engaged in collaborative discussions to finalise article inclusion decisions, and any discrepancies were resolved through discussion with the other two co-authors until consensus was reached.

### Quality appraisal

We systematically assessed each study to evaluate its trustworthiness, value, and relevance using the Critical Appraisal Skills Programme (CASP) quality assessment tool for qualitative research . Since critical appraisal involves subjective judgement, we began by carefully reading each paper to understand its aims, methodology, results, conclusions, and to identify any limitations or biases. Next, we applied the CASP checklist, ensuring all ten assessment criteria were addressed and keeping detailed records of our evaluations. A summary of individual assessments for each study is provided in the supplementary material.

Each criterion was scored based on whether it was met: “Yes” received a score of 2, “Unclear” a score of 1, and “No” a score of 0. Consequently, the total score for each study ranged from 0 (lowest possible score) to 20 (highest possible score). Studies were then categorised according to their total scores as follows: poor quality (0–4), low quality (5–8), moderate quality (9–12), good quality (13–16), and high quality (17–20). Notably, no studies were excluded based on quality.

### Data extraction and synthesis

We performed data extraction and synthesis using the Qualitative Analysis Guide of Leuven (QUAGOL) approach (Dierckx de Casterlé et al., 2012, 2021). This approach was selected for its ability to combine case-oriented and cross-case analysis of qualitative data, incorporate forward-backward dynamics and the constant comparative method from grounded theory, and emphasise teamwork.

**Figure 1.**
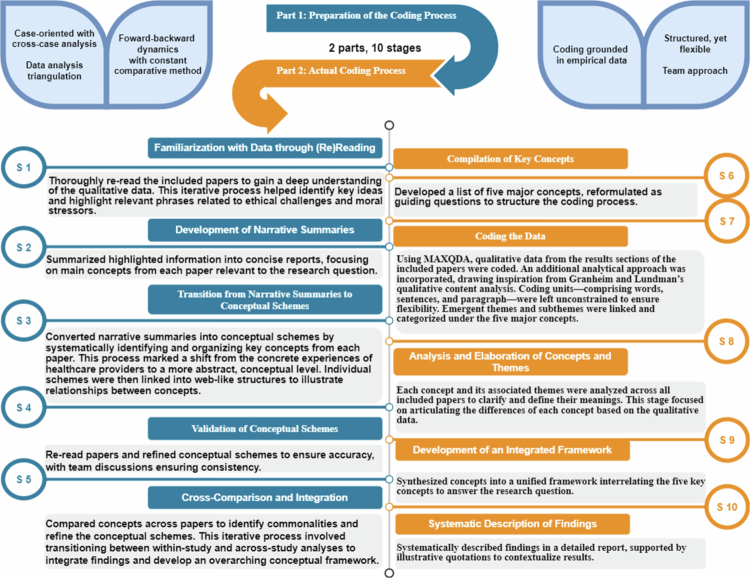
Data analysis and synthesis process.

The process consisted of two main parts: preparation for coding and the actual coding, with each part comprising five stages, making a total of ten stages. At stage seven of the QUAGOL approach, we incorporated an additional analytical strategy to guide the coding process, drawing inspiration from Graneheim and Lundman’s approach to qualitative content analysis (Graneheim & Lundman, 2004). For a detailed overview of the stages and our data analysis and synthesis strategy, please refer to Figure 1.

## Results

### Study characteristics

**Figure 2.**
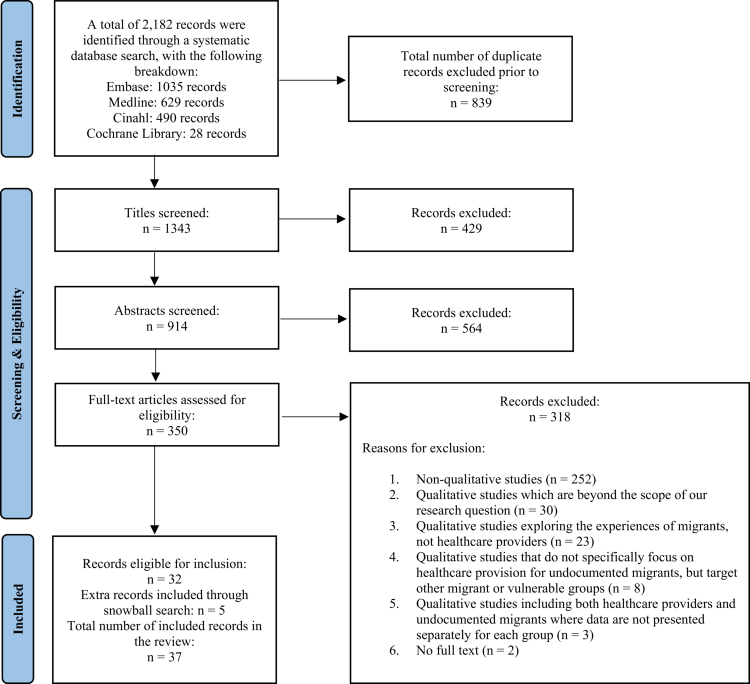
PRISMA flow chart illustrating the process of identifying and selecting relevant articles from four electronic databases.

As depicted in Figure 2, our systematic search initially identified 2,182 records. Following a screening process based on predefined inclusion and exclusion criteria, 2,150 records were excluded. This resulted in 32 qualitative studies meeting the inclusion criteria. Furthermore, the snowball search identified an additional five records, bringing the total number of studies included in the systematic review to 37.

As detailed in Table III, our systematic review included 37 qualitative studies published between 2009 and 2024. These studies were conducted across various geographic regions based on the United Nations Geoscheme classification. There were 12 studies from Northern America (United States), 16 from Europe (Western Europe: Spain [4], Belgium [2], France [1], Germany [1]; Northern Europe: Denmark [2], England [2], the Netherlands [2], Norway [2], Sweden [2]), 2 from Central America (Costa Rica), 1 from Western Asia (Israel), and 1 from Southeast Asia (Thailand). Sample sizes of participating healthcare providers varied, with 18 studies involving 1–20 participants, 16 involving 21–40 participants, and 3 involving more than 40 participants. In eight studies, the sample encompassed both healthcare providers and undocumented migrants. However, our analysis specifically focused on data derived from healthcare providers.

**Table III.** Characteristics and quality assessment of qualitative studies included in our systematic review (*n* = 37), with professional categorisation of interviewed healthcare providers (*n* = 1059).

First: Qualitative studies		
Study characteristics and quality assessment	No. of studies	(%)
**Year of publication**		
2009–2015	13	35.1
2016–2020	9	24.3
2021–2024	15	40.5
**Location**		
United States	12	32.4
Spain	4	10.8
Multiple locations	3	8.1
Belgium	2	5.4
Costa Rica	2	5.4
Denmark	2	5.4
England	2	5.4
The Netherlands	2	5.4
Norway	2	5.4
Sweden	2	5.4
France	1	2.7
Germany	1	2.7
Israel	1	2.7
Thailand	1	2.7
**Sample size**		
1−20	18	48.6
21−40	16	43.2
>40	3	8.1
**Methods of data collection**		
Interviews (semi-structured)	13	35.1
Ethnographic fieldwork (direct observation & interviews)	12	32.4
Interviews (both in-depth & semi-structured)	5	13.5
Interviews (in-depth)	3	8.1
Semi-structured interviews & focus group discussions	2	5.4
In-depth interviews & focus group discussions	1	2.7
Focus group discussions	1	2.7
**Health sector**		
Public sector (formal or governmental)	15	40.5
Private sector (non-governmental or humanitarian)	12	32.4
Both (governmental & humanitarian)	9	24.3
Unspecified	1	2.7
**Health field**		
Interdisciplinary	10	27.0
Unspecified	7	18.9
Maternal health	6	16.2
Mental health	3	8.1
Emergency medicine	3	8.1
Primary care	3	8.1
General practice	1	2.7
Child health	1	2.7
Oral health	1	2.7
Oncology	1	2.7
Organ transplantation	1	2.7
**Quality assessment**		
High	27	73.0
Good	8	21.6
Moderate	2	5.4
**Second: Healthcare providers**		
Categorisation of healthcare providers by profession	No. of providers	(%)
**Profession**		
Unspecified	499	47.1
Nurses, midwifes	134	12.7
Physicians, clinicians	130	12.3
Administrators, senior executives, service representatives or specialists	109	10.3
Mental health professionals and experts	51	4.8
NGO and CSO staff, first aid workers	46	4.3
Community health workers, social workers, early childhood specialists	37	3.5
Physician assistants, nurse assistants, medical evaluation or clinical assistants	30	2.8
Dentists	9	0.8
Cultural mediators	8	0.8
Medical students	5	0.5
Dietitians	1	0.1

Note: The sample size from one study was excluded from the overall count of healthcare providers in our systematic review. This was because the study did not explicitly specify the number of healthcare providers involved, instead reporting a combined sample size for both interviewed healthcare providers and undocumented migrants.

Data collection methods were predominantly semi-structured interviews (13 studies) and ethnographic fieldwork (12 studies). The studies explored healthcare provision for undocumented migrants in various health sectors, with 15 focused on public health systems. The studies covered a range of health fields such as interdisciplinary care (10 studies) and maternal health (6 studies). Quality assessments rated 27 studies as high quality. Additionally, the review incorporated perspectives from 1,059 healthcare providers, with a professional breakdown that included 134 nurses and midwives, 130 physicians and clinicians, and 109 administrators and service specialists. Unspecified roles accounted for nearly half of the provider sample (499). Among the included studies, six focused exclusively on nurses, and three solely on physicians.

### Main findings

**Figure 3.**
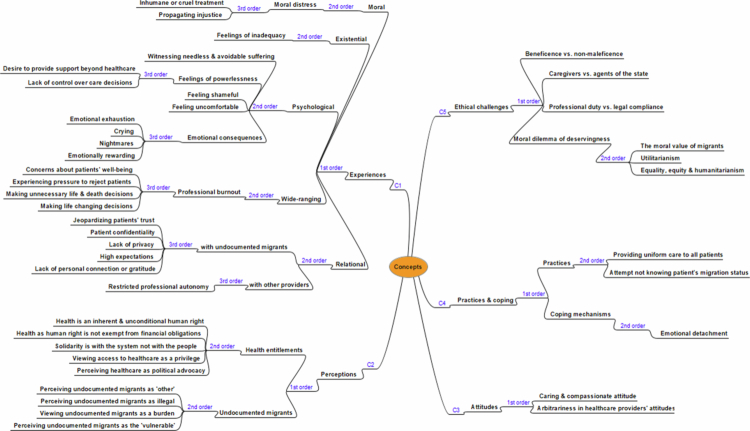
Overview of the five synthesized concepts and their associated themes and subthemes.

Our QUAGOL-guided analysis identified five main concepts: experiences, perceptions, attitudes, practices and coping mechanisms, and ethical challenges. These concepts were reformulated into guiding questions and used to structure the presentation of our findings. A total of 58 themes and subthemes emerged, categorised as 15 first-order themes, 25 second-order themes, and 18 third-order subthemes. These were organised and linked under the five major concepts. For a complete list, please refer to Figure 3. In this review, the term concepts is used to denote higher-order analytic constructs rather than individual themes. While themes and subthemes represent patterns identified directly from the empirical material, the five concepts reflect a subsequent level of analytic abstraction that integrates multiple first-, second-, and third-order themes across studies. These concepts capture broader, relational dimensions of healthcare providers’ experiences and ethical challenges that cannot be adequately represented by individual themes alone. As such, the concepts function as synthesised findings that organise and connect thematically derived evidence, and they provide the analytic building blocks for the integrative conceptual framework.

**Concept 1—Experiences: what various experiences do healthcare providers face in delivering care to undocumented migrants, and how do these experiences impact their moral character as well as their professional and personal well-being?**

Healthcare providers' experiences in delivering care to undocumented migrants were diverse, encompassing moral, existential, psychological, wide-ranging, and relational dimensions. These experiences often emerged from the significant challenges providers faced when striving to deliver equitable care within structurally inequitable systems.

### Moral experience

In two studies, a number of healthcare providers disclosed experiencing moral distress as a result of being exposed to morally stressful situations or events beyond their control (Armin, 2019; Cervantes et al., 2018). This distress was caused by their inability to prevent the transgression of undocumented migrants' entitlement to standardised equitable care. They found themselves unwillingly propagating injustices or even engaging in actions they considered inhumane or cruel (Cervantes et al., 2018). Providers also experienced moral distress when attempting to reconcile policies that restrict patient access to care with their ethical obligation to provide equitable care. This distress stemmed from structural inequities and the social exclusion embedded in public health programmes and administrative procedures (Armin, 2019).

### Existential experience

Several healthcare providers experienced a professional identity crisis when they were unable to provide necessary or adequate care. This situation led to deeply personal and often profound reflections about their roles and existence as healthcare providers. Their feelings of personal inadequacy were particularly pronounced in humanitarian and voluntary care settings, where they faced work overload, time constraints, patient privacy issues, and a lack of resources (Armin, 2019; Sandblom & Mangrio, 2017; Tiedje & Plevak, 2014).

### Psychological experience

In four studies, numerous healthcare providers reported witnessing needless and avoidable suffering among their undocumented migrant patients (Cervantes et al., 2018; Holmes, 2012; Sahraoui, 2020; Vanobberghen et al., 2022). Sometimes, this occurred on a daily basis and often involved patients they knew personally. For instance, clinicians who provide emergency-only haemodialyses to undocumented migrants with end-stage kidney disease expressed deep distress over their patients' avoidable symptoms, such as pruritus, oedema, and shortness of breath, which persisted until they met the criteria for emergency care. Many of these patients even arrived in cardiac arrest and subsequently died (Cervantes et al., 2018). Similarly, healthcare providers who volunteered to medically monitor a hunger strike organised by undocumented migrants recounted being exposed to numerous stories of suffering. These narratives included homelessness, lack of access to legal employment, chronic untreated medical conditions, and exploitation (Vanobberghen et al., 2022). Some providers even noted that prolonged exposure to such suffering caused them to feel increasingly dehumanised (Cervantes et al., 2018; Sahraoui, 2020).

In addition to witnessing their patients' suffering, several healthcare providers experienced feelings of powerlessness (Armin, 2019; Cervantes et al., 2018; Gely et al., 2023; Gullberg & Wihlborg, 2014; Holmes, 2012; Jensen et al., 2011; Kvamme & Voldner, 2022; Marrow, 2012; Tiedje & Plevak, 2014; Vanobberghen et al., 2022). They expressed their frustrations with phrases such as “not doing much” (Vanobberghen et al., 2022), “hands are tied” (Marrow, 2012), “do not have the power” (Armin, 2019), and “could not help” (Armin, 2019). Some providers even described this sense of powerlessness as a state of paralysis (Vanobberghen et al., 2022). In many cases, healthcare providers were unable to prescribe medications or arrange hospital admission (Jensen et al., 2011), provide kidney and oncology care (Armin, 2019; Cervantes et al., 2018; Gely et al., 2023), and register patients for liver transplantation or support patients with cardiac arrhythmia in receiving defibrillators (Marrow, 2012), among other critical situations, due to the patients' immigration status.

The sense of powerlessness experienced by healthcare providers arose from their desire to offer support beyond medical care (Gullberg & Wihlborg, 2014; Kvamme & Voldner, 2022; Marrow, 2012; Tiedje & Plevak, 2014; Vanobberghen et al., 2022). For example, they sought to assist in delaying the deportation of patients and their families (Kvamme & Voldner, 2022), aid undocumented migrants in accessing social support services such as unemployment benefits, disability assistance, or public housing (Marrow, 2012), and contribute to mitigating the social, political, and cultural exclusions faced by undocumented migrants (Tiedje & Plevak, 2014). Additional factors contributing to this sense of powerlessness included a lack of control over care decisions (Cervantes et al., 2018), and a feeling of perplexity due to institutional and policy constraints (Gely et al., 2023; Jensen et al., 2011).

Beyond feelings of powerlessness, some healthcare providers experienced added discomfort and shame when treating undocumented migrants. The discomfort stemmed from questioning the legitimacy of the migrants' need to remain in host countries illegally (Jensen et al., 2011). The shame originated from the moral and emotional burden associated with navigating the complexities of their professional roles (Cervantes et al., 2018; Willen, 2011). For instance, one social worker, who had to deliver distressing news, expressed “I feel like I have to go take a shower… I feel really stinky for having to tell them what I just had to tell them. They’re crying, or their family is crying” (Cervantes et al., 2018). This statement reflects a desire to cleanse oneself of the negative emotions and moral burden associated with such challenging encounters.

Caring for undocumented migrants proved to be overwhelming. Numerous healthcare providers reported that the emotional toll of caring for undocumented migrants led to various psychological consequences, including emotional exhaustion, stress, and experiences of crying and nightmares (Armin, 2019; Cervantes et al., 2018; Granero-Molina et al., 2021; Gullberg & Wihlborg, 2014; Holmes, 2012; Sahraoui, 2020; Vanobberghen et al., 2022). These effects were particularly intense for providers working in emergency care, detention centres, humanitarian settings, and with children of undocumented migrant parents. Despite these challenges, two studies found that some healthcare providers described caring for undocumented migrants as emotionally rewarding. This was because it allowed them to engage in virtuous deeds, notably helping vulnerable patients who might otherwise receive no assistance (Lafaut, 2021), and provided them with joy and satisfaction in saving their patients' lives (Tiedje & Plevak, 2014).

### Wide-ranging experience

This captures impacts extending beyond immediate psychological strain, affecting multiple domains of providers' lives and overall well-being. In this context, numerous healthcare providers reported experiencing professional burnout, characterised by elevated stress levels, profound frustration, and physical exhaustion related to their duties. Many described it as an ongoing, repetitive cycle (Armin, 2019; Cervantes et al., 2018; Sahraoui, 2020; Willen, 2011).

The professional burnout experienced by healthcare providers was primarily driven by their deep concern for their patients' well-being (Cervantes et al., 2018; Sahraoui, 2020). Many providers recounted instances where they were under intense pressure to deny care or turn away medically unstable patients. These circumstances often forced them to make critical, life-or-death decisions—choices that could have been avoided if undocumented migrants had access to comprehensive healthcare beyond just emergency services (Cervantes et al., 2018). In some instances, healthcare providers became the sole decision-makers, wielding significant power over the lives of undocumented migrants and bearing the heavy burden of decisions with far-reaching consequences, such as determining whether a migrant’s health condition was compatible with deportation (Sahraoui, 2020). Additional factors contributing to professional burnout included the administrative invisibility and systemic exclusion of undocumented migrants from healthcare systems and policies, which placed an undue burden on healthcare providers, making them solely responsible for addressing the health needs of this vulnerable population (Armin, 2019).

### Relational experience

By “relational experience,” we mean the interactions healthcare providers have with both undocumented migrants and their colleagues, and how these interactions shape their overall experiences. When engaging with undocumented migrants, healthcare providers' experiences were significantly shaped by factors such as trust, confidentiality, privacy, expectations, and gratitude.

In the context of the provider-patient relationship, some healthcare providers expressed concerns about jeopardising their patients' trust. Trust was diminished as both undocumented migrants and providers found themselves in challenging situations where undocumented migrants were unable to access the necessary and deserved healthcare, and providers were unable to deliver the level of care they were committed to providing (Cervantes et al., 2018). This lack of trust was particularly pronounced among healthcare providers working in detention centres, where their involvement in migration control processes introduced an element of suspicion into their relationships with undocumented migrants (Sahraoui, 2020).

Patient confidentiality is another issue that significantly influenced healthcare providers' interactions with undocumented migrants (Biswas et al., 2011; Dauvrin et al., 2012; Jensen et al., 2011; Straßmayr et al., 2012; Willen, 2011). Given the precarious legal status of these individuals, some providers faced uncertainty regarding how to uphold confidentiality, often grappling with the dilemma of whether to report undocumented patients, particularly in the absence of explicit policies or guidelines (Biswas et al., 2011). This quandary was especially pronounced for providers caring for undocumented migrants with severe mental health conditions. In extreme scenarios, such patients might be referred to hospitals for observation or treatment without their consent, heightening the risk of detection by immigration authorities and subsequent deportation once stabilised (Straßmayr et al., 2012).

Healthcare providers also indicated a tendency to report undocumented migrants under specific circumstances: when they were suspected of involvement in criminal activities or posed a danger to themselves or others, when they were severely injured, or in the event of a fatality during treatment—particularly when it was necessary to inform their relatives or when certain identification challenges arose (Dauvrin et al., 2012; Jensen et al., 2011). While some providers were uncertain about how to navigate the confidentiality of patients, others expressed a firmer stance, asserting that hospitals should be considered sanctuaries where patients can seek care without fear, and emphasising that access to healthcare should be independent of a migrant’s legal status (Jensen et al., 2011).

In addition to upholding patient confidentiality, several healthcare providers identified the lack of privacy within the provider-patient relationship as a significant challenge, particularly in humanitarian and voluntary healthcare settings (Granero-Molina et al., 2021, 2022; Saadi et al., 2021; Vanobberghen et al., 2022). This challenge was further intensified when immigration and law enforcement officers were present during patient consultations (Saadi et al., 2021). For certain vulnerable populations, such as victims of human trafficking among undocumented migrant women, the absence of privacy was emphasised as a critical barrier to effective healthcare, as it hinders providers' ability to establish trust with their patients (Granero-Molina et al., 2022).

Other factors that affected the relationship between healthcare providers and undocumented migrants included the migrants' high expectations of the providers and a perceived lack of gratitude from the migrants. One study reported on these elevated expectations and highlighted that undocumented migrant mothers and families believed the public health nurses could influence authorities regarding their permanent residency status (Kvamme & Voldner, 2022). The issue of a lack of gratitude was mentioned in another study, where some dentists offering free oral care to undocumented migrants expressed feeling unappreciated and experienced a lack of personal connection with their patients (van Midde et al., 2021).

In the context of inter-provider relationships, some nurses reported instances where their professional autonomy was curtailed by physicians or specialists, particularly concerning the referral of undocumented migrants to hospitals, the prescription of medications, and interactions with immigration authorities (Granero-Molina et al., 2022; Saadi et al., 2021). For example, during humanitarian response efforts, when undocumented migrants required antibiotics or painkillers, but their cases were not deemed urgent, physicians often withheld approval for hospital transfers. This posed significant challenges for nurses, who were not authorised to prescribe medications. Despite these restrictions on their autonomy, nurses also described situations where their responsibilities expanded to include emergency care in the absence of physicians. However, even in these circumstances, they were still not permitted to prescribe medications or transfer patients without a physician’s authorisation (Granero-Molina et al., 2022).

**Concept 2—Perceptions: how do healthcare providers perceive undocumented migrants and their health entitlements, and what factors contribute to these perceptions?**

The perceptions of healthcare providers regarding undocumented migrants and their health entitlements were often polarised, reflecting not only individual beliefs but also broader societal debates about the intersection of health, immigration status, and human rights.

### Health entitlements

Healthcare providers expressed divergent perspectives regarding health as a human right. Some viewed the health entitlements of undocumented migrants as an inherent and unconditional right, irrespective of their immigration status, legal standing, insurance coverage, or ability to pay (Bianchi et al., 2019; Hoekstra, 2021; Marrow, 2012; Tiedje & Plevak, 2014; Tschirhart et al., 2021). Others, however, argued that the notion of health as a human right does not absolve individuals from financial obligations, particularly for critically ill patients or those requiring costly medical procedures or interventions (Armin, 2019; Dos Santos 2015; Tschirhart et al., 2021). Even in the presence of charity organisations or clinics, such as mercy clinics providing cancer care, these facilities often lack the capacity to care for everyone, and the funds available are insufficient to cover the expenses of high-cost treatments like chemotherapy (Armin, 2019).

One provider perceived health as a commodity, arguing that undocumented migrants should be held accountable when utilising free medical services (Bianchi et al., 2019). Two providers even linked the high fertility rate among undocumented migrant women to their free access to maternal services, proposing that the number of free births available to these women should be limited (Bianchi et al., 2019). In a similar vein, some healthcare providers expressed greater solidarity with healthcare systems than with undocumented migrants, justifying this stance by emphasising the need to protect healthcare systems from potential collapse (Bianchi et al., 2019; Dos Santos, 2015; Goldade, 2009; Gullberg & Wihlborg, 2014; Marrow, 2012). Furthermore, some providers viewed access to healthcare services for undocumented migrants as a privilege, citing examples such as undocumented migrant women crossing borders to give birth (Dos Santos, 2015; Goldade, 2009), and those receiving medical services funded by taxpayers (Bianchi et al., 2019; Marrow, 2012).

Despite these varied perceptions, several other providers viewed the provision of healthcare to undocumented migrants as a form of political advocacy, aimed at countering hostile anti-migrant environments, promoting health justice, and upholding human dignity (Castañeda, 2011; Hoekstra, 2021; Piccoli & Perna, 2024; Willen, 2011). These providers highlighted the importance of raising awareness about the challenges faced by undocumented migrants through storytelling, advocacy campaigns, petitions, and media coverage. Additionally, they emphasised the need for lobbying policymakers by leveraging their expertise, data, and resource mobilisation to support these advocacy efforts (Piccoli & Perna, 2024).

### Undocumented migrants

In examining healthcare providers' perceptions of undocumented migrants, it was found that some providers harboured unfavourable views. Certain providers regarded undocumented migrants as “others”, emphasising their cultural differences in a judgmental manner (Dos Santos, 2015; Goldade, 2009). Some perceived undocumented migrants as illegal, immoral, or irrational, partially blaming them for their precarious living situations or poor health outcomes (Bianchi et al., 2019; Dos Santos, 2015; Worthing et al., 2022). A minority of providers characterised migrants, particularly undocumented pregnant women, as demanding (Goldade, 2009). One provider even expressed an extreme view by labelling undocumented migrants as intruders (Dos Santos, 2015).

In three studies, some healthcare providers viewed undocumented migrants as a burden on healthcare systems due to the high costs associated with their care, their complex health needs, and the additional time and effort required to treat them (Gely et al., 2023; Goldade, 2009; Worthing et al., 2022).

Healthcare providers' perspectives on vulnerability were also contradictory. While some recognised the vulnerability of undocumented migrants due to their challenging circumstances (Gullberg & Wihlborg, 2014; Willen, 2011), others perceived themselves as vulnerable, feeling burdened by what they described as the rudeness of undocumented migrants (Dos Santos, 2015).

**Concept 3—Attitudes: what attitudes do healthcare providers exhibit towards undocumented migrants, and what factors influence their interactions?**

The attitudes of healthcare providers towards undocumented migrants were varied, encompassing both compassionate and arbitrary elements. Many healthcare providers demonstrated a caring and empathetic approach, striving to offer culturally sensitive care and advocating for the health entitlements of undocumented migrants. However, these attitudes were not always consistent. The arbitrariness in their behaviour was evident in instances where personal biases, stereotypes, or administrative challenges influenced their interactions, leading to varied and sometimes conflicting responses.

### Caring and compassionate attitude

When delivering care to undocumented migrants, numerous healthcare providers demonstrated compassionate and empathetic attitudes (Armin, 2019; Bianchi et al., 2019; Castañeda, 2011; Cervantes et al., 2018; Doshi et al., 2020; Gely et al., 2023; Granero-Molina et al., 2021; Jiménez-Lasserrotte et al., 2023; Kvamme & Voldner, 2022; Mladovsky, 2023; Piccoli & Perna, 2024; Saadi et al., 2021; Sandblom & Mangrio, 2017; Straßmayr et al., 2012; Teunissen et al., 2015; Tiedje & Plevak, 2014; Vanobberghen et al., 2022; Voldner et al., 2023; Willen, 2011). This included adopting a welcoming demeanour, being consistently available for undocumented migrants, offering additional support that went beyond medical care, and advocating for undocumented migrants’ health entitlements.

Healthcare providers also underscored the significance of empathy in fostering connections with undocumented migrants (Vanobberghen et al., 2022), aiming to challenge existing stereotypes (Jiménez-Lasserrotte et al., 2023), and gain a deeper understanding of their experiences and emotions (Granero-Molina et al., 2021, 2022; Jiménez-Lasserrotte et al., 2023; López-Domene et al., 2019; Vanobberghen et al., 2022). This approach, in turn, facilitates the provision of more culturally sensitive care. Nonetheless, a study revealed that some midwives involved in migration control faced difficulties in sustaining their initial empathetic approach toward undocumented migrants (Sahraoui, 2020). For instance, one midwife initially sought any minor justification to prevent the deportation of undocumented migrants, but after two years in the role, she eventually complied with sending them back based on medical criteria (Sahraoui, 2020).

### Arbitrariness in healthcare providers’ attitudes

Although healthcare providers exhibited compassionate attitudes, these attitudes were marked by inconsistency and were at times unpredictable. In some situations, they were sympathetic and supportive, while in others, they appeared indifferent. For instance, some healthcare providers displayed a lack of compassion in their interactions with undocumented migrants, such as by requesting documents and information that these individuals could not provide (Granero-Molina et al., 2022). In some cases, this lack of compassion was influenced by personal biases and judgements (Goldade, 2009; Marrow, 2012). Certain providers even blamed undocumented migrants, particularly pregnant women, for excessive reproduction, neglecting prenatal care, and thereby endangering their unborn children (Bianchi et al., 2019; Dos Santos, 2015).

Rather than showing cultural sensitivity, some providers attributed poor health conditions to the religious and cultural beliefs of the migrants, holding them responsible for what they perceived as irresponsible behaviours (Holmes, 2012). Additionally, some healthcare providers expressed concerns that undocumented migrants could pose a risk to themselves, other patients, or staff, although these concerns appeared to be based on hypothetical scenarios rather than direct experiences (Worthing et al., 2022). Lastly, there were instances where undocumented migrants were administratively invisible to certain doctors and specialists, who subsequently turned a blind eye to this issue (Armin, 2019; Worthing et al., 2022).

**Concept 4—Practices & Coping: what practices and coping mechanisms do healthcare providers employ when delivering care to undocumented migrants, and how do these strategies influence their ability to balance professional responsibilities with personal norms and resilience?**

Healthcare providers employed varied practices and coping mechanisms when delivering care to undocumented migrants. Providers often adopted practices that emphasised equality, such as offering uniform treatment to all patients. Their coping mechanisms ranged from emotional detachment to maintaining a strictly medical focus in their interactions, revealing the complex interplay between professional responsibilities and personal resilience in the face of challenging circumstances.

### Practices

Several healthcare providers implemented the practice of offering uniform care to all patients, regardless of citizenship, documentation, or insurance status (Bianchi et al., 2019; Fabi & Taylor, 2019; Jensen et al., 2011; Saadi et al., 2021; Yu et al., 2020). This approach often aligned with the healthcare providers' personal norms and values, particularly when these were in harmony with the mission of their institutions (Fabi & Taylor, 2019). Providers who adopted this practice justified it as an ethical imperative rooted in the principles of equality and health justice (Bianchi et al., 2019; Fabi & Taylor, 2019), as well as from a utilitarian perspective (Castañeda, 2011; Saadi et al., 2021).

In some instances, healthcare providers consciously avoided knowing the migration status of their patients (Armin, 2019; Fabi & Taylor, 2019; Lafaut, 2021; Marrow, 2012; Saadi et al., 2021; Yu et al., 2020). One provider expressed that having less knowledge about a patient’s legal status felt safer and more effective (Armin, 2019). Other providers emphasised that this approach not only facilitates the delivery of high-quality and equitable care (Yu et al., 2020), but also fosters trust, alleviates fears of deportation, and makes undocumented migrants feel more at ease (Fabi & Taylor, 2019; Marrow, 2012). Even when healthcare providers were aware of a patient’s undocumented status, they refrained from labelling them as such, opting instead for categorisations that avoided emphasising “undocumentedness” (Lafaut, 2021; Marrow, 2012). For example, they categorised undocumented migrants as patients with limited access to healthcare, similar to other vulnerable groups such as the homeless, and focused on integrating these patients into available care systems (Lafaut, 2021).

One study underscored the challenge of overlooking the differences that set migrants apart from other patients when providing healthcare. Nevertheless, healthcare providers made deliberate efforts to maintain neutrality in their interactions, striving to treat all patients equitably despite these differences (Lafaut, 2021).

### Coping mechanisms

As indicated in the preceding findings, delivering healthcare to undocumented migrants can be emotionally exhausting, and relationally and morally challenging, particularly when providers witness preventable suffering and experience powerlessness under institutional and policy constraints. To cope and protect themselves, some providers adopted strategies of emotional detachment (Cervantes et al., 2018; Sahraoui, 2020; Vanobberghen et al., 2022).

However, this detachment can sometimes deviate from empathetic care, potentially harming the provider-patient relationship. For instance, one provider, who offers emergency-only haemodialyses to undocumented migrants, explained that it is easier to view these patients as mere numbers—focusing on metrics such as potassium, oxygen, and bicarbonate levels—rather than as individuals, in reference to undocumented migrants who meet the criteria for emergent or life-threatening conditions (Cervantes et al., 2018).

Other providers used phrases like “being objective”, “making technical decisions”, and “keeping consultations strictly medical” to describe their approach to managing the emotional strain of treating undocumented migrants, emphasising a focus on medical aspects rather than personal connections (Sahraoui, 2020). By framing their work in technical terms and limiting emotional engagement, providers sought to reduce distress and preserve functional capacity in demanding settings. In certain healthcare settings, such as detention centres, this emotional detachment has fostered mutual suspicion between healthcare providers and undocumented migrants, thereby hindering the development of genuine care relationships (Sahraoui, 2020).

**Concept 5—Ethical challenges: what ethical challenges do healthcare providers face when delivering care to undocumented migrants, and in which situations do their duty to care and their professional obligations create moral conflicts?**

Healthcare providers encountered a range of ethical challenges that often placed them in moral conflict. These challenges primarily revolved around balancing the principles of beneficence and non-maleficence, as providers strived to deliver care that benefited their patients but were sometimes constrained by institutional and legal limitations, inadvertently causing harm. Moreover, their dual roles as caregivers and agents of the state created tensions between their duty to care and their role in migration control.

Healthcare providers also faced conflicts between their professional duties toward their patients and legal compliance with governmental and institutional regulations. Finally, the moral dilemma of deservingness further complicated decision-making, as providers navigated contrasting frameworks to determine who was entitled to care. These ethical tensions highlighted the complexity of care delivery in such contexts, where both professional and moral obligations were continuously tested.

### Beneficence vs. non-maleficence

In this systematic review, we identified various practices employed by healthcare providers to act in the best interest of undocumented migrants and uphold the principle of beneficence, such as offering equal care to all patients regardless of their immigration status and advocating for the health entitlements of undocumented migrants. However, providers also encountered circumstances where, despite their intentions, they inadvertently caused harm. For example, some found themselves in situations where they had to deny essential or life-saving treatments and services (Cervantes et al., 2018; Fabi & Taylor, 2019). One case involved the denial of emergency-only haemodialyses, even for visibly ill patients, particularly when dialysis chairs were unavailable. One provider expressed the gravity of such decisions by remarking “It just seems like we are playing Russian roulette to some extent with people’s lives” (Cervantes et al., 2018).

Several healthcare providers also recounted instances where care was modified based on patients’ immigration status, leading to unequal treatment (Cervantes et al., 2018; Fabi & Taylor, 2019; Gullberg & Wihlborg, 2014). For instance, a diabetic patient with deteriorating eyesight received laser treatment while classified as an asylum-seeker, but this care was discontinued after the patient’s asylum claim was rejected, as their condition was deemed non-urgent (Gullberg & Wihlborg, 2014). Moreover, harmful practices often stemmed from providers’ stereotypical attitudes and perceptions toward undocumented migrants, resulting in differential treatment and substandard care for this vulnerable population (Gullberg & Wihlborg, 2014; Holmes, 2012; Jiménez-Lasserrotte et al., 2023; Worthing et al., 2022).

These stereotypical perceptions included ethnocentric assumptions about migrant patients and the misinterpretation of their cultural practices, leading to incorrect attributions of poor health outcomes to the migrants’ cultural beliefs (Holmes, 2012). Stereotypical thinking extended further, encompassing ideas of ethnic body differences. For instance, a retired dentist claimed that Mexican migrant patients have “granite-like” bone structures, making dental procedures more difficult (Holmes, 2012). We find that such instances of biological essentialism reduce patients to simplistic ethnic stereotypes, attributing a physical characteristic to an entire ethnic group. This assumes that their dental health issues are inherently tied to their ethnicity, without considering other factors such as access to care or nutrition. In another study, some healthcare providers expressed concerns that undocumented patients might conceal their identity or past, potentially posing safety risks. Although these scenarios were often hypothetical, they reflect stereotypical fears that undocumented migrants might be hiding criminal backgrounds or engaging in dangerous behaviour (Worthing et al., 2022).

### Caregivers vs. agents of the state

In two ethnographic studies, healthcare providers found themselves navigating a tension between their professional responsibilities as caregivers and their institutional roles as agents of the state. This ethical conflict was particularly evident in contexts where providers were involved in migration control or bureaucratic triage that limited access to healthcare (Goldade, 2009; Sahraoui, 2020).

In one study, midwives working in detention centres during the deportation of undocumented migrants faced significant ethical challenges. They were simultaneously responsible for safeguarding the health of pregnant detainees while participating in the broader biopolitical regulation of migrants’ mobility. The presence of law enforcement personnel during medical care further compromised the midwives’ autonomy and their ability to prioritise patient well-being, resulting in feelings of powerlessness and complicity (Sahraoui, 2020).

In the other study, healthcare providers—particularly social workers and receptionists—assumed bureaucratic gatekeeping roles that directly affected migrants' access to healthcare. Administrative staff were tasked with verifying patients’ insurance documentation before allowing access to services. This bureaucratic process required non-medical staff to deny care to uninsured undocumented migrants, often before any clinical evaluation could occur. As a result, the ethical obligation to provide care was subordinated to institutional protocols, effectively positioning some providers as enforcers of exclusionary state policies (Goldade, 2009).

### Professional duty vs. legal compliance

Professional duty refers to the ethical obligations that healthcare providers hold toward patients, irrespective of their legal status. These duties encompass delivering care that safeguards patient well-being and respects human dignity. On the other hand, legal compliance pertains to following the laws and regulations established by governments or institutions, which may include restrictions on healthcare services for undocumented migrants and, in extreme cases, the requirement to report their status.

In the context of providing healthcare to undocumented migrants, many providers encountered situations where their professional obligations conflicted with legal requirements or regulations (Castañeda, 2011; Fabi & Taylor, 2019; Saadi et al., 2021; Straßmayr et al., 2012). For example, some healthcare providers described instances where they leveraged informal networks to circumvent restrictions, choosing to treat undocumented migrants despite the risk of contravening legal mandates. These decisions were often driven by personal conscience (Straßmayr et al., 2012).

This conflict created significant ethical challenges, as healthcare providers were forced to balance their moral duty to care for all patients with legal frameworks that could restrict their ability to do so. The challenge became even more acute in situations where medical humanitarianism for undocumented migrants was criminalized, placing healthcare providers at risk of legal consequences for assisting these individuals (Castañeda, 2011; Straßmayr et al., 2012). Even in cases where such criminalisation did not lead to convictions, it fostered a perception among healthcare providers and non-governmental organisations that they were operating in a legal grey area (Castañeda, 2011). One provider highlighted this dilemma, stating “The healthcare for illegal immigrants is mostly provided underground or by NGOs which do it on the conditions that is illegal according to the country’s legislation” (Straßmayr et al., 2012).

### Moral dilemma of deservingness

This dilemma captures the ethical tensions healthcare providers face when determining who is entitled to healthcare. Providers’ judgements on deservingness are shaped by complex and sometimes conflicting moral frameworks—ranging from moral judgements to utilitarian approaches, and equality- and equity-driven care. First, under the moral value of migrants, some providers question migrants' deservingness based on their perceived moral standing, often linking it to notions of citizenship and societal contribution. These judgements are influenced by resource scarcity and perceived moral character.

In contrast, the utilitarian perspective emphasises the broader societal benefits of providing healthcare to all, arguing that preventive and primary care for undocumented migrants is a cost-effective approach to protect public health and reduce the strain on emergency services. Finally, the framework of equality, equity, and humanitarianism highlights a more inclusive view, where providers advocate for healthcare access as a fundamental human right, stressing fairness and the moral duty to address health disparities through compassionate and equitable care.

### The moral value of migrants

Undocumented migrants were depicted by some healthcare providers as morally inferior and less entitled to healthcare benefits, based on criteria such as citizenship or societal contribution (Dos Santos, 2015; Goldade, 2009; Worthing et al., 2022). In some instances, this portrayal arose from viewing undocumented migrants as “the other”, with judgements about their moral character underpinning providers’ beliefs about their deservingness of medical care (Dos Santos, 2015; Goldade, 2009). For example, one provider remarked “We know that they live in a precarious situation, and that they face a difficult political situation [in Nicaragua], but they come and they demand”; “If I have a house and the cousins come over, cousins that come to destroy the house, I have to close the doors and tell them I love them but I have to protect my home. Right?” (Dos Santos, 2015). Other providers expressed concerns that undocumented migrants were fraudulently exploiting resources meant for those deemed more “deserving”, especially given the scarcity of available services (Worthing et al., 2022). One provider shared “There’s people coming from all over the world, who can come here, get treatment, lie through their teeth, owe the NHS thousands of pounds, and you’ve got genuine people who have worked all their lives and can’t get any treatment, got to wait months or years for it. It does hurt I am afraid” (Worthing et al., 2022).

This perception of undocumented migrants as less deserving was not only voiced by some healthcare providers but also by legal citizens who felt that undocumented migrants were unjustly receiving publicly-funded healthcare, often at their own expense (Marrow, 2012). In response to such sentiments, a nurse explained to her family—legal descendants of Eastern European immigrants—that “we all need healthcare”, and that “healthcare doesn’t know papers” (Marrow, 2012). Additionally, some healthcare providers expressed a strong personal commitment to serving undocumented migrants, viewing them as deserving of care and appreciating the opportunity to work with them (Holmes, 2012). Others emphasised the economic contributions of undocumented migrants, as one nurse noted “Our society would not function without [unauthorised] people working for nothing and paying taxes and not getting any services” (Marrow, 2012).

In one study, healthcare providers' decisions to treat undocumented women were often influenced by moral considerations regarding the foetus. The primacy given to the foetus frequently ensured medical care; however, the women themselves were often criticised for being “marginal” and for lacking prenatal care. As Dos Santos explains, this reflects the invocation of two distinct moral frameworks: the foetus, perceived as innocent and divinely protected, elicited compassion, while the mothers, viewed as guilty, provoked judgement (Dos Santos, 2015). In another study, some healthcare providers considered undocumented pregnant women deserving of healthcare due to the heightened risks associated with pregnancy, given that both the mother and foetus were at stake. These providers emphasised the importance of prenatal care for the well-being of both mother and child, highlighting the humanity of the infant and portraying the mother as the caretaker of a future citizen (Bianchi et al., 2019).

### Utilitarianism

Several healthcare providers justified the deservingness of healthcare access for undocumented migrants from a utilitarian standpoint, particularly emphasising the importance of primary and preventive healthcare services (Bianchi et al., 2019; Castañeda, 2011; Gely et al., 2023; Goldade, 2009; Marrow, 2012; Tiedje & Plevak, 2014). One provider, for instance, expressed “I think that everybody should get care. If you’re not going to treat someone for TB because they’re undocumented, or if you’re not going to give them birth control or treat their diabetes because “it doesn’t affect” you, [you should know] it all affects you. If you can’t take care of your community then you can’t take care of yourself” (Marrow, 2012).

Providers argued that expanding preventive and primary care for undocumented migrants is a cost-effective strategy to reduce the higher expenses associated with emergency care (Marrow, 2012). From a utilitarian perspective, many healthcare providers emphasised that restricting healthcare services for undocumented migrants would result in serious consequences and place a financial strain on healthcare systems. They underscored the importance of immunisation in preventing harmful diseases, access to adequate maternity care to avoid birth defects linked to folic acid deficiency, and effective pain management to reduce the overuse of painkillers (Bianchi et al., 2019; Tiedje & Plevak, 2014). Additional examples include family planning, cancer screenings, dental care, and other essential services (Castañeda, 2011; Goldade, 2009). Finally, a social worker highlighted the cost-effectiveness of allowing undocumented migrants to obtain health insurance, which would increase access to expensive procedures such as organ transplantation (Gely et al., 2023).

### Equality, equity and humanitarianism

Many healthcare providers viewed the concept of health deservingness for undocumented migrants through the frameworks of equality, equity, and humanitarianism. Health equality asserts that undocumented migrants should receive the same standard of care as any other individual, regardless of their legal status or socio-economic background. However, this perspective on health access overlooks the significant barriers undocumented migrants often face in obtaining healthcare. For this reason, a number of healthcare providers also viewed health deservingness through the lenses of equity and humanitarianism. These two approaches acknowledge the disparities experienced by undocumented migrants and aim to provide healthcare that addresses their specific needs and circumstances. In summary, while equality strives for sameness, equity strives for fairness by addressing health disparities.

When considering health deservingness through the lens of equality, several healthcare providers emphasised the inherent humanity of undocumented migrants, asserting that they should be treated no differently than others (Bianchi et al., 2019; Hoekstra, 2021; Piccoli & Perna, 2024). This perspective reflects the belief that the healthcare system should be inclusive and respectful of people from diverse cultures and ethnic backgrounds (Bianchi et al., 2019). One provider articulated the significance of equality by stating “Because we’re human beings. Because we’re all human beings, because how we treat others matters. And there’s nobody that’s greater or less than anyone else. Whatever the wealthiest deserves, the poorest deserves as well... That’s why” (Bianchi et al., 2019). Another provider echoed this opinion, explaining “Medical care is not a commodity that only a few should have because they have the right things in their pockets—money or papers. There cannot be a hierarchy of who gets health care and who doesn’t … or who gets good health care and who has to just get what’s left over. We should all get the same health care” (Hoekstra, 2021).

Even in instances where civil society organisations expand healthcare access for undocumented migrants, several providers emphasised the importance of including these individuals in public services as a matter of recognition. This inclusion goes beyond mere access to healthcare services; it also affirms that undocumented migrants are entitled to health rights and have the capacity to claim and exercise these rights within the healthcare system (Piccoli & Perna, 2024).

In an effort to mitigate health disparities that undocumented migrants face and promote health equity, numerous healthcare providers sought alternative avenues for delivering care, often through humanitarian initiatives, volunteer work, and personal undertakings such as voluntary networks, clinics, and partnerships with civil society organisations. The motivations driving these providers were diverse, ranging from a deep commitment to social justice to a desire to serve those most in need (Granero-Molina et al., 2021, 2022; Holmes, 2012; Jiménez-Lasserrotte et al., 2023; López-Domene et al., 2019; Piccoli & Perna, 2024; Tiedje & Plevak, 2014; Willen, 2011).

This dedication was frequently described as a personal calling to serve marginalised communities (Holmes, 2012). One provider explicitly rejected terms like “compassion” or “charity” to describe her efforts, stating “I don’t simply do an act of charity”, and emphasising her sustained commitment to the community with whom she had forged close ties over time (Tiedje & Plevak, 2014). Other providers were motivated by faith and altruism, with one remarking “For me, this is where God is calling me” (Tiedje & Plevak, 2014).

However, while humanitarian work plays a critical role in addressing the healthcare needs of undocumented migrants, concerns have been raised about the potential risk of supplementing, rather than supporting and integrating with, public healthcare systems—thus perpetuating two parallel and unequal structures of care, which may contradict the concept of health equity (Piccoli & Perna, 2024).

### Discussion

This systematic review synthesises qualitative evidence on the ethical challenges and moral stressors experienced by healthcare providers delivering care to undocumented migrants, an area that has remained fragmented and under-theorised in the existing literature. By integrating findings from diverse healthcare settings and policy contexts, the review moves beyond isolated descriptions of provider experiences to offer a structured account of how ethical challenges emerge, interact, and persist across clinical practice. The synthesis suggests that these challenges are not simply the result of individual provider uncertainty or isolated bedside dilemmas. Rather, they reflect a process of ethical burden-shifting, in which governments, institutions, or dominant policy structures transfer moral responsibility and the consequences of exclusionary arrangements onto less powerful actors. In the context of undocumented migrants’ healthcare, this occurs when access to care is shaped by migration control, limited entitlements, administrative ambiguity, and judgements about deservingness, leaving providers and patients to carry the practical and moral weight of these arrangements. Providers are consequently required to negotiate these tensions through everyday clinical judgements, advocacy efforts, informal workarounds, confidentiality decisions, emotional coping, gatekeeping, or humanitarian responses. In doing so, the review addresses an important gap in prior research, which has largely focused either on access barriers for undocumented migrants or on providers’ experiences in specific settings, without systematically examining the ethical dimensions that cut across these contexts.

The Discussion is organised into two main sections. First, we interpret the findings through an integrative conceptual framework that elucidates the relationships between providers’ experiences, perceptions, attitudes, practices, coping mechanisms, and ethical challenges. Second, we situate these findings within the broader literature and discuss their implications for ethical guidance, institutional support, and future research in migration health.

### Weaving concepts into a framework

This systematic review identified five interconnected concepts—experiences, perceptions, attitudes, practices and coping mechanisms, and ethical challenges—that collectively clarify the interrelated dynamics of healthcare providers’ roles in caring for undocumented migrants. These concepts do not exist in isolation but are deeply interwoven, offering a holistic view of the ethical landscape—that is, the constellation of moral pressures, institutional constraints, and professional obligations within which providers operate. The conceptual model in the supplementary material illustrates how experiences of moral stress are linked to perceptions of undocumented migrants and their health entitlements, which in turn shape attitudes, inform clinical practices and coping responses, and give rise to recurrent ethical conflicts under legal and institutional constraints.

The experiences of healthcare providers emerge as a foundation, revealing the profound moral stress, emotional burden, and risk of professional burnout that arise when delivering care in structurally inequitable systems. Providers’ duty to care is often constrained by institutional barriers, creating conflicts that amplify moral distress and undermine their ability to fulfil professional obligations. These experiences set the stage for shaping providers’ perceptions and attitudes.

Perceptions and attitudes, while distinct, are closely linked to these experiences. Perceptions of undocumented migrants and their entitlements are shaped by a complex interplay of personal values, societal narratives, and systemic biases. These perceptions often lead to two diverging pathways of attitudes. Some providers, perceiving health as a human right, demonstrate compassionate and patient-centred attitudes. Others, influenced by stereotypes or by framing health as a privilege, adopt arbitrary or indifferent attitudes that marginalise patients. This duality underscores how perceptions and attitudes can either promote culturally sensitive care or reinforce exclusion.

The concepts of practices and coping mechanisms reflect the strategies providers employ to navigate these challenges. On one hand, practices such as providing uniform care regardless of citizenship, documentation, or insurance status embody advocacy for migrants’ health entitlements. On the other, coping strategies such as emotional detachment and depersonalisation—though protective for providers—create relational barriers that erode the provider-patient relationship.

At the heart of these dynamics lies the concept of ethical challenges, which binds the other concepts together and directly addresses our research question. These challenges—rooted in the conflicts between beneficence and non-maleficence, professional duty versus legal compliance, and the moral dilemma of deservingness—reflect the tensions healthcare providers face as they navigate their dual roles as caregivers and agents within broader sociopolitical systems. The conceptual model in the supplementary material highlights how ethical challenges both shape and are shaped by providers’ experiences, perceptions, attitudes, and practices, revealing a cyclical interplay in which systemic barriers amplify moral distress, and moral distress, in turn, influences care delivery.

By synthesising these concepts, the review reveals that addressing these challenges requires more than individual resilience or isolated provider-level interventions. Many of the ethical tensions identified in this review are generated by restrictive entitlement regimes, institutional ambiguity, and resource constraints; therefore, responsibility for addressing them cannot rest primarily with frontline providers. Responses must therefore shift attention back to the policy and institutional levels where many of these constraints are produced and sustained. This requires systemic reforms and institutional support systems that align healthcare delivery with principles of equity, justice, and human rights. Such alignment requires translating these normative commitments into practice through clear entitlement policies, non-discriminatory administrative procedures, safeguards for confidentiality, and institutional guidance that supports healthcare providers in delivering care based on need rather than legal status.

### Expanded discussion on concepts

In this section, we explore each concept in greater depth, tracing the ways they intersect within the overarching framework.

#### Moral distress and moral injury: causes, prevalence, impacts, and support strategies

Moral experience refers to how healthcare providers perceive and respond to ethically or morally significant situations encountered in their professional lives. It involves the subjective process of grappling with moral values, ethical principles, and the practical realities of decision-making, as well as the alignment—or conflict—between these principles and the actions taken. Such experiences are profoundly influenced by professional ethics, personal beliefs, cultural norms, and situational factors.

Our analysis revealed that healthcare providers often experience moral distress when their decisions or actions conflict with their moral values and professional ethics. This tension is particularly pronounced when providers are compelled to deny patients essential care due to their immigration status, a circumstance that many perceive as perpetuating injustice. Prolonged exposure to such morally distressing situations can lead to moral injury, a more severe psychological condition.

Moral injury arises when individuals face events that deeply violate their core moral beliefs, such as perpetrating or failing to prevent ethically conflicting actions or experiencing betrayal by trusted authorities. Unlike moral distress, which is rooted in external constraints that impede ethical action, moral injury reflects an internal struggle characterised by guilt, shame, and a sense of moral failure (Griffin et al., 2019). Many of these feelings were articulated by healthcare providers in our review, emerging as recurrent themes in their narratives.

Griffin et al. identified specific contexts where healthcare providers are susceptible to moral injury, including end-of-life decisions, resource allocation during crises, and navigating ethical ambiguity in military or humanitarian settings (Griffin et al., 2019). While end-of-life decisions and military contexts were not prominent in our review, we found that healthcare providers working in emergency care, humanitarian contexts, and detention centres frequently encountered morally stressful situations. These settings often forced providers to make ethically complex decisions, further exacerbating moral distress and the risk of moral injury.

The prevalence of moral distress among healthcare providers caring for undocumented migrants had been widely documented, aligning with our findings. For instance, emergency physicians were particularly vulnerable to moral distress due to their role as gatekeepers to emergency medical services. They faced the ethical dilemma of stabilising and discharging undocumented patients, fully aware that these individuals lacked access to follow-up care and remained at risk of deterioration (Kluesner et al., 2021). Similarly, clinicians providing emergency-only haemodialyses for undocumented immigrants with end-stage renal failure reported experiencing severe moral distress. They were compelled to participate in a system that withheld treatment until life-threatening conditions arose—a practice viewed as inhumane and ethically problematic. Notably, 48% of clinicians reported experiencing such distress, with 73% identifying the leading cause as witnessing patients' suffering (Jawed et al., 2021). Nurses working with undocumented migrant children and families also reported moral distress stemming from disparities in care and legal constraints that prevented them from offering comprehensive support. These nurses often witnessed the detrimental impact of such limitations on their patients’ health and well-being, leaving them feeling powerless to advocate for systemic change (Stephen & Zoucha, 2021).

The sources of moral distress are multifaceted. Legal restrictions that exclude undocumented migrants from healthcare coverage and benefits create substantial barriers to equitable care (Stephen & Zoucha, 2021). Institutional policies that prioritise financial considerations over ethical obligations, such as those promoting medical repatriation, further exacerbate the ethical challenges faced by providers (Kuczewski, 2019). Additionally, resource limitations—including insufficient interpreters, inadequate cultural competency training, and a lack of social support services—intensify moral distress. Healthcare providers may feel ill-equipped to offer culturally sensitive care or address the complex needs of undocumented migrants, leading to feelings of inadequacy, frustration, and helplessness (Richard & Brisbois, 2019).

Building on these concerns, insights from Lipsky’s street-level bureaucracy theory further illuminate the mechanisms through which moral distress develops in this context. As this theory predicts (Cooper et al., 2015; Lipsky, 2010), ambiguous entitlement policies and the persistent tension between professional ethics and institutional rules effectively position healthcare providers as street-level bureaucrats—frontline actors whose discretionary decisions determine how policy is enacted in practice. In settings where eligibility criteria are unclear or restrictive, providers become involuntary brokers between policy and undocumented migrants, and their judgements shape patients’ real-world access to care. This dynamic forces healthcare workers to devise creative strategies to address the often complex health and social needs of undocumented migrants while simultaneously navigating institutional constraints. Yet discretion operates within limits: even when providers wish to offer equitable, comprehensive care to all patients, resource scarcity and legal constraints may compel them to make difficult micro-level choices about whom they can help, prioritising the most vulnerable or the most straightforward cases. The moral weight of these discretionary decisions—decisions that are structurally imposed yet ethically consequential—contributes substantially to providers’ moral distress, as they bear responsibility for outcomes shaped by forces beyond their control.

Addressing moral distress and moral injury requires a comprehensive and nuanced approach. According to Griffin et al., interventions should recognise the psychological, social, and spiritual dimensions of these experiences, ensuring that healthcare providers receive the necessary ethical, emotional, and institutional support to navigate such challenges. This includes fostering organisational cultures that prioritise ethical decision-making, providing access to counselling and peer support, and implementing systemic changes to reduce the structural inequities that drive moral distress (Griffin et al., 2019). By addressing these underlying issues, healthcare providers can be empowered to continue their work effectively while mitigating the long-term psychological and emotional toll of their professional roles. Conversely, failing to address these challenges may intensify distress and lead to professional burnout. In our conceptual model (supplementary material), this burnout is represented in the outer ring, illustrating how unresolved strain can further compromise both provider’s well-being and the quality of care delivered.

#### Professional burnout: a long-term consequence of moral distress and systemic barriers

Bridgeman et al. conceptualised professional burnout as a syndrome resulting from prolonged occupational stress, characterised by three core dimensions: emotional exhaustion, depersonalisation, and reduced personal accomplishment. Emotional exhaustion involves feeling drained and fatigued by work, often resulting in apathy, reduced empathy, and diminished engagement. Depersonalisation refers to a detached or cynical attitude toward work, reducing human interactions to impersonal, technical tasks. Reduced personal accomplishment reflects a sense of inefficacy, where individuals feel their efforts are inadequate, undermining both job satisfaction and professional identity (Bridgeman et al., 2018).

The ethical challenges and systemic barriers identified in this review—such as legal restrictions, resource constraints, and institutional pressures—exacerbate these dimensions of burnout. This aligns with Humikowski's description of professional burnout as “human fracking” (Humikowski, 2018), a metaphor that aptly illustrates how external pressures—such as administrative burdens, excessive workloads, and inadequate systemic support—fracture resilience and deplete healthcare providers' emotional and professional reserves.

Building on the concept of burnout as a response to systemic pressures, healthcare providers working with migrants and refugees face heightened risks of burnout and secondary traumatic stress. The emotional toll of repeatedly hearing trauma narratives further amplifies these risks. Research indicates that approximately 30% of providers in such settings experience severe burnout (Roberts et al., 2021). These findings align with themes that emerged from our review, particularly those related to the moral and psychological experiences of healthcare providers.

Structural vulnerability theory offers further insight into the systemic roots of burnout among healthcare providers caring for undocumented migrants. Bourgois et al. conceptualise structural vulnerability as an increased risk of negative health outcomes arising from one’s position within overlapping social, political, economic, and institutional hierarchies that constrain agency, limit access to care, and shape exposure to harm. They operationalise this concept through a Structural Vulnerability Assessment Tool designed to support clinicians in identifying upstream determinants of harm and cultivating structural competency. Drawing on broader critical social science, they underscore that clinicians increasingly confront forms of suffering produced by political and economic forces outside the clinic, highlighting the need for an orientation that recognises these structural conditions (Bourgois et al., 2017).

This understanding of structural vulnerability helps clarify patterns observed in our review, particularly why providers experienced their work as not only challenging but morally injurious. They were situated within systems that rendered undocumented migrants structurally vulnerable yet were not given the authority, resources, or institutional pathways to address the upstream determinants driving this vulnerability. This mismatch between responsibility and capacity for action intensified the moral burden providers described, making their distress a reflection of systemic failures rather than individual shortcomings.

Scholars have also emphasised that structural vulnerability extends to healthcare providers themselves. Morera et al. highlight how societal expectations of clinicians as exceptionally resilient or “superhuman” obscure their own vulnerability and create stigma around acknowledging distress or seeking support (Morera et al., 2024), while Catherine Smith demonstrates that vulnerability among healthcare workers is unevenly distributed and shaped by systemic conditions such as limited resources and institutional neglect (Smith, 2020). These insights parallel our findings: providers caring for undocumented migrants often confronted complex needs and ethically fraught decisions without adequate institutional backing. Their vulnerability was therefore structurally produced—emerging from prolonged exposure to patients’ structural harms, responsibility without corresponding institutional authority or support, and cultural narratives that obscured their own need for care. Recognising this structural dimension of provider vulnerability is essential for designing interventions that move beyond individual coping strategies and address the systemic conditions that generate burnout and moral distress.

Accordingly, we recommend that institutions move beyond individualised resilience-building and prioritise structural responses to moral distress. This includes providing access to ethics consultation, and embedding regular supervision, reflective practice, and structured debriefing mechanisms into routine clinical work. These measures directly address the situations described in the primary studies, where providers faced sustained exposure to ethically fraught decisions with limited institutional backing and few opportunities for shared deliberation or support. By instituting such systemic interventions, healthcare organisations can alleviate the structurally produced vulnerability experienced by providers and create conditions that enable ethically sustainable care for undocumented patients.

#### Challenges to nurses’ autonomy in undocumented migrant healthcare and strategies for enhancement

The experiences of burnout and moral distress cannot be fully understood without examining the dynamics among healthcare providers themselves. Inter-provider relationships emerged as a significant yet underexplored theme that shapes the ethical landscape of healthcare delivery for undocumented migrants. These relationships are influenced by varying professional roles, decision-making hierarchies, and differing perceptions of undocumented migrants' health entitlements. These relational dynamics often intersect with systemic barriers, leading to conflicts, disparities in autonomy, and inconsistencies in care delivery.

Our systematic review highlights that some nurses reported limitations to their professional autonomy despite their expanded roles. These limitations were particularly evident in decisions related to hospital referrals for undocumented migrants, prescribing medications, and interactions with immigration authorities. Such challenges raise critical questions about the boundaries of professional autonomy and its implications for ethical care.

Professional nurse autonomy is a multidimensional concept that encompasses the ability and confidence of nurses to make responsible, discretionary decisions independently and collaboratively within the scope of their professional practice. It is rooted in the belief in patient-centred care, prioritising advocacy and well-being (Wade, 1999). In the context of caring for undocumented migrants, professional autonomy becomes especially critical, as nurses are often on the frontline, addressing the unique and complex needs of this vulnerable population.

Pursio et al identified four key themes that shape professional autonomy in nursing: shared leadership, professional skills, inter- and intra-professional collaboration, and the establishment of a healthy work environment. Shared leadership fosters participative decision-making, empowering nurses to play an active role in shaping care strategies. The development of professional skills enhances nurses' clinical competence and equips them to make informed decisions. Collaboration among healthcare providers, both within and across professions, strengthens care delivery and creates a sense of mutual support. Finally, a healthy work environment that promotes independence and decision-making is essential for advancing nurses’ autonomy (Pursio et al., 2021).

In our perspective, these strategies not only enhance the professional autonomy of nurses but also have profound implications for the provision of healthcare to undocumented migrants. Shared leadership ensures that nurses actively contribute to care strategies for undocumented patients, fostering more inclusive and culturally sensitive approaches. Enhanced professional skills enable nurses to address the complex health needs of undocumented migrants with greater confidence and efficacy. Strong inter- and intra-professional collaboration helps healthcare teams navigate the ethical and logistical challenges of providing care, ensuring coordinated and compassionate service. Lastly, a supportive work environment empowers nurses to advocate for undocumented migrants while maintaining their professional integrity.

Enhancing nurses’ participation in decision-making and fostering appreciation among physicians for their unique contributions are both ethical imperatives and practical necessities. Achieving these goals requires a cultural shift within healthcare institutions to reduce hierarchical barriers and promote mutual respect. Such efforts can improve the resolution of moral conflicts, lead to better patient outcomes, and enhance job satisfaction for nurses (Poškutė et al., 2022).

#### Healthcare providers' perceptions of undocumented migrants' health entitlements: the case for a rights-based approach

The dynamics within healthcare teams are not the sole determinant of care delivery. Healthcare providers’ perceptions of undocumented migrants and their entitlements significantly influence not only their approaches to care but also their engagement in advocacy efforts. These perceptions arise from a complex interplay of personal values, systemic biases, and broader sociopolitical narratives. While some providers view health as a fundamental right that should be upheld regardless of immigration status, others consider access to care contingent on legal and financial eligibility.

Protecting the human right to health for undocumented migrants is a legal and ethical necessity, as their exclusion from healthcare violates international commitments to ensuring equal access to health services. However, many countries are increasingly restricting healthcare for undocumented migrants under nationalist policies. This shift represents a retreat from the principle of universal human rights toward a system where healthcare is tied to citizenship, eroding the fundamental idea that healthcare is a right for all, regardless of legal status.

In the supplementary material, we demonstrate that healthcare entitlements for undocumented migrants vary widely across national contexts. Even in countries with established welfare or national health systems, access to care for undocumented migrants is often legally restricted and administratively fragmented. Across several settings, we also identify persistent gaps between human rights commitments and their practical implementation. For instance, in the United States, healthcare coverage is not universal even for citizens. In Denmark, while universal healthcare is guaranteed for citizens and permanent residents, it excludes undocumented migrants. In England, access to secondary care follows a residence-based system that renders most undocumented migrants ineligible. In the Netherlands, mandatory health insurance is restricted to those with legal residency status, effectively excluding undocumented individuals. In Norway—despite its strong welfare system—only minimal entitlements are extended to undocumented migrants, reflecting a Nordic welfare model that prioritises legal residency and economic stability over inclusive access.

These examples underscore the tension between citizenship-based entitlements and the universal human right to health, and illustrate the diverse contexts in which healthcare providers operate when delivering care to undocumented migrants. To address this tension, Ooms et al. underscore the need to reinforce the notion of collective human responsibility, emphasising that healthcare should be upheld as a universal right rather than a privilege restricted by legal status (Ooms et al., 2019). In the same vein, Onarheim et al. emphasise that achieving universal healthcare coverage for undocumented migrants requires a rights-based approach that prioritises affordability, accessibility, and inclusion for all. Without such an approach, healthcare systems fail to meet their ethical and legal obligations, perpetuating inequalities and preventing undocumented migrants from accessing essential and preventive care, ultimately compromising public health outcomes (Onarheim et al., 2018).

We find that recognising this rights-based approach is crucial for healthcare providers caring for undocumented migrants, as it ensures that ethical and professional obligations toward this cohort of patients are met. This realisation requires understanding that healthcare is not merely a service but a fundamental right that must be upheld regardless of a patient's legal status. It also necessitates awareness of the structural and policy-level barriers that undocumented migrants face, as well as the willingness to advocate for policies that promote equitable access to care. Moreover, embracing a rights-based approach means that providers must actively challenge discriminatory practices, adopt inclusive care models, and engage in ethical reflection to balance individual patient needs with systemic constraints. By doing so, healthcare providers contribute to a more just and effective healthcare system that aligns with the universalistic ethical notions, such as the principle of health equity.

Central to this shift is strengthening healthcare providers’ understanding of the legal and ethical foundations of undocumented migrants’ health entitlements and the scope of care to which they are entitled. Integrating this knowledge into professional education and institutional training, and reinforcing it through clear organisational protocols, equips providers with the clarity needed to interpret entitlements correctly and to act consistently with a rights-based approach. Such clarity not only reduces the moral uncertainty that often arises in encounters with undocumented patients but also supports providers in challenging exclusionary practices and ensuring that care delivery aligns with established human rights obligations.

Nonetheless, our systematic review reveals that healthcare providers varied in their awareness of undocumented migrants’ health entitlements, with perceptions often polarised. In several contexts, lack of awareness was manifested as uncertainty about professional obligations—particularly regarding confidentiality and whether undocumented migrants should be reported to authorities. This uncertainty at times resulted in administrative invisibility, where eligible undocumented patients were overlooked despite having legal entitlements to care. Moreover, negative perceptions of undocumented migrants often stemmed from misconceptions that portrayed them as burdens on receiving countries and healthcare systems rather than as contributors to the economy.

This polarisation is also evident in the broader literature. For instance, Scott et al. found that general practitioners had limited awareness of the health rights of refugees, asylum seekers, and undocumented migrants. A significant proportion were unfamiliar with the distinctions between these groups, highlighting a gap in foundational understanding. Additionally, there was widespread uncertainty about registration policies and the healthcare services to which migrants are entitled. Confusion also extended to legal responsibilities; some mistakenly believed they were required to report undocumented migrants to authorities, while others were uncertain about the correct course of action (Scott et al., 2019).

Serre-Delcor et al. found that a significant portion of healthcare and social care professionals lacked knowledge regarding migrants' health rights, with 61% admitting to having limited or no understanding of these entitlements. Despite working directly with newly arrived migrants, only 29% of professionals had received formal workplace training on the topic, highlighting a gap in professional development and preparedness (Serre-Delcor et al., 2021). Another cross-sectional study found that healthcare providers’ attitudes toward healthcare access varied; broad or full access was more commonly supported by foreign-born respondents, clinicians, and those in primary care settings. However, 61.1% of those who favoured restricted access still endorsed health as a human right, highlighting a paradox between principles and practice (Ruiz-Casares et al., 2013).

While these findings highlight a critical gap in healthcare providers’ knowledge of migrants’ health entitlements, they also raise deeper questions about how perceptions of undocumented migrants influence care delivery. Beyond knowledge deficits, the way healthcare providers conceptualise undocumented migrants—whether as individuals entitled to care or as outsiders whose presence is contested—shapes both their attitudes and practices.

#### The othering of migrants: sociopolitical dynamics and their impact on healthcare providers’ attitudes and practices

In our review, we found that some healthcare providers perceive undocumented migrants as “others”, distinguishing them from the broader patient population in ways that reinforce social, legal, and moral boundaries. This process of othering is not merely a reflection of individual biases but is rooted in broader sociopolitical narratives that portray undocumented migrants as a burden on healthcare systems or as morally undeserving of care. Such framing can influence providers’ willingness to advocate for their undocumented patients, their emotional engagement in patient care, and their decisions regarding treatment access (Abdulrazeq et al., 2024).

Othering theory provides a valuable lens for understanding these dynamics. Rooted in social and critical theory (Roberts & Schiavenato, 2017), othering is a social process in which a dominant group defines and differentiates others based on perceived deviations from accepted social norms, reinforcing exclusion and marginalisation. It functions by identifying differences—often tied to race, ethnicity, or gender—resulting in the subordination of those deemed the Other. Othering can be both conscious and unconscious, contributing to negative patient experiences, reduced healthcare quality, and deepening inequities.

The othering of forced migrants (Grove & Zwi, 2006), including undocumented migrants, manifests through various mechanisms that reinforce their social exclusion. Refugees are often framed as a threat, depicted as an “invasion” or a “security risk”, which fuels fear and justifies restrictive policies. The “queue-jumping” and “uninvited guest” narratives portray asylum seekers as bypassing an orderly process, distinguishing “good” (offshore) from “bad” (onshore) refugees—creating divisions that further marginalise those seeking asylum through irregular means. A charity-based perspective strips refugees of agency, casting them as passive recipients rather than individuals with rights. The perceived strain on resources exaggerates their impact on healthcare and welfare, fostering hostility and restrictive measures. Finally, maintaining the other ensures physical and social distance through detention, dispersal strategies, and the criminalisation of protests, further alienating and marginalising forced migrants.

To challenge the othering discourse, healthcare providers must advocate for inclusive healthcare policies that ensure access to services for forced migrants, regardless of their legal status. They should reframe narratives to emphasise migrants' rights rather than portraying them as burdens. Additionally, healthcare providers must promote evidence-based policies, document the health impacts of exclusionary practices, and actively oppose restrictive measures such as detention and limited healthcare access. By fostering inclusivity and social justice, providers can counter harmful narratives and enhance health outcomes for both migrants and host communities (Abdulrazeq et al., 2024; Grove & Zwi, 2006).

Complementing these provider-level actions, we recommend that institutions implement strategies that confront these forms of othering within organisational culture. This includes creating protected spaces for ethical reflection and dialogue, supporting anti-discrimination and cultural humility training, and involving migrants and civil society actors in the co-design of services. Such measures directly respond to the forms of marginalisation that emerged across the studies in our review. By embedding these structural approaches into routine practice, healthcare institutions can counter exclusionary narratives and foster a more equitable and inclusive environment for undocumented migrants.

#### Emotional detachment in healthcare: a coping mechanism with ethical consequences

While advocating for inclusive policies and opposing exclusionary measures are essential steps toward addressing the broader systemic injustices faced by undocumented migrants, healthcare providers must also contend with the personal toll of working under these challenging conditions. The emotional burden of witnessing preventable suffering, coupled with institutional and legal constraints that hinder comprehensive care, often leads providers to adopt various coping mechanisms. One such mechanism that emerged from our findings is emotional detachment—a strategy some healthcare providers use to shield themselves from the psychological distress of their work. Although emotional detachment may serve as a protective measure, its implications extend beyond the individual provider, raising ethical concerns and influencing patient-provider relationships and the overall quality of care.

Emotional detachment, characterised by depersonalisation, significantly compromises the quality of patient care by increasing the likelihood of patient safety incidents, diminished professionalism, and patient dissatisfaction. Healthcare providers experiencing such detachment may become indifferent toward those they serve, leading to reduced empathy, impaired communication, and suboptimal clinical decision-making (Hodkinson et al., 2022). As depicted in the conceptual model (supplementary material), negative perceptions and arbitrary attitudes—further intensified by detachment—create barriers between providers and undocumented migrants (represented as a brick wall). These barriers restrict advocacy, erode trust, and ultimately hinder access to healthcare for undocumented migrants.

Although emotional detachment may serve as a short-term coping mechanism—a self-shielding strategy—complete disengagement is neither a sustainable nor an ethically sound in managing distress within healthcare. A more balanced approach, characterised by regulated emotional engagement, enables providers to maintain compassion while preserving professional boundaries. As conceptualised by Tan et al., clinical empathy extends beyond a purely cognitive exercise; it involves an interplay of imaginative, affective, and cognitive elements that foster meaningful connections between providers and patients. This connection is strengthened through genuine concern and effective communication, ensuring that empathy is both experienced and expressed in ways that build trust and enhance therapeutic relationships (Tan et al., 2021).

In our perspective, adopting regulated emotional engagement can help healthcare providers navigate emotional challenges while preserving their well-being and the ethical integrity of patient care. We believe that this approach fosters a more supportive healthcare environment for undocumented migrants, where they are treated with respect and a sense of human connection rather than as mere cases to be managed.

#### Ethical dilemmas in healthcare for undocumented migrants: balancing duty and legal constraints

Throughout this discussion, we have explored moral distress and the ethical tensions arising from inter-provider dynamics and systemic barriers that shape healthcare providers' perceptions, attitudes, and practices. However, beyond these challenges, our findings highlight additional ethical dilemmas that directly impact healthcare delivery for undocumented migrants, including the conflicts between beneficence and non-maleficence, the tension between professional duty and legal compliance, and the moral dilemma of deservingness.

In caring for undocumented migrants, healthcare providers must balance their ethical obligation to maximise benefits while minimising harm, ensuring that their interventions do not inadvertently cause unintended risks due to limited resources or non-traditional care settings. The principle of informed consent and transparency is essential, as providers must clearly communicate potential risks while acknowledging the precarious legal and social status of their patients.

Consequently, under certain circumstances, explicit documentation of immigration status should be avoided when the risks outweigh the benefits. Instead, healthcare providers can use indirect phrasing—such as noting “immigration-related stressors”—to ensure continuity of care while safeguarding patient confidentiality. Additionally, informing patients about their privacy rights under healthcare regulations can help alleviate fears, foster trust, and encourage them to seek necessary medical care without hesitation (Kim et al., 2019). These precautions are crucial for preventing unintended harm while maintaining ethical and professional integrity.

Another factor in maximising benefits is building and maintaining trust, especially for healthcare providers working in detention centres or with undocumented migrants facing deportation fears. To achieve this (D'Alonzo & Greene, 2020), providers can adopt several strategies. First, they can conceptualise trust as a dynamic continuum, recognising that it develops over time through consistent, respectful interactions. Additionally, providers should acknowledge the stressors affecting undocumented migrants, including legal uncertainties and social isolation, and actively dispel harmful myths about healthcare access and immigration enforcement. Further, building bridges with community organisations, engaging in culturally sensitive communication, and establishing a positive presence in immigrant communities through outreach initiatives can foster long-term trust. We find these strategies not only encourage timely care-seeking but also uphold the ethical principles of beneficence and non-maleficence, ultimately improving health outcomes.

In addition to direct medical care (Abdulrazeq et al., 2024), providers can adopt various strategies to improve accessibility despite systemic constraints. Some leverage their professional and personal networks to connect undocumented migrants with specialists or institutions willing to offer care. Others adopt creative resource allocation approaches, such as navigating existing policies to secure funding or access to essential treatments. Additionally, some engage in case-by-case advocacy, negotiating with hospital administrators or policymakers to facilitate care provision for patients in urgent need. We find these efforts, though ethically complex, represent pragmatic approaches to fulfilling the dual responsibility of beneficence and non-maleficence while operating within restrictive healthcare systems.

#### The moral dilemma of deservingness: ethical and policy debates in healthcare access for undocumented migrants

Another critical ethical challenge in healthcare delivery for undocumented migrants is the moral dilemma of deservingness. Healthcare providers must navigate the tension between addressing undocumented migrants' health needs and responding to societal judgements about who is entitled to care—often in resource-limited settings where difficult decisions must be made. Examining how notions of deservingness influence healthcare decision-making is essential for understanding the ethical complexities that providers encounter in their efforts to deliver equitable care.

Our review indicates that healthcare providers conceptualise healthcare deservingness from multiple perspectives, including the moral worth of migrants, utilitarian principles, and the values of equality, equity, and humanitarianism. While some providers demonstrated greater solidarity with undocumented migrants, others prioritised the healthcare system's integrity. This dual allegiance is also evident in the literature. For instance, Vanthuyne et al. found that healthcare providers held divergent views on the healthcare deservingness of individuals with precarious immigration status. Some providers advocated for universal access, framing healthcare as a fundamental human right grounded in principles of humanitarianism, social justice, and public health. Conversely, others opposed such access, citing concerns related to system abuse, resource constraints, and the legality of residency status. Supporters of universal access emphasised the ethical obligation to care for vulnerable populations, particularly children and pregnant women, whereas opponents perceived certain migrants as “fraudsters” who exploit healthcare services without contributing to the system through taxation (Vanthuyne et al., 2013).

Perceptions of deservingness influence patient outcomes by shaping access to and the quality of healthcare provided to migrants, often reinforcing systemic inequities. Levesque et al. Patient-Centred Access to Healthcare Framework is helpful here (Levesque et al., 2013). It conceptualises access as the opportunity to identify healthcare needs, to seek care, to reach services, to obtain or use them, and ultimately to have those needs fulfilled. The framework outlines five dimensions of accessibility (approachability, acceptability, availability and accommodation, affordability, and appropriateness) and five corresponding population abilities (to perceive, seek, reach, pay, and engage). When interpreted alongside our findings, this framework clarifies how concrete perceptions of deservingness become embedded in each of these stages and shape pathways to care.

In several studies, some healthcare providers portrayed undocumented migrants as morally inferior, fraudulent, or as an unfair burden on the healthcare system. Such perceptions reduce the approachability and acceptability of services and undermine migrants’ ability to perceive themselves as legitimate users of care or to seek help without shame or hesitation. By contrast, other providers explicitly grounded their practice in equality, equity, and humanitarianism—affirming undocumented migrants’ entitlement to care. These perceptions appear to strengthen migrants’ ability to seek, reach, and engage with services by validating their presence in the healthcare system and reducing fear-driven barriers. Additionally, practices such as requesting documents that providers knew migrants could not provide, rendering them administratively invisible, or selectively prioritising patients perceived as more deserving illustrate how deservingness judgements can translate into constrained availability and accommodation and weakened abilities to reach and engage with care in practice.

This moral assessment, often based on legal status, nationality, race, or socioeconomic background, can result in preventable morbidity and mortality, as seen in cases where life-saving interventions are withheld from certain groups while granted to others. Thus, deservingness does not merely shape attitudes; it actively structures the conditions under which access to care is enabled or denied. Addressing these disparities requires integrating structural competency and cultural humility into medical education, advocating for policies that decouple healthcare from immigration enforcement, and fostering systemic reforms that prioritise equitable access to healthcare regardless of migration status (Holmes et al., 2021).

According to Lo and Nguyen, healthcare providers can resist the racialization of medical deservingness by blending neoliberal norms (such as self-sufficiency and responsibility) with social justice values (including universal healthcare rights and communal solidarity). They can challenge the assumption that citizenship status determines healthcare access by reassuring patients that their medical needs are valid regardless of documentation. By emphasising co-ethnic bonds and using culturally sensitive communication, healthcare providers can help undocumented migrants overcome internalised undeservingness and encourage them to seek necessary care (Lo & Nguyen, 2021).

Last but not least, when assessing deservingness through a utilitarian perspective, restricting access to healthcare and welfare benefits does not inherently lead to cost savings. Instead, exclusionary policies may impose greater burdens on public health systems while exacerbating health disparities. From a cost-effectiveness standpoint (Juárez et al., 2019), a more sustainable approach involves implementing preventive healthcare measures and inclusive welfare policies that facilitate early medical intervention. This strategy not only reduces long-term healthcare costs associated with untreated conditions but also mitigates the adverse consequences of policies that deepen societal inequities and public health challenges.

For example, a study conducted in Germany analysing the effects of restrictive healthcare policies on asylum seekers and refugees between 1994 and 2013 found that per capita healthcare expenditures for asylum seekers and refugees with restricted access were, on average, 40% higher than those with full healthcare entitlements. This disparity underscores the financial burden resulting from delayed or restricted access to medical care. The cumulative excess cost attributed to restricted access was estimated at approximately 1.56 billion Euros, highlighting that such policies do not effectively reduce healthcare expenditures. Instead, they contribute to higher overall costs, primarily due to increased reliance on emergency and secondary healthcare services (Bozorgmehr & Razum, 2015).

These findings emphasise the urgent need to shift migration health debates from politically motivated economic arguments to evidence-based policy discussions. As Gottlieb et al. propose, future research should focus on generating robust evidence on the economic impacts of migrant health policies, incorporating migrants in routine health data collection, and conducting qualitative studies to reveal the hidden costs of exclusion. It should also critically analyse economic arguments in migration debates, investigating why certain narratives persist despite contradicting evidence. Additionally, research must assess the ethics and politics of economic evidence, ensuring that healthcare access is not reduced to economic utility. To maximise impact, studies should prioritise science communication, ensuring that findings effectively inform policymaking. Finally, interdisciplinary collaboration—across public health, economics, and political science, alongside migrant communities—is essential to debunk economic myths and advance evidence-based policymaking (Gottlieb et al., 2020).

However, it is important to acknowledge that utilitarian reasoning also carries the risk of reinforcing a conditional model of healthcare access, where patients’ worthiness of care is evaluated based on their perceived economic and social contributions rather than their inherent human dignity. While utilitarian arguments can serve as strategic tools for expanding healthcare access, they must be critically examined alongside justice-based frameworks to safeguard healthcare as a fundamental right, rather than a privilege contingent upon utility.

### Implications and recommendations

#### Implications

Our findings underscore that the ethical challenges faced by healthcare providers go beyond individual dilemmas, reflecting broader systemic, institutional, and policy-level constraints that shape healthcare provision for undocumented migrants and highlight the urgent need for structural reform. Addressing these challenges requires a shift from reliance on individual ethical resilience to institutional and policy-level interventions that align healthcare delivery with ethical principles of justice, equity, and human rights. Moreover, fostering an inclusive healthcare environment, reinforcing professional autonomy, and equipping providers with ethical guidance can mitigate moral distress and improve care outcomes for undocumented migrants. Ultimately, our review calls for greater interdisciplinary collaboration among policymakers, healthcare institutions, and researchers to advance ethically sound and pragmatic solutions for healthcare providers working in migration contexts.

#### Recommendations

To translate our findings into actionable steps, we propose targeted recommendations for key actors, including policymakers, healthcare institutions, healthcare providers, and researchers. Grounded in the themes identified in this review, these recommendations directly correspond to the ethical and practical challenges described by healthcare providers. Calls for rights-based and non-discriminatory entitlement frameworks respond to the structural barriers, restrictive policies, and administrative obstacles encountered by healthcare providers. Proposals for stronger institutional support—such as ethics protocols, supervision and debriefing structures, ethics committees, and access to psychological support—emerge from providers’ accounts of moral distress, burnout, and uncertainty about their legal and ethical responsibilities. Likewise, recommendations for training on migrants’ health rights, health equity, and anti-discrimination practices reflect the documented influence of providers’ perceptions and attitudes on undocumented migrants’ access to, and experience of, care. Together, these measures aim to address the ethical tensions identified in this review while fostering systemic changes that promote equitable and ethically sound healthcare for undocumented migrants. For a detailed list of recommendations, please refer to Table IV.

**Table IV.** Recommendations for key stakeholders in healthcare for undocumented migrants.

**Policy-makers: Strengthening legal and institutional frameworks**	
1- Adopt a rights-based approach to healthcare access	Recognise healthcare as a fundamental human right rather than a privilege tied to legal status. Align national healthcare policies with international human rights frameworks and public health commitments to ensure equitable access for undocumented migrants.
2- Establish legal safeguards to protect undocumented migrant patients' health data and uphold provider autonomy	Develop clear legal frameworks to safeguard the confidentiality of migrant patients while establishing ethically justified conditions for the disclosure of healthcare information to immigration authorities. Provide legal clarity on healthcare providers' professional obligations and rights, ensuring protection from legal repercussions while delivering ethical care to undocumented migrants.
3- Address institutional and structural barriers to care	Develop policy frameworks that eliminate administrative obstacles to healthcare access for undocumented migrants. Implement streamlined referral systems to facilitate access to specialised medical care, mental health services, and chronic disease management.
**Healthcare institutions: Implementing ethical and structural reforms**	
4- Develop institutional ethical protocols for treating undocumented migrants	Establish institutional policies that balance legal obligations, ethical responsibilities, and the healthcare entitlements of undocumented migrants. Provide structured ethical guidance and decision-making support to assist providers in navigating moral distress.
5- Implement comprehensive training on migrant healthcare ethics	Integrate ethics-focused education into medical and professional training curricula, emphasising moral dilemmas, health equity, migrants' health rights, and culturally sensitive care. Offer continuous professional development on navigating the ethical and legal complexities of treating undocumented migrant patients.
6- Enhance psychological and institutional support for healthcare providers	Establish structured peer-support groups and mental health counselling services to help providers manage moral distress and burnout. Implement multidisciplinary ethics committees to support providers in complex cases and alleviate the psychological burden of ethically challenging decisions.
7- Promote institutional advocacy and partnerships	Strengthen collaborations between healthcare institutions, legal aid organisations, and migrant support networks. Encourage institutional engagement in advocating for policies that protect the autonomy of healthcare providers and the rights of undocumented migrant patients.
**Healthcare providers: Ethical strategies to navigate moral and legal challenges**	
8- Adopt ethical workarounds while upholding professional integrity	Use ethically sound documentation practices that safeguard patient confidentiality (e.g., noting “immigration-related stressors” rather than explicitly documenting legal status). Navigate restrictive policies through case-by-case advocacy to ensure that undocumented migrant patients receive the necessary care within existing frameworks.
9- Strengthen trust and culturally competent care practices	Build trust through empathetic, transparent, and patient-centred communication, acknowledging the vulnerabilities of undocumented migrants and their concerns about deportation and exclusion. Develop culturally sensitive care models that account for sociopolitical, linguistic, emotional, legal, and economic factors, as well as the unique circumstances and unstable living conditions of undocumented migrant patients.
10- Engage in ethical reflection and collaborative decision-making	Regularly participate in case-based ethical deliberations to discuss complex care decisions and share best practices with colleagues. Seek institutional and peer support systems to mitigate moral distress and prevent emotional detachment characterised by depersonalisation.
11- Resist bias and the rationing of care based on perceived deservingness	Critically examine and challenge internalised biases that may influence care decisions. Commit to non-discriminatory treatment, ensuring that all patients—regardless of immigration status—receive dignified and equitable care.
12- Participate in policy and public advocacy for structural change	Actively participate with professional organisations, ethics committees, and policy advisory groups to advocate for ethical healthcare policies. Actively contribute to public discourse on migrant health rights by promoting evidence-based narratives and countering politically driven misinformation, in order to foster informed policy and public understanding.
**Researchers: Advancing ethical enquiry and evidence-based policy**	
13- Investigating the ethical challenges encountered by healthcare providers in the provision of care for undocumented migrants	Explore healthcare providers' experiences of moral distress, burnout, and ethical dilemmas in the context of providing care for undocumented migrants. Examine the coping mechanisms employed by healthcare providers and their long-term impact on the quality of care provided to undocumented migrants.
14- Documenting the consequences of restrictive healthcare policies targeting undocumented migrants	Generate empirical evidence on the public health, economic, and ethical implications of excluding undocumented migrants from healthcare services. Evaluate the comparative effectiveness of inclusive policies, using case studies from various healthcare systems.
15- Assessing the impact of structural and institutional reforms	Investigate the impact of institution-based ethics committees and frameworks in supporting healthcare providers with complex ethical decision-making, particularly in mitigating moral distress and enabling ethical care provision for undocumented migrants. Measure the outcomes of ethics-focused training programmes, particularly in strengthening moral resilience and enhancing ethical decision-making under legal and institutional constraints.
16- Translating research into policy and practice	Foster interdisciplinary collaboration among academics, policymakers, and healthcare institutions to translate research findings into practical reforms that promote ethical and equitable healthcare for undocumented migrants. Enhance science communication strategies to counter politically motivated misinformation and raise public awareness about its harmful effects—not only on the health of undocumented migrants but on public health as a whole.

#### Future research trajectories

Building on the themes generated in this review, we identify a set of future research trajectories aimed at extending the evidence base and deepening understanding of the ethical challenges and moral stressors experienced by healthcare providers. While the recommendations presented earlier outline actionable steps for institutions, policymakers, providers, and researchers, the trajectories introduced here reflect broader conceptual and methodological directions that can guide the next phase of scholarly enquiry.

The aim of proposing these trajectories is to clarify how the findings of this review can be further developed through focused qualitative investigations, theory-building, and context-sensitive analyses. They also highlight areas where current evidence remains limited due to constraints in primary studies—such as inconsistent reporting of professional roles and the absence of explicit links between healthcare policy and providers’ experiences. Advancing research in these directions will not only strengthen the conceptual foundations established in this review but also generate more nuanced, empirically grounded insights that can support equitable and ethically sound healthcare for undocumented migrants. For an overview of these proposed directions and suggested qualitative approaches, please refer to Table V.

**Table V.** Suggested research trajectories building on the present review.

Key area	Coverage in current review	Future research trajectories	Suggested qualitative approaches
**Analytical framework and data interpretation**	We synthesised 58 themes and subthemes into an overarching conceptual framework. Given the large number and diversity of the emergent themes, the data were interpreted and presented at a more conceptual and integrative level. This approach aligned with our objective to systematically consolidate a fragmented evidence base and produce a coherent synthesis of healthcare providers’ ethical challenges and moral stressors. However, the necessary breadth of interpretation limited the space for deeper exploration of individual themes, finer-grained distinctions, and more elaborated conceptual theorisation within the constraints of a single manuscript.	Conduct focused qualitative investigations that explore specific themes or clusters of themes—for example, the moral dilemma of deservingness or conflicts between legal obligations and professional ethics—in greater depth and contextual nuance. Develop richer conceptual insights by examining how healthcare providers make sense of their experiences, including how they interpret and navigate moral distress, conflicting duties, and restrictive policy environments in their everyday practice. Explore context-specific ethical challenges by studying selected clinical settings, institutional contexts, or provider groups to generate deeper understanding of the mechanisms that shape moral stress. Build theories grounded in empirical data to explain how providers negotiate ethical tensions and construct meaning in morally complex care environments.	**Interpretative phenomenological analysis** □ Well suited for in-depth, case-by-case examination of healthcare providers’ lived experiences of moral distress. □ Emphasises meaning-making and the double hermeneutic, allowing researchers to interpret how providers themselves understand and navigate ethically and emotionally challenging situations. **Grounded theory** □ Appropriate for developing explanatory models based on a selected subset of themes—for example, how providers navigate conflicting obligations when legal constraints contradict professional ethics, or how they manage moral tensions in resource-constrained settings. □ Supports the development of theories grounded in empirical data, such as those explaining the processes underlying ethical decision-making and the formation of moral judgements in practice. **Narrative analysis** □ Ideal for capturing how healthcare providers construct moral meaning through stories—for example, how they narrate their identities as caregivers versus agents of state policy, or how they describe their moral self-perception when working with undocumented migrants. □ Reveals how broader sociopolitical narratives shape individual moral framings and influence providers’ perceptions and attitudes.
**Policy contexts and their influence on providers’ ethical experiences**	We developed a country-by-country policy overview in the Supplementary Material to contextualise the health system settings in which healthcare providers operate and to clarify the scope of entitlements available to undocumented migrants. This contextualisation was intended to situate, rather than structure, our analytic framework. Our synthesis relied on published findings and selected quotations, without access to raw qualitative material or primary data collection. Only a small subset of primary studies directly linked specific healthcare policies or laws to providers’ moral distress; more often, providers referred broadly to “restrictive systems”, “administrative barriers”, or “legal constraints” without identifying particular policies or legal provisions. Policy regimes varied both across and within countries and were inconsistently described in the primary studies, making comparative policy inference methodologically challenging and posing risks of ecological fallacy. These considerations do not represent methodological limitations of our review, as the aim was not to conduct a formal comparative policy analysis but to synthesise ethical challenges and moral stressors across diverse qualitative studies.	Explore how distinct policy contexts result in or exacerbate moral distress among healthcare providers by examining specific entitlement frameworks, implementation gaps, and regulatory ambiguities. Investigate how varying degrees of access (e.g., emergency-only coverage, partial entitlements, or near-universal care) shape certain ethical challenges, such as inability to uphold professional standards and moral residue from denied or delayed care. Examine subnational differences (e.g., municipal vs. national entitlements, decentralised asylum systems, discretionary hospital policies, and regional reimbursement rules) to understand how everyday administrative interpretations of policies and laws influence providers’ moral distress. Study the impact of implementation gaps—where rights exist on paper but are not operationalized in practice—on providers’ emotional strain, ethical decision-making, and advocacy behaviours. Identify mechanisms of policy-driven moral distress, such as reporting obligations, fear of legal repercussions, or managerial and institutional pressures. Develop typologies of policy environments (e.g., restrictive-regulatory, inclusive-but-fragmented, welfare-based-but-citizenship-bound) and examine how these intersect with professional roles, institutional mandates, and organisational cultures to shape ethical experiences.	**Qualitative case studies** □ Well suited for examining how different policy contexts shape providers’ moral stress and ethical challenges. □ Enable comparison across nations, regions, or sectors (e.g., governmental vs. humanitarian) to explore structural determinants of moral distress. **Qualitative policy analysis combined with primary interviews (integrated design)** □ Merges documentary analysis of laws, regulations, and administrative procedures with in-depth interviews with providers who work under these frameworks. □ Particularly suitable for uncovering how policy texts are interpreted, enacted, resisted, or operationalized in clinical practice. **Mixed-methods designs** □ Qualitative components can identify mechanisms of policy-related moral stress, while quantitative components (e.g., surveys of burnout or moral distress indices) can assess their prevalence and variation across contexts. □ Especially valuable for linking contextual factors to measurable outcomes and triangulating patterns across policy environments. **Ethnographic approaches** □ Can illuminate how policy-driven administrative practices (e.g., reporting obligations, billing rules, reimbursement procedures) shape moral tensions in routine clinical encounters. □ Reveal the “everyday implementation” of policy and its ethical, relational, and emotional consequences for providers and patients.
**Professional roles and their influence on providers’ ethical experiences**	We documented the diversity of professional roles represented across the included studies and considered how these roles shaped the ethical challenges and moral stressors experienced by healthcare providers. Role-specific reflections were incorporated when such distinctions were explicitly articulated in the primary qualitative accounts (e.g., nurses’ constrained professional autonomy). Systematic comparison across professions was not feasible, as many primary studies did not clearly specify participants’ professional backgrounds or reported findings from mixed provider groups without disaggregation. This inconsistent reporting limited our ability to attribute particular ethical challenges to specific roles in a methodologically robust manner. Despite variation in professional backgrounds, many ethical challenges and moral stressors appeared across professions, indicating shared ethical pressures inherent to providing care for undocumented migrants.	Investigate how ethical challenges and moral stressors differ according to professional role, scope of practice, and degree of responsibility for clinical or administrative decision-making. Examine profession-specific mechanisms of moral distress (e.g., nurses’ proximity to patient suffering). Explore interprofessional dynamics to understand how hierarchical structures, team-based care, and role expectations shape ethical tensions and advocacy practices. Assess how training, codes of professional ethics, and organisational cultures influence providers’ ethical reasoning. Identify shared versus role-specific ethical burdens to inform tailored support interventions and ethics education.	**Focus group discussions** □ Useful for exploring collective interpretations of ethical challenges within specific professions. □ Capture areas of consensus, disagreement, and interprofessional tension in a dialogic environment. **Ethnographic approaches** □ Effective for observing role-specific practices, interprofessional interactions, and hierarchies in situ. □ Reveal how institutional routines, informal norms, and team dynamics shape moral stress in everyday clinical work. **Role-focused qualitative evidence synthesis** □ A synthesis targeting a specific profession can systematically map profession-specific ethical challenges across contexts. □ Generates role-specific conceptual insights and informs tailored training, support strategies, and policy design.

### Limitations

Several limitations should be considered when interpreting the findings of this systematic review. First, the review focused exclusively on peer-reviewed qualitative studies indexed in major bibliographic databases. While the search strategy was broad and the approach ensured methodological rigour, it may have excluded alternative sources of insight—such as unpublished reports, legal case documents, or narrative accounts shared via social media and activist platforms—which could offer valuable contextual depth or highlight emerging ethical concerns. Although such sources were beyond the scope of this review, future research may benefit from adopting more expansive search strategies and complementary methodologies to capture these perspectives and enrich the ethical analysis.

Second, while no geographical restrictions were applied during study selection, most of the included studies were conducted in the United States and Western Europe. This geographic concentration reflects the regions where qualitative research on this topic is most prevalent but may limit the transferability of findings to other contexts where legal, institutional, and sociocultural conditions differ significantly. The overrepresentation of certain settings may obscure region-specific ethical challenges and contextually grounded moral stressors encountered by healthcare providers in underrepresented areas such as Latin America, the Middle East, Southeast Asia, or sub-Saharan Africa. This geographic imbalance also reflects a broader structural pattern in global migration and health scholarship, wherein research produced in the Global North is disproportionately represented in peer-reviewed literature.

As a result, commonly used analytical frameworks and ethical debates are often shaped by Northern migration-health systems, policy regimes, and institutional cultures. This concentration risks overshadowing perspectives from the Global South, where healthcare systems, migration trajectories, migration-health policies, and provider–patient dynamics may differ substantially. Accordingly, the ethical challenges identified in this review may not fully capture the realities faced by healthcare providers in regions where qualitative research on healthcare delivery for undocumented migrants remains limited. Addressing this imbalance requires sustained empirical work in underrepresented settings to broaden the global evidence base and ensure that discussions on migrant health ethics draw on a more diverse and inclusive range of contexts and experiences.

Third, the review was limited to studies published in English, which may have led to the exclusion of relevant work in other languages, particularly from non-English-speaking countries. This language restriction may introduce a degree of cultural and contextual bias, potentially underrepresenting the diversity of healthcare providers' ethical experiences worldwide.

Fourth, while the use of qualitative synthesis methods enabled a rich thematic exploration, it necessarily involved interpretation and abstraction, which may have introduced subjectivity despite efforts to ensure analytic rigour through team-based review and structured coding. Future research could benefit from integrating mixed-method approaches or conducting comparative case studies across healthcare systems to triangulate findings and validate thematic interpretations.

Finally, although the review used CASP to appraise the methodological quality of included studies and adopted several strategies to support analytical rigour, no formal GRADE-CERQual or equivalent assessment of confidence in individual synthesised findings was conducted. CERQual assesses confidence in review findings by considering methodological limitations, coherence, adequacy of data, and relevance; however, its proper application requires a structured, finding-by-finding assessment rather than an assessment of the review as a whole or of broad themes. Given the large number of themes and subthemes generated in this review, and the interpretive and conceptually integrated nature of the synthesis, applying CERQual would have required a separate and detailed assessment process that was beyond the scope of the present review. Readers should therefore interpret the findings as conceptually integrated qualitative synthesis findings rather than as findings with formally assessed confidence. This consideration is particularly relevant for readers who wish to use specific findings to inform policy formulation or formal decision-making processes.

### Conclusion

This systematic review synthesised qualitative evidence on the ethical challenges and moral stressors healthcare providers face when delivering care to undocumented migrants. By analysing 37 qualitative studies, this review identified five interconnected concepts—experiences, perceptions, attitudes, practices and coping mechanisms, and ethical challenges—which together provide a comprehensive understanding of how providers navigate the ethical and professional complexities of caring for this marginalised population. Taken together, these concepts show that the review’s contribution is not simply the organisation of themes, but the clarification of how ethical pressure moves across levels of healthcare systems: from policy and institutional arrangements, through providers’ moral reasoning and coping strategies, to the care ultimately made available to undocumented migrants.

Healthcare providers’ experiences were shaped by moral distress, professional burnout, and the emotional burden of witnessing preventable suffering. These experiences influenced their perceptions, with some providers viewing healthcare as a universal right, while others framed care as a privilege tied to legal status. Such perceptions informed their attitudes, which ranged from compassionate and patient-centred to arbitrary and inconsistent, shaped by systemic constraints and individual biases. In response, healthcare providers adopted varied practices and coping mechanisms, including advocacy strategies, maintaining professional neutrality, and, in some cases, emotional detachment to manage the ethical weight of their work. At the core of these dynamics were ethical challenges, particularly the tension between beneficence and non-maleficence, conflicts between professional duty and legal compliance, and the moral dilemma of deservingness, all of which shaped decision-making in healthcare delivery for undocumented migrants.

The findings underscore that healthcare providers do not merely function within existing constraints but actively negotiate, resist, and adapt to systemic inequities. They employ advocacy strategies, workarounds, and coping mechanisms to reconcile their ethical obligations with institutional barriers. Seen through the lens of ethical burden-shifting, these responses are not only individual coping strategies; they also reveal how unresolved policy and institutional tensions become concentrated in everyday clinical encounters. However, these strategies do not fully resolve the inherent tensions in healthcare provision for undocumented migrants. When providers are left to manage these tensions case by case, the moral burden of exclusionary or ambiguous systems remains insufficiently addressed.

By shedding light on these challenges, this review contributes to the discourse on migrant health ethics and underscores the need for ethical guidelines, policy interventions, and institutional support systems to mitigate moral distress, enhance provider well-being, and ensure equitable healthcare access. Specifically, the findings highlight the need for clearer guidance on healthcare providers’ obligations toward undocumented patients and the scope of care to which they are entitled, as well as policy reforms that expand entitlements beyond emergency-only coverage and reduce administrative barriers to access. They also underscore the importance of institutional supports such as ethics consultation pathways, protected opportunities for reflective practice, and psychosocial resources for staff working under sustained moral strain.

Future research should explore context-specific interventions that strengthen ethical resilience among healthcare providers while advocating for systemic reforms that align healthcare delivery with principles of justice, equity, and human rights. Rather than treating moral distress only as an individual professional experience, future studies should examine how policy contexts, institutional rules, and professional roles shape when, where, and onto whom ethical burdens are shifted. This includes assessing how entitlement reforms, ethics-support infrastructures, and ethics-focused training programmes influence provider well-being and ethical performance, as well as undocumented migrants’ access to and experience of care.

## Supplementary Material

Healthcare policy contexts.docx Healthcare policy contexts.docx

PRISMA checklist.docx PRISMA checklist.docx

Conceptual Model.docx Conceptual Model.docx

Quality assessment of included studies.docx Quality assessment of included studies.docx

## Data Availability

All papers included in this systematic review are publicly available—most through open-access sources, while others may require institutional access depending on the institute's agreements with the respective journals. All other associated data will be made available upon reasonable request to the corresponding author.

## Endnotes

^1^
This term refers to residing in a country without a recognised legal authorisation, including entering without authorisation, overstaying a valid permit, breaching conditions of stay (e.g., working without authorisation), or being born to parents who are already in an irregular situation. The term is used to denote an administrative and contingent legal status, rather than to criminalise individuals, and is preferred over “illegal” in migration health and ethics literature.

^2^
Our analytic focus remained on healthcare providers’ accounts of ethical challenges and moral stressors across settings. Because this review relied on published findings without access to raw qualitative material, and only a subset of primary studies explicitly linked healthcare policy contexts to the ethical challenges faced by healthcare providers, imposing a policy-driven comparative frame would risk over-interpretation and ecological fallacy. Comparative inference also requires consistent reporting on entitlement scope, implementation, and enforcement—details that were absent or unevenly documented across the included studies. Given that entitlement regimes vary over time and across sub-national jurisdictions, the policy overview is used to situate, not structure, our findings: it provides contextual grounding for interpretation and transferability, while the themes that emerged remain anchored in the primary qualitative accounts.

## References

[cit0001] Abdulrazeq, F. , März, J. , Biller-Andorno, N. , & Gastmans, C. (2024). Healthcare providers' advocacy approaches and ethical challenges in delivering healthcare to undocumented migrants: A scoping review. *Medicine, Health Care and Philosophy* , *27* (4), 579–606. 10.1007/s11019-024-10225-8 39370496 PMC11519158

[cit0002] Abubakar, I. , Aldridge, R. W. , Devakumar, D. , Orcutt, M. , Burns, R. , Barreto, M. L. , Dhavan, P. , Fouad, F. M. , Groce, N. , Guo, Y. , & Hargreaves, S. (2018). The UCL–Lancet commission on migration and health: the health of a world on the move. *The Lancet* , *392* (10164), 2606–2654. 10.1016/S0140-6736(18)32114-7 PMC7612863 30528486

[cit0003] Aljadeeah, S. , Payedimarri, A. B. , Kielmann, K. , Michielsen, J. , Wirtz, V. J. , & Ravinetto, R. (2024). Access to medicines among asylum seekers, refugees and undocumented migrants across the migratory cycle in Europe: A scoping review. *BMJ Global Health* , *9* (10), e015790. 10.1136/bmjgh-2024-015790 PMC11481121 39414330

[cit0004] Armin, J. S. (2019). Administrative (in) visibility of patient structural vulnerability and the hierarchy of moral distress among health care staff. *Medical Anthropology Quarterly* , *33* (2), 191–206. 10.1111/maq.12500 30667109

[cit0005] Berlinger, N. (2019). Is it ethical to bend the rules for undocumented and other immigrant patients? *AMA Journal of Ethics* , *21* (1), 100–105. 10.1001/amajethics.2019.100 30672426

[cit0006] Bianchi, A. , Oths, K. S. , & White, K. (2019). Are the undocumented deserving? Health workers' views of immigrants in Alabama. *Journal of health care for the poor and underserved* , *30* (2), 820–840. 10.1353/hpu.2019.0058 31130553 PMC10409594

[cit0007] Biswas, D. , Kristiansen, M. , Krasnik, A. , & Norredam, M. (2011). Access to healthcare and alternative health-seeking strategies among undocumented migrants in Denmark. BMC Public Health, *11* , 1–11. 10.1186/1471-2458-11-560 21752296 PMC3163547

[cit0008] Bourgois, P. , Holmes, S. M. , Sue, K. , & Quesada, J. (2017). Structural vulnerability: operationalizing the concept to address health disparities in clinical care. *Academic Medicine* , *92* (3), 299–307. 10.1097/ACM.0000000000001294 27415443 PMC5233668

[cit0009] Bozorgmehr, K. , & Razum, O. (2015). Effect of restricting access to health care on health expenditures among asylum-seekers and refugees: A quasi-experimental study in Germany, 1994–2013. *PLoS One* , *10* (7), e0131483. 10.1371/journal.pone.0131483 26201017 PMC4511805

[cit0010] Brenner, J. M. , Blutinger, E. , Ricke, B. , Vearrier, L. , Kluesner, N. H. , & Moskop, J. C. (2021). Ethical issues in the access to emergency care for undocumented immigrants. *JACEP Open* , *2* (3), e12461. 10.1002/emp2.12461 34095898 PMC8164497

[cit0011] Bridgeman, P. J. , Bridgeman, M. B. , & Barone, J. (2018). Burnout syndrome among healthcare professionals. *The Bulletin of the American Society of Hospital Pharmacists* , *75* (3), 147–152. 10.2146/ajhp170460 29183877

[cit0012] Castañeda, H. (2011). Medical humanitarianism and physicians’ organized efforts to provide aid to unauthorized migrants in Germany. *Human organization* , *70* (1), 1–10. 10.17730/humo.70.1.a16566172p238244

[cit0013] Cervantes, L. , Richardson, S. , Raghavan, R. , Hou, N. , Hasnain-Wynia, R. , Wynia, M. K. , Kleiner, C. , Chonchol, M. , & Tong, A. (2018). Clinicians' perspectives on providing emergency-only hemodialysis to undocumented immigrants: A qualitative study. *Annals of internal medicine* , *169* (2), 78–86. 10.7326/M18-0400 29800062

[cit0014] Cooper, M. J. , Sornalingam, S. , & O’Donnell, C. (2015). Street-level bureaucracy: an underused theoretical model for general practice? *The British Journal of General Practice* , *65* (636), 376–377. 10.3399/bjgp15X685921 26120132 PMC4484940

[cit0015] D'Alonzo, K. T. , & Greene, L. (2020). Strategies to establish and maintain trust when working in immigrant communities. *Public Health Nursing* , *37* (5), 764–768. 10.1111/phn.12764 32638421 PMC7484021

[cit0016] Dauvrin, M. , Lorant, V. , Sandhu, S. , Devillé, W. , Dia, H. , Dias, S. , Gaddini, A. , Ioannidis, E. , Jensen, N. K. , Kluge, U. , & Mertaniemi, R. (2012). Health care for irregular migrants: pragmatism across Europe. A qualitative study. *BMC research notes* , *5* , 1–9. 10.1186/1756-0500-5-99 22340424 PMC3315408

[cit0017] De Casterlé, B. D. , Gastmans, C. , Bryon, E. , & Denier, Y. (2012). QUAGOL: A guide for qualitative data analysis. *International journal of nursing studies* , *49* (3), 360–371. 10.1016/j.ijnurstu.2011.09.012 21996649

[cit0018] Dierckx de Casterlé, B. , De Vliegher, K. , Gastmans, C. , & Mertens, E. (2021). Complex qualitative data analysis: lessons learned from the experiences with the qualitative analysis guide of leuven. *Qualitative Health Research* , *31* (6), 1083–1093. 10.1177/1049732320966981 33135554

[cit0019] Dos Santos, S. L. S. (2015). Undeserving mothers? Shifting rationalities in the maternal healthcare of undocumented Nicaraguan migrants in Costa Rica. *Anthropology & Medicine* , *22* (2), 191–201. 10.1080/13648470.2015.1004503 25639299

[cit0020] Doshi, M. , Lopez, W. D. , Mesa, H. , Bryce, R. , Rabinowitz, E. , Rion, R. , & Fleming, P. J. (2020). Barriers & facilitators to healthcare and social services among undocumented latino (a)/Latinx immigrant clients: perspectives from frontline service providers in southeast michigan. *PLoS One* , *15* (6), e0233839. 10.1371/journal.pone.0233839 32502193 PMC7274400

[cit0021] Dwyer, J. (2004). Illegal immigrants, health care, and social responsibility. *Hastings Center Report* , *34* (1), 34–41. 10.2307/3528249 15098405

[cit0022] El Arab, R. A. , Somerville, J. , Abuadas, F. H. , Rubinat-Arnaldo, E. , & Sagbakken, M. (2023). Health and well-being of refugees, asylum seekers, undocumented migrants, and internally displaced persons under COVID-19: A scoping review. *Frontiers in public health* , *11* , 1145002. 10.3389/fpubh.2023.1145002 37181725 PMC10169615

[cit0023] Fabi, Rachel E. , & Taylor, Holly A. (2019). Prenatal care for undocumented immigrants: professional norms, ethical tensions, and practical workarounds. *Journal of Law, Medicine & Ethics* , *47* (3), 398–408. 10.1177/1073110519876172 PMC10119784 31560623

[cit0024] Gely, Y. I. , Esqueda-Medina, M. , Johnson, T. J. , Arias-Pelayo, M. L. , Cortes, N. A. , Isgor, Z. , Lynch, E. B. , & Lange-Maia, B. S. (2023). Experiences with kidney transplant among undocumented immigrants in Illinois: A qualitative study. *Kidney Medicine* , *5* (6), 100644. 10.1016/j.xkme.2023.100644 37235043 PMC10206204

[cit0025] Goldade, K. (2009). Health is hard here” or “health for all”? the politics of blame, gender, and health care for undocumented Nicaraguan migrants in Costa Rica. *Medical Anthropology Quarterly* , *23* (4), 483–503. 10.1111/j.1548-1387.2009.01074.x 20092055

[cit0026] Gottlieb, N. , Trummer, U. , Davidovitch, N. , Krasnik, A. , Juárez, S. P. , Rostila, M. , Biddle, L. , & Bozorgmehr, K. (2020). Economic arguments in migrant health policymaking: proposing a research agenda. *Globalization and Health* , *16* , 1–5. 10.1186/s12992-020-00642-8 33218359 PMC7677743

[cit0027] Graneheim, U. H. , & Lundman, B. (2004). Qualitative content analysis in nursing research: concepts, procedures and measures to achieve trustworthiness. *Nurse education today* , *24* (2), 105–112. 10.1016/j.nedt.2003.10.001 14769454

[cit0028] Granero-Molina, J. , Jiménez‐Lasserrotte, M. D. M. , Fernández‐Medina, I. M. , Ruiz‐Fernández, M. D. , Hernández‐Padilla, J. M. , & Fernández‐Sola, C. (2022). Nurses’ experiences of emergency care for undocumented migrants who travel by boats. *International nursing review* , *69* (1), 69–79. 10.1111/inr.12723 34628657

[cit0029] Granero-Molina, J. , del, M. , Jimenez-Lasserrotte, M. , Ruiz-Fernández, M. D. , Hernández-Padilla, J. M. , Fernández-Medina, I. M. , del, M. , Lopez-Rodriguez, M. , & Fernández-Sola, C. (2021). Physicians’ experiences of providing emergency care to undocumented migrants arriving in Spain by small boats. *International emergency nursing* , *56* , 101006. 10.1016/j.ienj.2021.101006 33989922

[cit0030] Griffin, B. J. , Purcell, N. , Burkman, K. , Litz, B. T. , Bryan, C. J. , Schmitz, M. , Villierme, C. , Walsh, J. , & Maguen, S. (2019). Moral injury: an integrative review. *Journal of traumatic stress* , *32* (3), 350–362. 10.1002/jts.22362 30688367

[cit0031] Grove, N. J. , & Zwi, A. B. (2006). Our health and theirs: forced migration, othering, and public health. *Social science & medicine* , *62* (8), 1931–1942. 10.1016/j.socscimed.2005.08.061 16242227

[cit0032] Gullberg, F. , & Wihlborg, M. (2014). Nurses’ experiences of encountering undocumented migrants in Swedish emergency healthcare. *International Journal of Migration, Health and Social Care* , *10* (3), 148–158. 10.1108/IJMHSC-08-2013-0027

[cit0033] Hodkinson, A. , Zhou, A. , Johnson, J. , Geraghty, K. , Riley, R. , Zhou, A. , Panagopoulou, E. , Chew-Graham, C. A. , Peters, D. , Esmail, A. , & Panagioti, M. (2022). Associations of physician burnout with career engagement and quality of patient care: systematic review and meta-analysis. *BMJ* , *378* , e070442. 10.1136/bmj-2022-070442 36104064 PMC9472104

[cit0034] Hoekstra, E. (2021). Not a free version of a broken system:&Rdquo; medical humanitarianism and immigrant health justice in the United States. *Social Science & Medicine* , *285* , 114287. 10.1016/j.socscimed.2021.114287 34364157

[cit0035] Holmes, S. M. (2012). The clinical gaze in the practice of migrant health: Mexican migrants in the United States. *Social science & medicine* , *74* (6), 873–881. 10.1016/j.socscimed.2011.06.067 21992736

[cit0036] Holmes, S. M. , Castañeda, E. , Geeraert, J. , Castaneda, H. , Probst, U. , Zeldes, N. , Willen, S. S. , Dibba, Y. , Frankfurter, R. , Lie, A. K. , & Askjer, J. F. (2021). Deservingness: migration and health in social context. *BMJ global health* , *6* (Suppl 1), e005107. 10.1136/bmjgh-2021-005107 PMC8031028 33827795

[cit0037] Humikowski, C. A. (2018). Beyond burnout. *JAMA: the Journal of the American Medical Association* , *320* (4), 343–344. 10.1001/jama.2018.9910 30043069

[cit0038] Jawed, A. , Moe, S. M. , Anderson, M. , Slaven, J. E. , Wocial, L. D. , Saeed, F. , & Torke, A. M. (2021). High moral distress in clinicians involved in the care of undocumented immigrants needing dialysis in the United States. *Health equity* , *5* (1), 484–492. 10.1089/heq.2020.0114 34316532 PMC8309436

[cit0039] Jensen, N. K. , Norredam, M. , Draebel, T. , Bogic, M. , Priebe, S. , & Krasnik, A. (2011). Providing medical care for undocumented migrants in Denmark: what are the challenges for health professionals? *BMC Health Services Research* , *11* , 1–10. 10.1186/1472-6963-11-154 21711562 PMC3150245

[cit0040] Jiménez-Lasserrotte, M. D. M. , Artés-Navarro, R. , Granero-Molina, J. , Fernández-Medina, I. M. , Ruiz-Fernández, M. D. , & Ventura-Miranda, M. I. (2023). Experiences of healthcare providers who provide emergency care to migrant children who arriving in Spain by small boats (patera): A qualitative study. *Children* , *10* (6), 1079. 10.3390/children10061079 37371310 PMC10296883

[cit0041] Juárez, S. P. , Honkaniemi, H. , Dunlavy, A. C. , Aldridge, R. W. , Barreto, M. L. , Katikireddi, S. V. , & Rostila, M. (2019). Effects of non-health-targeted policies on migrant health: A systematic review and meta-analysis. *The Lancet Global Health* , *7* (4), e420–e435. 10.1016/S2214-109X(18)30560-6 30852188 PMC6418177

[cit0042] Kim, G. , Molina, U. S. , & Saadi, A. (2019). Should immigration status information be included in a patient’s health record? *AMA Journal of ethics* , *21* (1), 8–16. 10.1001/amajethics.2019.8 30672413

[cit0043] Kisa, S. , & Kisa, A. (2024). No papers, no Treatment": A scoping review of challenges faced by undocumented immigrants in accessing emergency healthcare. *International Journal for Equity in Health* , *23* (1), 184. 10.1186/s12939-024-02270-9) 39277719 PMC11401389

[cit0044] Kluesner, N. H. , McGrath, N. , Allen, N. G. , Dilip, M. , & Brenner, J. (2021). Ethical issues and obligations with undocumented immigrants relying on emergency departments for dialysis. *JACEP Open* , *2* (6), e12590. 10.1002/emp2.12590 35005702 PMC8716567

[cit0045] Kuczewski, M. (2019). Clinical ethicists awakened: addressing two generations of clinical ethics issues involving undocumented patients. *The American Journal of Bioethics* , *19* (4), 51–57. 10.1080/15265161.2019.1572812 30994422

[cit0046] Kvamme, E. , & Voldner, N. (2022). Public health nurses’ encounters with undocumented migrant mothers and children. *Public health nursing* , *39* (1), 286–295. 10.1111/phn.13019 34897781

[cit0047] Lafaut, D. (2021). Beyond biopolitics: the importance of the later work of foucault to understand care practices of healthcare workers caring for undocumented migrants. *BMC Medical Ethics* , *22* (1), 157. 10.1186/s12910-021-00726-z 34837977 PMC8627089

[cit0048] Levesque, J. F. , Harris, M. F. , & Russell, G. (2013). Patient-centred access to health care: conceptualising access at the interface of health systems and populations. *International journal for equity in health* , *12* (1), 18. 10.1186/1475-9276-12-18 23496984 PMC3610159

[cit0049] Lipsky, M. (2010). The Critical Role of Street-Level Bureaucrats, Street-level bureaucracy: Dilemmas of the individual in public services. 30th Anniversary Expanded Edition (pp. 3–12). Russell Sage Foundation.

[cit0050] Lo, M. C. M. , & Nguyen, E. T. (2021). Resisting the racialization of medical deservingness: how latinx nurses produce symbolic resources for latinx immigrants in clinical encounters. *Social Science & Medicine* , *270* , 113677. 10.1016/j.socscimed.2021.113677 33434715

[cit0051] López-Domene, E. , Granero-Molina, J. , Fernández-Sola, C. , Hernández-Padilla, J. M. , López-Rodríguez, M. D. M. , Fernández-Medina, I. M. , Guerra-Martín, M. D. , & Jiménez-Lasserrrotte, M. D. M. (2019). Emergency care for women irregular migrants who arrive in Spain by small boat: A qualitative study. *International journal of environmental research and public health* , *16* (18), 3287. 10.3390/ijerph16183287 31500213 PMC6765787

[cit0052] Marrow, H. B. (2012). Deserving to a point: unauthorized immigrants in San Francisco’s universal access healthcare model. *Social Science & Medicine* , *74* (6), 846–854. 10.1016/j.socscimed.2011.08.001 21893374

[cit0053] Martinez, O. , Wu, E. , Sandfort, T. , Dodge, B. , Carballo-Dieguez, A. , Pinto, R. , Rhodes, S. , Moya, E. , & Chavez-Baray, S. (2015). Evaluating the impact of immigration policies on health status among undocumented immigrants: A systematic review. *Journal of immigrant and minority health* , *17* , 947–970. 10.1007/s10903-013-9968-4 24375382 PMC4074451

[cit0054] Mladovsky, P. (2023). Mental health coverage for forced migrants: managing failure as everyday governance in the public and NGO sectors in england. *Social Science & Medicine* , *319* , 115385. 10.1016/j.socscimed.2022.115385 36175262

[cit0055] Moezzi, S. M. I. , Etemadi, M. , Lankarani, K. B. , Behzadifar, M. , Katebzada, H. , & Shahabi, S. (2024). Barriers and facilitators to primary healthcare utilization among immigrants and refugees of low and middle-income countries: A scoping review. *Globalization and health* , *20* (1), 75. 10.1186/s12992-024-01079-z 39449084 PMC11515291

[cit0056] Moher, D. , Liberati, A. , Tetzlaff, J. , & Altman, D. G. (2009). Preferred reporting items for systematic reviews and meta-analyses: the PRISMA statement. *BMJ* , *339* , b2535–b2535. 10.1136/bmj.b2535 19622551 PMC2714657

[cit0057] Morera, D. , Delgado, J. , Lorenzo, E. , de Castro-Peraza, M. E. , & Delgado, N. (2024). Superheroes? No, thanks.” accepting vulnerability in healthcare professionals. *Human Resources for Health* , *22* (1), 16. 10.1186/s12960-024-00899-9 38378609 PMC10877781

[cit0058] Onarheim, K. H. , Melberg, A. , Meier, B. M. , & Miljeteig, I. (2018). Towards universal health coverage: including undocumented migrants. *BMJ global health* , *3* (5), e001031. 10.1136/bmjgh-2018-001031 PMC6195153 30364297

[cit0059] Onarheim, K. H. , Wickramage, K. , Ingleby, D. , Subramani, S. , & Miljeteig, I. (2021). Adopting an ethical approach to migration health policy, practice and research. *BMJ global health* , *6* (7), e006425. 10.1136/bmjgh-2021-006425 PMC8319989 34321236

[cit0060] Ooms, G. , Keygnaert, I. , & Hammonds, R. (2019). The right to health: from citizen's right to human right (and back). *Public Health* , *172* , 99–104. 10.1016/j.puhe.2019.01.019 30905443

[cit0061] Papageorgiou, V. , Wharton-Smith, A. , Campos-Matos, I. , & Ward, H. (2020). Patient data-sharing for immigration enforcement: A qualitative study of healthcare providers in england. *BMJ Open* , *10* (2), e033202. 10.1136/bmjopen-2019-033202 PMC7044876 32051313

[cit0062] Piccoli, L. , & Perna, R. (2024). Civil society organisations and the healthcare of irregular migrants: the humanitarianism-equity dilemma. *Comparative migration studies* , *12* (1), 20. 10.1186/s40878-024-00372-5

[cit0063] Poškutė, M. , Bartkienė, A. , Fatkulina, N. , & Gefenas, E. (2022). The contribution of professional autonomy in advancing ethical behaviour: A narrative review of studies in nursing. *Journal of Nursing Management* , *30* (7), 2301–2307. 10.1111/jonm.13842 36192841

[cit0064] Pursio, K. , Kankkunen, P. , Sanner‐Stiehr, E. , & Kvist, T. (2021). Professional autonomy in nursing: an integrative review. *Journal of nursing management* , *29* (6), 1565–1577. 10.1111/jonm.13282 33548098

[cit0065] Richard, P. J. , & Brisbois, M. D. (2019). Deportation and health: implications for nurses. *Nursing2024* , *49* (6), 64–66. 10.1097/01.NURSE.0000558095.53840.e9 31124859

[cit0066] Roberts, M. L. A. , & Schiavenato, M. (2017). Othering in the nursing context: A concept analysis. *Nursing Open* , *4* (3), 174–181. 10.1002/nop2.82 28694982 PMC5500989

[cit0067] Roberts, F. , Teague, B. , Lee, J. , & Rushworth, I. (2021). The prevalence of burnout and secondary traumatic stress in professionals and volunteers working with forcibly displaced people: A systematic review and two meta‐analyses. *Journal of traumatic stress* , *34* (4), 773–785. 10.1002/jts.22659 33772884

[cit0068] Ruiz-Casares, M. , Rousseau, C. , Laurin-Lamothe, A. , Rummens, J. A. , Zelkowitz, P. , Crépeau, F. , & Steinmetz, N. (2013). Access to health care for undocumented migrant children and pregnant women: the paradox between values and attitudes of health care professionals. *Maternal and child health journal* , *17* , 292–298. 10.1007/s10995-012-0973-3 22399247

[cit0069] Saadi, A. , Sanchez Molina, U. , Franco-Vasquez, A. , Inkelas, M. , & Ryan, G. W. (2021). Barriers and facilitators to implementation of health system interventions aiming to welcome and protect immigrant patients: A qualitative study. *Journal of General Internal Medicine* , *36* (10), 3071–3079. 10.1007/s11606-021-06788-4 33987786 PMC8118102

[cit0070] Sahraoui, N. (2020). Challenges to medical ethics in the context of detention and deportation: insights from a French postcolonial department in the Indian Ocean. *Social Science & Medicine* , *258* , 113073. 10.1016/j.socscimed.2020.113073 32480185

[cit0071] Sandblom, M. , & Mangrio, E. (2017). The experience of nurses working within a voluntary network: A qualitative study of health care for undocumented migrants. *Scandinavian Journal of Caring Sciences* , *31* (2), 285–292. 10.1111/scs.12343 27292203

[cit0072] Scott, R. , Forde, E. , & Wedderburn, C. (2019). GP trainees’ experience, knowledge and attitudes towards caring for refugees, asylum seekers and undocumented migrants. *Education for Primary Care* , *30* (5), 322–323. 10.1080/14739879.2019.1652699 31409205

[cit0073] Serre-Delcor, N. , Oliveira, I. , Moreno, R. , Treviño, B. , Hajdók, E. , Esteban, E. , Murias-Closas, A. , Denial, A. , & Evangelidou, S. (2021). A cross-sectional survey on professionals to assess health needs of newly arrived migrants in Spain. *Frontiers in public health* , *9* , 667251. 10.3389/fpubh.2021.667251 34409005 PMC8365167

[cit0074] Smith, C. (2020). The structural vulnerability of healthcare workers during COVID-19: observations on the social context of risk and the equitable distribution of resources. *Social Science & Medicine* , *258* , 113119. 10.1016/j.socscimed.2020.113119 32534301 PMC7280115

[cit0075] Stephen, J. M. , & Zoucha, R. (2021). A call for nurse leader action: ethical nursing care of latinx unauthorized immigrant children and families. *Nurse leader* , *19* (4), 395–400. 10.1016/j.mnl.2020.08.002 PMC7492073 32952460

[cit0076] Straßmayr, C. , Matanov, A. , Priebe, S. , Barros, H. , Canavan, R. , Díaz-Olalla, J. M. , Gabor, E. , Gaddini, A. , Greacen, T. , Holcnerová, P. , & Kluge, U. (2012). Mental health care for irregular migrants in Europe: barriers and how they are overcome. *BMC Public Health* , *12* , 1–12. 10.1016/j.mnl.2020.08.002 22607386 PMC3528475

[cit0077] Suphanchaimat, R. , Kantamaturapoj, K. , Putthasri, W. , & Prakongsai, P. (2015). Challenges in the provision of healthcare services for migrants: A systematic review through providers’ lens. *BMC health services research* , *15* , 1–14. 10.1186/s12913-015-1065-z 26380969 PMC4574510

[cit0078] Tan, L. , Le, M. K. , Yu, C. C. , Liaw, S. Y. , Tierney, T. , Ho, Y. Y. , Low, J. , Lim, E. , Ng, R. , & Ngeow, C. (2021). Defining clinical empathy: A grounded theory approach from the perspective of healthcare workers and patients in a multicultural setting. *BMJ Open* , *11* (9), e045224. 10.1136/bmjopen-2020-045224 PMC8442049 34521657

[cit0079] Teunissen, E. , Van Bavel, E. , Van Den Driessen Mareeuw, F. , Macfarlane, A. , Van Weel-Baumgarten, E. , Van Den Muijsenbergh, M. , & Van Weel, C. (2015). Mental health problems of undocumented migrants in the Netherlands: A qualitative exploration of recognition, recording, and treatment by general practitioners. *Scandinavian Journal of Primary Health Care* , *33* (2), 82–90. 10.3109/02813432.2015.1041830 25961462 PMC4834507

[cit0080] Tiedje, K. , & Plevak, D. J. (2014). Medical humanitarianism in the United States: alternative healthcare, spirituality and political advocacy in the case of our lady guadalupe free clinic. *Social Science & Medicine* , *120* , 360–367. 10.1016/j.socscimed.2014.05.018 24862175

[cit0081] Tschirhart, N. , Jiraporncharoen, W. , Thongkhamcharoen, R. , Yoonut, K. , Ottersen, T. , & Angkurawaranon, C. (2021). Including undocumented migrants in universal health coverage: A maternal health case study from the Thailand-Myanmar border. *BMC Health Services Research* , *21* , 1–9. 10.1186/s12913-021-07325-z 34876107 PMC8650330

[cit0082] van Midde, M. , Hesse, I. , van Der Heijden, G. J. , Duijster, D. , van Elteren, M. , Kroesen, M. , Agyemang, C. , & Beune, E. (2021). Access to oral health care for undocumented migrants: perspectives of actors involved in a voluntary dental network in the Netherlands. *Community Dentistry and Oral Epidemiology* , *49* (4), 330–336. 10.1111/cdoe.12605 33341949

[cit0083] Vanobberghen, R. , Lafaut, D. , Louckx, F. , Devroey, D. , & Vandevoorde, J. (2022). Between sympathy, fascination, and powerlessness. The experiences of health professionals during the medical monitoring of a hunger strike among undocumented migrants. *Frontiers in public health* , *10* , 756964. 10.3389/fpubh.2022.756964 35692350 PMC9174691

[cit0084] Vanthuyne, K. , Meloni, F. , Ruiz-Casares, M. , Rousseau, C. , & Ricard-Guay, A. (2013). Health workers' perceptions of access to care for children and pregnant women with precarious immigration status: health as a right or a privilege? *Social science & medicine* , *93* , 78–85. 10.1016/j.socscimed.2013.06.008 23906124

[cit0085] Voldner, N. , Eick, F. , & Vangen, S. (2023). Goodbye and good luck’Midwifery care to pregnant undocumented migrants in Norway: A qualitative study. *Sexual & Reproductive Healthcare* , *37* , 100878. 10.1016/j.srhc.2023.100878 37369145

[cit0086] Wade, G. H. (1999). Professional nurse autonomy: concept analysis and application to nursing education. *Journal of advanced nursing* , *30* (2), 310–318. 10.1046/j.1365-2648.1999.01083.x 10457232

[cit0087] Willen, S. S. (2011). Do “Illegal” im/migrants have a right to health? Engaging ethical theory as social practice at a tel aviv open clinic. *Medical anthropology quarterly* , *25* (3), 303–330. 10.1111/j.1548-1387.2011.01163.x 22007560

[cit0088] Woodward, A. , Howard, N. , & Wolffers, I. (2014). Health and access to care for undocumented migrants living in the european union: A scoping review. *Health policy and planning* , *29* (7), 818–830. Epub 2013 Aug 16. 10.1093/heapol/czt061 23955607 PMC4186209

[cit0089] Woofter, R. , & Sudhinaraset, M. (2022). Differences in barriers to healthcare and discrimination in healthcare settings among undocumented immigrants by deferred action for childhood arrivals (DACA) status. *Journal of Immigrant and Minority Health* , *24* (4), 937–944. 10.1007/s10903-022-01346-4 35226220 PMC9256563

[cit0090] Worthing, K. , Seta, P. , Ouwehand, I. , Berlin, A. , & Clinch, M. (2022). Reluctance of general practice staff to register patients without documentation: A qualitative study in north east London. *British Journal of General Practice* , *73* , e276–e283. 10.3399/BJGP.2022.0336 PMC9762756 36997202

[cit0091] Yu, M. , Kelley, A. T. , Morgan, A. U. , Duong, A. , Mahajan, A. , & Gipson, J. D. (2020). Challenges for adult undocumented immigrants in accessing primary care: A qualitative study of health care workers in Los Angeles county. *Health Equity* , *4* (1), 366–374. 10.1089/heq.2020.0036 32923841 PMC7484891

