# Supplementary material for: Between duty and constraint: a qualitative systematic review of healthcare providers' ethical challenges and moral stressors in caring for undocumented migrants
Source: Int J Qual Stud Health Well-being. 2026 Jul 9;21(1):2701615. doi: 10.1080/17482631.2026.2701615 (PMC13353462; doi:10.1080/17482631.2026.2701615)
Supplement: Healthcare policy contexts.docx [file ZQHW_A_2701615_SM5347.docx]

**Healthcare policy contexts for undocumented migrants across the included countries**

The studies included in this review span diverse healthcare contexts across North America, Europe, Central America, and Western and Southeast Asia. These contexts differ markedly in their healthcare system structures and in the entitlements granted to undocumented migrants—ranging from highly restrictive, emergency-only models to more inclusive approaches that extend access to additional services, such as maternal and child care. This supplementary material provides a brief overview of these policy contexts, organized by country, to contextualize the healthcare environments represented in the included studies.

**North America (United States)**

The United States lacks a universal healthcare system; its predominantly market-based model and the Affordable Care Act exclude undocumented migrants from both Medicaid eligibility and Marketplace coverage, leaving many uninsured. These gaps are further entrenched by federal and state policy decisions: Congress prohibits undocumented migrants from accessing premium tax credits, restricts subsidies and cost-sharing reductions to those with incomes below 400% of the federal poverty level, and, following the 2012 Supreme Court ruling that made Medicaid expansion optional, several states chose not to broaden eligibility (Crowley et al., 2020; Castañeda & Mulligan, 2017).

Access to care is therefore anchored in emergency-only entitlements: hospitals must stabilize patients under the Emergency Medical Treatment and Labor Act, and Emergency Medicaid covers labor and emergent conditions but not ongoing treatment. Limited federally supported services exist for children and prenatal care, while safety-net delivery is provided by Federally Qualified Health Centers and free or low-cost clinics that serve patients regardless of immigration status (Castañeda & Mulligan, 2017; Beck et al., 2019).

Subnational policies create heterogeneity: as of April 2025, 14 states and the District of Columbia provide comprehensive state-funded coverage to children regardless of immigration status; New Jersey and Vermont extend state-funded coverage to income-eligible pregnant people (with Vermont providing 12 months of postpartum coverage); and seven states plus the District of Columbia offer fully state-funded coverage to at least some income-eligible adults regardless of status. These state actions are associated with lower uninsurance among immigrants and, for pregnancy expansions, greater prenatal care use and improved birth outcomes (Key facts on health coverage of immigrants, 2025).

However, exclusionary federal policies and enforcement measures—such as the 2019 expansion of the “public charge” rule (partly reversed in 2021) and programs like 287(g)—have generated chilling effects, prompting even eligible family members to avoid services (Nuño et al., 2022). Concerns about accessing assistance programs, including health coverage, are likely to intensify under the second Trump administration. Moreover, provisions currently under consideration in the House budget reconciliation bill could restrict health coverage for undocumented migrants and penalize states that use their own funds to cover immigrants regardless of status (Key facts on health coverage of immigrants, 2025).

**Europe**

Moving to Europe, healthcare entitlements for undocumented migrants are determined nationally and span a wide spectrum—from emergency-only access to broader schemes that include primary and secondary care. This diversity results in marked cross-country variability, with frequent legal and administrative restrictions limiting effective access. Differences are evident not only in legislative frameworks but also across healthcare levels, processes of care, and even in which subgroups of undocumented migrants are deemed eligible. In many countries, a clear gap exists between international human rights obligations and the degree to which healthcare entitlements for undocumented migrants are formally recognized (Suess et al., 2014).

Entitlements for children in an irregular migrant situation are especially heterogeneous: while France, Italy, Norway, Portugal, and Spain guarantee equal entitlements to all migrant children regardless of status, many other countries provide narrower or more ambiguous coverage (Stubbe Østergaard et al., 2017). Moreover, even where entitlements are formally recognized, their implementation mechanisms vary, and undocumented migrants remain among those most left behind in the WHO European Region’s pursuit of universal health coverage (Stevenson et al., 2024). We now outline country-level contexts within Europe.

**Western Europe (Spain, Belgium, France, Germany)**

**Spain**

Prior to 2012, Spain’s national health system afforded near-universal coverage; undocumented migrants registered at the municipal level had rights to care comparable to nationals under Law 4/2000 and subsequent reforms (Terraza Núñez et al., 2010; Vázquez et al., 2011). In April 2012, Royal Decree-Law (RDL) 16/2012 (with RD 1192/2012) reversed this approach by linking entitlement to social-security status and restricting undocumented migrants to emergency services, maternity and childbirth care, and healthcare for children, who remained entitled to equal health rights as citizen children (Cimas et al., 2016; Gogishvili et al., 2021; Hjern & Østergaard, 2016).

Given Spain’s decentralized governance, autonomous communities diverged sharply: several countered or attenuated the decree through legal, legislative, or administrative measures (e.g., alternative pathways, varying residency requirements), while others implemented the restrictions as written—producing substantial within-country heterogeneity in access (Cimas et al., 2016; Gogishvili et al., 2021). The 2018 RDL sought to re-expand access beyond emergencies, but implementation has remained uneven—residency-proof requirements (e.g., three months), jurisdictional ambiguities, paperwork hurdles, and variation across autonomous communities continue to impede effective entitlement, with non-governmental organizations frequently bridging the gaps (Hsia & Gil-González, 2021; Pérez-Urdiales, 2021).

**Belgium**

Belgium entitles undocumented migrants, including children, to Urgent Medical Aid (Aide Médicale Urgente, AMU)—a statutory safety net that provides free preventive, curative, outpatient, and hospital care to those without regular insurance. Enrollment in AMU requires an administrative procedure managed by the Public Centers for Social Welfare (CPAS/OCMW), which verify irregular status and medical need. A persistent challenge is the legally undefined scope of “medical urgency”, which must be assessed and attested by the consulting healthcare provider (Roberfroid et al., 2015).

This ambiguity not only influences how undocumented migrants seek care but also shapes the decision-making of healthcare providers and CPAS/OCMW, at times resulting in suboptimal care, refusal of care, or inadequate recognition of serious health conditions—particularly those related to mental health. Certain groups, including pregnant women, newborns, and children, are especially vulnerable to health risks, yet often receive insufficient care (Roberfroid et al., 2015).

Taken together, the restrictive interpretation of “medical urgency” and the disproportionate vulnerability of these groups underscore the structural limits of AMU. These limits are further reinforced by administrative complexity, lengthy social inquiries, and persistent information gaps, which often delay coverage. For instance, in a large Brussels maternity cohort, only 22% of AMU recipients had coverage at the first prenatal visit; common reasons for lacking coverage included waiting for a CPAS decision (40%), not being referred (26%), or not having applied (18%). Women without any coverage were more likely to deliver prematurely and to have newborns with lower birth weight compared to those with AMU or regular insurance (Vanneste et al., 2020).

**France**

In France, since 2000, undocumented migrants have been entitled to State Medical Aid (Aide Médicale de l’État, AME), which provides renewable one-year coverage for all medical treatment related to illness or childbirth within the tariffs set by Social Security. Eligibility requires meeting income thresholds, demonstrating at least three months of residence, and submitting supporting documentation. All healthcare professionals are legally obliged to accept AME beneficiaries. Those unable to satisfy the residency requirement are limited to Urgent and Vital Care (UVC)—defined as care whose absence would endanger life, cause serious long-term harm, or affect an unborn child—as well as interventions to prevent the transmission of communicable diseases (André & Azzedine, 2016). Children of irregular third-country migrants are, in principle, entitled to the same healthcare as national children. However, they are excluded from the national health insurance system and must instead qualify for AME (Hjern & Østergaard, 2016).

In practice, however, these legal entitlements have not translated into universal coverage. Analyses of the Premiers Pas survey found that only about half of eligible undocumented migrants were covered, with informational and administrative barriers identified as central reasons for non-take-up (Dourgnon et al., 2023). National hospital data (2013–2021) indicate that under-enrolment shifts care to UVC, where stays are 13% costlier, 19% longer, more likely to involve intensive care unit admission, and more often admitted via emergency departments compared with stays covered by AME (Zarca et al., 2025).

Political debate over France’s State Medical Aid intensified in late 2023. In November, the Senate approved a bill to restrict AME to serious illnesses, acute pain, pregnancy-related care, and limited preventive costs, with further parliamentary scrutiny to follow. At the same time, official budget reporting indicated that about 400,000 people benefited from AME in 2022, at an estimated 0.47% of national health-care expenditure. Such curtailment would likely delay access to care or lead individuals to forgo it, increase reliance on already overstretched emergency departments, and raise hospitalization rates while adding pressure on non-governmental organizations (Cailhol et al., 2024).

**Germany**

In Germany, while health insurance has been legally compulsory for residents since 2009, a significant uninsured population—including undocumented migrants—persists due to legal restrictions and practical barriers (Bosch & Gottlieb, 2019; Mylius & Frewer, 2015). Under the Asylum Seekers Benefits Act (AsylbLG), undocumented migrants are entitled during their first 18 months of residency to basic healthcare services, including treatment of acute illness and painful conditions, antenatal and postnatal care, vaccinations, and preventive testing with anonymous counselling and screening for infectious and sexually transmitted diseases (Bosch & Gottlieb, 2019; Webb et al., 2022). Claims typically pass through social welfare offices, which, under the Residence Act, are obliged to report undocumented migrants to immigration authorities, thereby increasing the risk of deportation and leaving many de facto without coverage (Webb et al., 2022; Mylius & Frewer, 2015).

German health policy seldom differentiates between adult and child migrants, and comparative analyses drawing on the United Nations Convention on the Rights of the Child framework identify Germany as one of the most restrictive contexts in Europe for undocumented children, where healthcare entitlements are limited (Stubbe Østergaard et al., 2017). Under the AsylbLG, undocumented children are formally entitled to emergency treatment, but access to routine and preventive services remains sharply constrained. The obligation of welfare offices to share information with immigration authorities exposes families to detection, deterring care-seeking and undermining children’s fundamental right to health. In addition, administrative procedures during and after arrival frequently disregard the best-interests-of-the-child principle—including the need for safety and stability—thereby compounding psychosocial stressors and exacerbating mental health risks (Stumpf, 2023).

In response, a parallel voluntary healthcare system has developed, such as NGO-run MediNetz initiatives and municipal healthcare vouchers (Anonymer Krankenschein, AKS). Empirical evidence shows that this system does not meet the standards of regular healthcare in Germany, with persistent gaps in quality and scope. Services often depend on donations and volunteers, remain heterogeneous, and are especially constrained in prevention and chronic disease management. AKS models perform closer to expected standards but are not yet universal (Stötzler& Kaifie, 2023; Zimmer, 2024).

**Northern Europe (Denmark, England, the Netherlands, Norway, Sweden)**

**Denmark**

In Denmark, the healthcare system is tax-funded and provides universal access to citizens and permanent residents. In contrast, undocumented migrants are legally entitled only to emergency care. However, the Danish Health Act does not clearly define what constitutes an emergency, leaving the determination to the discretion of the attending healthcare provider. Non-emergency care may be granted if referral to the migrant’s home country is deemed unreasonable, but such services are not consistently financed by the Regional Council. Another avenue is to seek treatment through the Danish Immigration Service, though this option carries the potential risk of deportation (Biswas et al., 2012).

Restricted access to non-emergency services exposes undocumented migrants to heightened risks of poor health outcomes. For instance, undocumented pregnant women frequently experience delays in accessing antenatal care, and have limited access to routine pregnancy screening (Funge et al., 2022; Wendland et al., 2016). Likewise, while Danish law accords undocumented children the same rights as Danish children, in practice, routine care often requires legal registration and financial approval, which are not feasible in most cases. Consequently, many undocumented children miss essential healthcare services, including vaccinations and developmental assessments, prompting parents to seek alternative support from organizations such as Danish Red Cross clinics (Parellada et al., 2021). Mobile and outreach programs have also played an important role in improving healthcare access for marginalized groups by offering culturally sensitive care. The AmiAmi clinic, for instance, provided services primarily to undocumented sex workers, a proportion of whom were victims of human trafficking, abuse, and violence (Knudtzen et al., 2022).

**England**

Similar to Denmark, England’s healthcare system is tax-funded and provides primary care services free of charge, either through registration with a general practitioner or by accessing the National Health Service (NHS) as a temporary patient (defined as someone staying in the area for more than 24 hours but less than three months). By law, undocumented migrants are entitled to the same rights (NHS entitlements: migrant health guide, 2025). In practice, however, uncertainty around eligibility and inconsistent administrative practices often obstruct access (Rafighi et al., 2016).

Secondary care follows a residence-based model, and those not considered ordinarily resident are subject to charges. A limited number of exemptions apply to undocumented migrants. These include rejected asylum seekers supported by the Home Office, local councils, or under part one of the Care Act 2014; victims of modern slavery or human trafficking; immigration detainees; persons receiving compulsory treatment under court orders or mental health legislation; and cases granted on humanitarian grounds by the Secretary of State. Certain medical conditions are also exempt from charging, including tuberculosis, HIV, other sexually transmitted infections, COVID-19, and conditions resulting from torture, female genital mutilation, domestic violence, or sexual violence (NHS entitlements: migrant health guide, 2025).

Beyond these exemptions, most non-emergency hospital services are chargeable at 150% of the standard NHS cost. This covers scheduled surgeries, specialist consultations, maternity services, and even follow-up appointments after emergency admissions (NHS entitlements: migrant health guide, 2025). Maternity care is classified as immediately necessary and cannot be refused, but undocumented women are billed at the higher tariff, often incurring substantial debts (Nellums et al., 2021). Undocumented children under 18 are also chargeable under the same rules, as England—unlike some devolved UK regions—does not provide universal free care for children (Guidance: rights to access healthcare, 2022). Since 2017, NHS trusts have been required to identify chargeable patients and to secure upfront payment for non-urgent care. Debts exceeding £500 are reported to the Home Office, reinforcing fears of enforcement and deportation. Although the 2017 NHS–Home Office data-sharing agreement was formally withdrawn in 2018, its legacy continues to foster mistrust among migrant communities (Health implications of the hostile environment, 2021).

**The Netherlands**

In the Netherlands, health insurance is mandatory for all residents, but the Dutch Linking Act (Koppelingswet), implemented in 1998, excluded undocumented migrants from public services, including health insurance, by linking eligibility to legal residence. Undocumented migrants, including children, remain legally entitled to necessary medical care and may register with a general practitioner. However, they must initially pay the costs themselves; if unable, providers can recover 80-100% of the expenses through a government reimbursement scheme. On paper, this framework makes primary, secondary, and tertiary care accessible, and the Netherlands is often considered to offer one of the most comprehensive packages in Europe (Biswas et al., 2012; Policy brief: towards realising health rights of undocumented people in the Netherlands, 2020).

In practice, undocumented migrants face significant challenges in accessing care. Barriers arise both on the provider side—where not all practitioners are familiar with or willing to use the reimbursement system—and on the patient side, where knowledge of rights is often limited. These factors, compounded by fears of authorities, the potential for high out-of-pocket costs, and experiences of discrimination, discourage many from seeking care even when entitled (Hintjens et al., 2020; Biswas et al., 2012). As a result, effective access remains far more restricted than the legal framework suggests. To bridge these barriers, several NGOs assumed mediating, educational, advocacy, and service-delivery roles. While these initiatives contributed to expanding access and improving navigation of the system, they also created a parallel system that operates partly outside the regular healthcare framework (Hanssen & Yerkes, 2025).

Yet even this limited and fragmented access remains vulnerable to shifts in political priorities. In October 2024, the Dutch cabinet announced a €40 million reduction in funding for essential healthcare for uninsured patients. While officially aimed at unemployed migrant workers rather than undocumented migrants specifically, the measure has been widely criticized by civil society and NGOs, who warn that it will further undermine access to care for already marginalized groups and reflects a broader exclusionary trend in Dutch healthcare policy (Cutting back on care for the uninsured is expensive and inhumane, 2024).

**Norway**

Access to healthcare for undocumented migrants is not necessarily determined by how strong or comprehensive a country’s welfare state is. For instance, Norway—despite being a well-established welfare state—offers only minimal healthcare services to undocumented migrants. This restricted access is embedded within the Nordic welfare model, which has increasingly been shaped by neoliberal orientations. The model emphasizes nationalism, economic stability, and equal protection for legal residents, while simultaneously marginalizing and excluding those designated as “the other” (Barker, 2017). Such exclusion raises important questions about Norway's commitment to international human rights, including the right to health and the broader goal of achieving universal health coverage for all, regardless of legal status (Onarheim et al., 2018; Haddeland, 2019; Stevenson et al., 2024).

The Royal Decree 1255 stipulates that everyone is entitled to emergency care—defined as situations requiring treatment within 24 hours—and to non-deferrable care, which includes medical conditions where postponement would pose an immediate risk of death, permanent severe disability, serious injury, or intense pain. In such cases, medical assessments are conducted under the presumption that the patient will leave the country within three weeks and will rely on their home country’s healthcare system. Healthcare that can be postponed for more than three weeks without escalating into an emergency falls outside the scope of this entitlement (Haddeland, 2019). With regard to children under 18, they are legally entitled to equivalent health rights and services—with low user fees (none under 16) and free specialized care. Nevertheless, undocumented children are excluded from general practitioner registration; instead, preventive and developmental care is primarily provided through Child Health Centres and school health services (Kvamme & Voldner, 2022).

Beyond the case of children, the Royal Decree sets out the general health entitlements of undocumented migrants. They are legally entitled to prenatal care, abortion, treatment for communicable diseases, and mental healthcare when their condition poses a risk to themselves or others. However, the general rule requires them to pay the full cost of healthcare services received. In the case of a medical emergency, upfront payment is not required, and the costs are reimbursed according to national monetary claims procedures. If the patient is unable to pay, healthcare institutions are obligated to cover the expenses (Haddeland, 2019; Onarheim et al., 2018). In practice, however, several barriers impede the realization of undocumented migrants’ health rights in Norway. Economic concerns, fear of being reported—especially among those evading deportation—poor language skills, and lack of awareness regarding healthcare providers’ duty of confidentiality often discourage undocumented individuals from seeking care, even when legally entitled to it, leading them instead to rely on alternative health-seeking strategies (Haddeland, 2019; Kvamme & Ytrehus, 2015).

**Sweden**

As in Norway, undocumented migrant adults in Sweden are entitled to emergency and non-deferrable healthcare—including maternity and abortion services—under the Act on Care for Persons without Documents (2013:407), which entered into force in 2013 (Health care of persons without permanent residence permits, 2020). These services are provided for a nominal fee comparable to that paid by asylum seekers. Prior to this legislation, undocumented adult migrants had no legal entitlement to subsidized healthcare and could only access emergency care at full cost (Human rights-based approach to universal health coverage inclusive of undocumented migrants, 2023).

The term “non-deferrable” is not clearly defined in the statute, creating uncertainty in implementation. Consequently, some patients are refused necessary treatment when their future residence in Sweden is unclear, particularly for interventions requiring long-duration care or aftercare, such as organ transplantation, psychiatric treatment, cancer care, and the provision of medical devices (Health care of persons without permanent residence permits, 2020).

A major implementation obstacle is limited provider awareness of the regulatory provisions, which contributes to inconsistent application. Additional barriers include communication difficulties; fear of disclosure to migration authorities and potential deportation; and, in some cases, arbitrary or neglectful provider behavior (O'Sullivan, 2024; Barkensjö et al., 2018; Mona et al., 2021). Cost considerations also weigh on clinical decision-making, influencing both prioritization and scope of care (O'Sullivan, 2024). There are also reported instances of patients being wrongly asked to reimburse costs (Health care of persons without permanent residence permits, 2020). In contrast to adults, undocumented children are entitled to the same level of healthcare as children who are citizens or lawful residents of Sweden, ensuring more comprehensive protection for this group.

**Central America (Costa Rica)**

Costa Rica is often recognized for its universal and comparatively generous healthcare system. Nevertheless, access to healthcare for undocumented migrants is constrained by a range of legal, institutional, and de facto barriers, as well as by agency practices, where migration and social policies intersect to restrict accessibility and, in effect, undermine the universalist ethos of the system (Voorend & Alvarado, 2023).

Public healthcare in Costa Rica is administered by the Costa Rican Social Security Fund (CCSS), which operates on a tripartite financing model involving contributions from employers, employees, and the state. The state also provides non-contributory insurance for individuals living below the poverty line, as well as for uninsured pregnant women and children, regardless of immigration status (Voorend & Alvarado, 2023). Article 42 of the Children and Adolescents Code guarantees that pregnant women are entitled to prenatal care throughout pregnancy and postnatal care for up to three months. Likewise, children are entitled to state coverage if their parents or guardians are uninsured, irrespective of documentation. In addition, individuals living with HIV are entitled to uninterrupted access to antiretroviral therapy and may benefit from state health insurance if they lack other forms of coverage. Emergency care is available to all uninsured individuals, including undocumented migrants; however, they are charged after receiving services (Access and right to health in Costa Rica).

Beyond such situations, access to healthcare remains heavily conditioned by migration status, as the law explicitly requires proof of legal residency for enrollment in health insurance. This creates a paradoxical “Catch-22”, in which obtaining regular migratory status requires access to health insurance, while access to health insurance presupposes legal residency. The financial burden of insurance enrollment further compounds these barriers, as undocumented migrants are disproportionately employed in the informal sector, where resources are limited (Voorend & Alvarado, 2023).

Qualitative evidence further indicates that undocumented migrants, particularly those from Nicaragua, at times encounter discriminatory attitudes and xenophobic behaviors from healthcare providers and gatekeepers (Goldade, 2009; Fouratt & Voorend, 2018). Such biases are frequently justified on the grounds that migrants disproportionately burden public health services and threaten the sustainability of the welfare system (Voorend, 2014). However, quantitative evidence contradicts this perception: one study found that incidence of migrant healthcare use was, in fact, lower than their share in the population (Voorend et al., 2021).

**Western Asia (Israel)**

Israel represents a complex, ideologically charged policy context in which undocumented (unauthorized) migrants are simultaneously marginalized, illegalized, and criminalized, and thus excluded from the national health insurance system (Willen, 2011; Willen, 2019). Since the late 1990s, Israel has seen a steady increase in undocumented residents, including a sizable population of Eritrean and Sudanese asylum seekers who are protected from refoulement despite the state’s non-recognition of their refugee claims (Willen, 2011; Waitzberg & Fialco, 2024; Moss et al., 2019).

Within this legal framework, access to publicly financed services is largely restricted to emergency care, with limited primary and specialist coverage for select conditions (e.g., maternal and infant care, infant immunizations, tuberculosis services, HIV diagnosis, and abortion in specific circumstances such as for minors or survivors of rape). Outside these narrowly defined entitlements, undocumented migrants are expected to purchase private insurance. Even when financially feasible, private coverage typically excludes pre-existing conditions, childbirth, mental health care, and rehabilitation, producing systematic gaps in access (Waitzberg & Fialco, 2024).

Until recently, children of undocumented migrants could enroll in a government-subsidized insurance scheme contingent on a monthly premium paid by parents (Moss et al., 2019). The Ministry of Health’s abrupt revision of this policy—restricting children’s access to emergency care only—has been criticized by Israeli human rights organizations for intensifying vulnerability among Eritrean and Sudanese families whose asylum applications remain unresolved and whose “temporary” non-return status is effectively indefinite. This status also generally withholds the right to work and limits access to social services, compounding health risks (Response to Israel’s reply to the UN-CESCR`s list of issues, 2019).

Beyond children, other high-risk groups include survivors of torture and trafficking, persons with disabilities and chronic conditions, women facing domestic violence, and people experiencing homelessness. Limiting access to appropriate treatment and rehabilitation for these groups undermines functional autonomy and human dignity (Response to Israel’s reply to the UN-CESCR`s list of issues, 2019). In response to state exclusion and to contest political discourses that deny migrants’ biolegitimacy, civil-society providers have established “open clinics,” most notably the Physicians for Human Rights–Israel clinic (operational since 1998). While these initiatives partially mitigate unmet needs, high patient volumes, constrained resources, and reliance on donations and volunteers mean they cannot substitute for comprehensive, rights-based coverage (Willen, 2011). Collectively, these dynamics illustrate how legal precarity, restricted entitlements, and fragmented safety-net services interact to produce structurally embedded health inequities for undocumented migrants in Israel.

**Southeast Asia (Thailand)**

Where Israel exemplifies a largely exclusionary approach that restricts entitlements to narrowly defined exceptions, Thailand—through a pragmatic recognition of migrants’ economic role—has, since the early 2000s, pursued a series of policies to protect undocumented migrants’ health and expand coverage. The Ministry of Public Health launched the voluntary, premium-based Health Insurance Card Scheme (HICS), followed by a “One-Stop Service” to register undocumented migrants and their dependents and to integrate the functions of relevant authorities (Suphanchaimat et al., 2017; Tangcharoensathien et al., 2017).

Thailand’s approach to migrant health has proceeded along two main strands. First, it has sought to extend financial risk protection through HICS. Second, it has advanced migrant-friendly service delivery through measures such as recruiting volunteer community health workers, deploying mobile clinics, installing bilingual signage in health facilities, and expanding workplace outreach (Tangcharoensathien et al., 2017). Implementation has nevertheless faced persistent challenges, including bureaucratic hurdles, limited legal mandates, weak intersectoral coordination among the ministries of interior, labor, public health, and immigration, and inconsistencies and ambiguities in policy communication (Suphanchaimat et al., 2019; Suphanchaimat et al., 2017).

Under HICS, any migrant may purchase coverage regardless of work-permit or citizenship status (Suphanchaimat et al., 2017). Enrollment requires registration at designated hospitals and completion of a health screening—typically for tuberculosis, syphilis, microfilaria, malaria, and leprosy—after which a full course of treatment is provided when indicated (Tangcharoensathien et al., 2017). The HICS benefits package is comparatively comprehensive, covering outpatient and inpatient services, emergency care, and health-promotion activities without beneficiary co-payment. A narrow exclusion list remains for selected high-cost services (e.g., dialysis for chronic renal failure) and certain psychiatric conditions (Suphanchaimat et al., 2017).

Operational issues persist. These include design gaps—particularly in financing and screening regulations that can result in individuals being deemed unfit for work and returned to their country of origin—as well as administrative delays in enrollment (Suphanchaimat et al., 2017; Tangcharoensathien et al., 2017; Kunpeuk et al., 2020). Addressing these constraints is essential if the scheme is to achieve its intended equity and public-health benefits.

**References**

United Nations High Commissioner for Refugees (UNHCR). (n.d.). Access and right to health in Costa Rica. Retrieved September 21, 2025 from: <https://help.unhcr.org/costarica/sobre-el-sitio/salud>

André, J. M., & Azzedine, F. (2016). Access to healthcare for undocumented migrants in France: a critical examination of State Medical Assistance. *Public Health Reviews. 37*(1), 5. https://doi.org/10.1186/s40985-016-0017-4

Barkensjö, M., Greenbrook, J. T., Rosenlundh, J., Ascher, H., & Elden, H. (2018). The need for trust and safety inducing encounters: a qualitative exploration of women’s experiences of seeking perinatal care when living as undocumented migrants in Sweden. *BMC pregnancy and childbirth. 18*(1), 217. https://doi.org/10.1186/s12884-018-1851-9

Barker, V. (2017). Nordic nationalism and penal order: Walling the welfare state (1st ed.). *Routledge*. https://doi.org/10.4324/9781315269795

Beck, T. L., Le, T. K., Henry-Okafor, Q., & Shah, M. K. (2019). Medical care for undocumented immigrants: national and international issues. *Physician Assistant Clinics. 4*(1), 33-45. https://doi.org/10.1016/j.cpha.2018.08.002

Biswas, D., Toebes, B., Hjern, A., Ascher, H., & Norredam, M. (2012). Access to health care for undocumented migrants from a human rights perspective: a comparative study of Denmark, Sweden, and The Netherlands. *Health Hum Rights. 14*(2), 49-60.

Bosch, S. C., & Gottlieb, D. N. (2019). Legal access rights to health care: country profile-Germany. *World Health Organization.*

Cailhol, J., Vignier, N., Vandentorren, S., Moussaoui, S., & Feral-Pierssens, A. L. (2024). French political bargaining over undocumented migrants’ health. *The Lancet. 403*(10422), 140-141. https://doi.org/10.1016/S0140-6736(23)02718-6

Castañeda, H. & Mulligan, J. (2017). Unequal Coverage: The experience of health care reform in the United States. *New York University Press.* https://doi.org/10.18574/nyu/9781479897001.001.0001

Cimas, M., Gullon, P., Aguilera, E., Meyer, S., Freire, J. M., & Perez-Gomez, B. (2016). Healthcare coverage for undocumented migrants in Spain: regional differences after Royal Decree Law 16/2012. *Health Policy. 120*(4), 384-395. https://doi.org/10.1016/j.healthpol.2016.02.005

Crowley, R., Daniel, H., Cooney, T. G., Engel, L. S., & Health and Public Policy Committee of the American College of Physicians. (2020). Envisioning a better US health care system for all: coverage and cost of care. *Annals of internal medicine, 172*(2_Supplement), S7-S32. https://doi.org/10.7326/M19-2415

Médecins Du Monde. (2024). Cutting back on care for the uninsured is expensive and inhumane. Retrieved September 13, 2025 from: https://doktersvandewereld.org/actueel/nieuws/bezuinigen-op-zorg-aan-onverzekerden-is-duur-%C3%A9n-inhumaan/

Dourgnon, P., Jusot, F., Marsaudon, A., Sarhiri, J., & Wittwer, J. (2023). Just a question of time? Explaining non-take-up of a public health insurance program designed for undocumented immigrants living in France. *Health Economics, Policy and Law. 18*(1), 32-48. https://doi.org/10.1017/S1744133122000159

Fouratt, C. E., & Voorend, K. (2018). Sidestepping the State: Practices of Social Service Commodification among Nicaraguans in Costa Rica and Nicaragua. *Journal of Latin American Studies. 50*(2), 441-468. https://doi.org/10.1017/S0022216X17001195

Funge, J. K., Boye, M. C., Parellada, C. B., & Norredam, M. (2022). Demographic characteristics, medical needs and utilisation of antenatal care among pregnant undocumented migrants living in Denmark between 2011 and 2017. *Scandinavian Journal of Public Health. 50*(5), 575-583. https://doi.org/10.1177/14034948211011400

Gogishvili, M., Costa, S. A., Flórez, K., & Huang, T. T. (2021). Policy implementation analysis on access to healthcare among undocumented immigrants in seven autonomous communities of Spain, 2012–2018. *BMJ open. 11*(6), e045626. https://doi.org/10.1136/bmjopen-2020-045626

Goldade, K. (2009). “Health is hard here” or “health for all”? The politics of blame, gender, and health care for undocumented Nicaraguan migrants in Costa Rica. *Medical Anthropology Quarterly. 23*(4), 483-503. https://doi.org/10.1111/j.1548-1387.2009.01074.x

Royal College of Paediatrics and Child Health (RCPCH). (2022). Guidance: rights to access healthcare. Retrived September 6, 2024 from: https://www.rcpch.ac.uk/sites/default/files/2022-04/Guidance-Rights_to_access_healthcare_updated_April_2022.pdf

Haddeland, H. B. (2019). The right to health Care for Irregular Migrants in Norway: interpretation, accessibility, and gaps between needs and rights. *Nordic Journal of Human Rights. 37*(4), 329-349. https://doi.org/10.1080/18918131.2020.1731970

Hanssen, I. R., & Yerkes, M. A. (2025). Health is wealth and documents are the currency: exploring the role of NGOs in facilitating healthcare access for undocumented migrants in the Netherlands–a qualitative study. *International journal for equity in health. 24*(1), 219. https://doi.org/10.1186/s12939-025-02594-0

The Swedish Council on Medical Ethics. (2020). Health care of persons without permanent residence permits. Retrieved September 20, 2025 from: https://smer.se/wp-content/uploads/2021/04/smer-rapport-2020_6-engelsk-oversattning-webb.pdf

British Medical Association. (2021). Health implications of the hostile environment. Retrieved September 20, 2025 from: https://www.bma.org.uk/media/4927/bma-health-implications-of-the-hostile-environment-dec-2021.pdf

International Institute of Social Studies. (2020). Policy brief: towards realising health rights of undocumented people in the Netherlands. Retrieved September 13, 2025 from: https://www.iss.nl/en/media/2020-09-33321researchbriefhealthrightsundocumentedpeople31-final-edited-17sep2020

Hintjens, H. M., Siegmann, K. A., & Staring, R. H. (2020). Seeking health below the radar: Undocumented People's access to healthcare in two Dutch cities. *Social Science & Medicine. 248*, 112822. https://doi.org/10.1016/j.socscimed.2020.112822

Hjern, A., & Østergaard, L. S. (2016). Migrant children in Europe: entitlements to health care. *Models of Child Health Appraised (MOCHA) Consortium*.

Hsia, R. Y., & Gil-González, D. (2021). Perspectives on Spain’s legislative experience providing access to healthcare to irregular migrants: a qualitative interview study. *BMJ open. 11*(8), e050204. https://doi.org/10.1136/bmjopen-2021-050204

Platform for International Cooperation on Undocumented Migrants (PICUM). (2023). Human rights-based approach to universal health coverage inclusive of undocumented migrants. Retrieved September 20, 2025 from: https://picum.org/wp-content/uploads/2023/08/PICUM-Submission-on-Universal-Health-Coverage.pdf

Kaiser Family Foundation (KFF). (2025). Key facts on health coverage of immigrants. Retrieved August 26, 2025 from: <https://www.kff.org/racial-equity-and-health-policy/key-facts-on-health-coverage-of-immigrants/>

Knudtzen, F. C., Mørk, L., Nielsen, V. N., & Astrup, B. S. (2022). Accessing vulnerable undocumented migrants through a healthcare clinic including a community outreach programme: a 12-year retrospective cohort study in Denmark. *Journal of Travel Medicine. 29*(7), taab128. https://doi.org/10.1093/jtm/taab128

Kunpeuk, W., Teekasap, P., Kosiyaporn, H., Julchoo, S., Phaiyarom, M., Sinam, P., Pudpong, N., & Suphanchaimat, R. (2020). Understanding the problem of access to public health insurance schemes among cross-border migrants in Thailand through systems thinking. *International journal of environmental research and public health. 17*(14), 5113. https://doi.org/10.3390/ijerph17145113

Kvamme, E., & Voldner, N. (2022). Public health nurses’ encounters with undocumented migrant mothers and children. *Public health nursing. 39*(1), 286-295. https://doi.org/10.1111/phn.13019

Kvamme, E., & Ytrehus, S. (2015). Barriers to health care access among undocumented migrant women in Norway. *Society, Health & Vulnerability. 6*(1), 28668. https://doi.org/10.3402/shv.v6.28668

Mona, H., Andersson, L. M., Hjern, A., & Ascher, H. (2021). Barriers to accessing health care among undocumented migrants in Sweden-a principal component analysis. *BMC health services research. 21*(1), 830. https://doi.org/10.1186/s12913-021-06837-y

Moss, D., Gutzeit, Z., Mishori, R., Davidovitch, N., & Filc, D. (2019). Ensuring migrants’ right to health? Case of undocumented children in Israel. *BMJ Paediatrics Open. 3*(1), e000490. https://doi.org/10.1136/bmjpo-2019-000490

Mylius, M., & Frewer, A. (2015). Access to healthcare for undocumented migrants with communicable diseases in Germany: a quantitative study. *The European Journal of Public Health. 25*(4), 582-586. https://doi.org/10.1093/eurpub/ckv023

Nellums, L.B., Powis, J., Jones, L., Miller, A., Rustage, K., Russell, N., Friedland, J.S., & Hargreaves, S. (2021). “It's a life you're playing with”: A qualitative study on experiences of NHS maternity services among undocumented migrant women in England. *Social Science & Medicine. 270*, 113610. https://doi.org/10.1016/j.socscimed.2020.113610

Office for Health Improvement and Disparities. (2025). NHS entitlements: migrant health guide. Retrieved September 5, 2025 from: <https://www.gov.uk/guidance/nhs-entitlements-migrant-health-guide>

Nuño, L. E., Herrera, V. M., & Soto, B. S. (2022). First, do no harm: why anti-immigrant policies in the United States are a public health concern. *Journal of public health policy. 43*(3), 403-412. https://doi.org/10.1057/s41271-022-00364-8

O'Sullivan, A. (2024). Undocumented migrants’ access to healthcare in Sweden, and the impact of Act 2013: 407. *Nursing ethics. 31*(7), 1349-1360. https://doi.org/10.1177/09697330231215947

Onarheim, K. H., Melberg, A., Meier, B. M., & Miljeteig, I. (2018). Towards universal health coverage: including undocumented migrants. *BMJ global health. 3*(5). https://doi.org/10.1136/bmjgh-2018-001031

Parellada, C. B., Boye, M. C., & Norredam, M. (2021). Vaccination needs and use of preventive healthcare services among undocumented migrant children in Denmark. *Acta paediatrica. 110*(6), 1932-1934. https://doi.org/10.1111/apa.15758

Pérez-Urdiales, I. (2021). Undocumented immigrants’ and immigrant women’s access to healthcare services in the Basque Country (Spain). *Global health action. 14*(1), 1896659. https://doi.org/10.1080/16549716.2021.1896659

Rafighi, E., Poduval, S., Legido-Quigley, H., & Howard, N. (2016). National Health Service principles as experienced by vulnerable London migrants in" austerity Britain": a qualitative study of rights, entitlements, and civil-society advocacy. *International journal of health policy and management. 5*(10), 589. https://doi.org/10.15171/ijhpm.2016.50

Physicians for Human Rights and Aid Organization for Refugees and Asylum Seekers in Israel. (2019). Response to Israel’s reply to the UN-CESCR`s list of issues. Retrieved September 27, 2025 from: https://www.phr.org.il/wp-content/uploads/2019/09/Response-to-Israel%E2%80%99s-Reply-to-the-UN-CESCRs-List-of-Issues-ASSAF-PHRI-.pdf.

Roberfroid, D., Dauvrin, M., Keygnaert, I., Desomer, A., Kerstens, B., Camberlin, C., Gysen, J., Lorant, V., & Derluyn, I. (2015). What health care for undocumented migrants in Belgium?. *KCE reports. 257*.

Waitzberg, R., & Fialco, S. (2024). Can people afford to pay for health care? New evidence on financial protection in Israel. *WHO Regional Office for Europe.* Retrieved September 27, 2025 from: https://iris.who.int/server/api/core/bitstreams/8d96a36d-6b18-4deb-997a-3634c7c3a925/content

Stevenson, K., Antia, K., Burns, R., Mosca, D., Gencianos, G., Rechel, B., Norredam, M., LeVoy, M., & Blanchet, K. (2024). Universal health coverage for undocumented migrants in the WHO European region: a long way to go. *The Lancet Regional Health–Europe. 41*. https://doi.org/10.1016/j.lanepe.2023.100803

Stötzler, M., & Kaifie, A. (2023). Healthcare for individuals without health insurance in Germany–a mixed methods approach to assess the situation and current challenges. *International Journal for Equity in Health. 22*(1), 117. https://doi.org/10.1186/s12939-023-01930-6

Stubbe Østergaard, L., Norredam, M., Mock-Munoz de Luna, C., Blair, M., Goldfeld, S., & Hjern, A. (2017). Restricted health care entitlements for child migrants in Europe and Australia. *The European Journal of Public Health. 27*(5), 869-873. https://doi.org/10.1093/eurpub/ckx083

Stumpf, S. (2023). Factsheet - Universal periodic review of Germany. SOS Cildren's Villages. Retrieved October 4, 2025 from: https://upr-info.org/sites/default/files/country-document/2023-09/Fact_sheet_SOS_Kinderdorf.pdf

Suess, A., Ruiz Pérez, I., Ruiz Azarola, A., & March Cerdà, J. C. (2014). The right of access to health care for undocumented migrants: a revision of comparative analysis in the European context. *The European Journal of Public Health. 24*(5), 712-720. https://doi.org/10.1093/eurpub/cku036

Suphanchaimat, R., Pudpong, N., Prakongsai, P., Putthasri, W., Hanefeld, J., & Mills, A. (2019). The devil is in the detail—understanding divergence between intention and implementation of health policy for undocumented migrants in Thailand. *International Journal of Environmental Research and Public Health. 16*(6), 1016. https://doi.org/10.3390/ijerph16061016

Suphanchaimat, R., Putthasri, W., Prakongsai, P., & Tangcharoensathien, V. (2017). Evolution and complexity of government policies to protect the health of undocumented/illegal migrants in Thailand–the unsolved challenges. *Risk management and healthcare policy.* 49-62. https://doi.org/10.2147/RMHP.S130442

Tangcharoensathien, V., Thwin, A. A., & Patcharanarumol, W. (2017). Implementing health insurance for migrants, Thailand. *Bulletin of the World Health Organization. 95*(2), 146. https://doi.org/10.2471/BLT.16.179606

Terraza Núñez, R., Vargas Lorenzo, I., Rodríguez Arjona, D., Lizana Alcazo, T., & Vázquez Navarrete, M. L. (2009). Health policies of national and regional level for the immigrant population in Spain. *Gaceta Sanitaria. 24*(2), 115-e1. https://doi.org/10.1016/j.gaceta.2009.10.005

Vanneste, C., Barlow, P., & Rozenberg, S. (2020). Urgent medical aid and associated obstetric mortality in Belgium. *Journal of Immigrant and Minority Health. 22*(2), 307-313. https://doi.org/10.1007/s10903-019-00897-3

Vázquez, M. L., Terraza-Núñez, R., Vargas, I., Rodríguez, D., & Lizana, T. (2011). Health policies for migrant populations in three European countries: England; Italy and Spain. *Health policy. 101*(1), 70-78. https://doi.org/10.1016/j.healthpol.2010.08.026

Voorend, K., & Alvarado, D. (2023). Barriers to healthcare access for immigrants in Costa Rica and Uruguay. *Journal of International Migration and Integration. 24*(2), 747-771. https://doi.org/10.1007/s12134-022-00972-z

Voorend, K. (2014). “Shifting in” state sovereignty: social policy and migration control in Costa Rica. *Transnational Social Review. 4*(2-3), 207-225. https://doi.org/10.1080/21931674.2014.952977

Voorend, K., Bedi, A. S., & Sura-Fonseca, R. (2021). Migrants and access to health care in Costa Rica. *World Development. 144*, 105481. https://doi.org/10.1016/j.worlddev.2021.105481

Webb, E., Offe, J., & van Ginneken, E. (2022). Universal Health Coverage in the EU: What do we know (and not know) about gaps in access. *Eurohealth. 28*(3), 13-17.

Wendland, A., Ehmsen, B. K., Lenskjold, V., Astrup, B. S., Mohr, M., Williams, C. J., & Cowan, S. A. (2016). Undocumented migrant women in Denmark have inadequate access to pregnancy screening and have a higher prevalence Hepatitis B virus infection compared to documented migrants in Denmark: a prevalence study. *BMC Public Health. 16*(1), 426. https://doi.org/10.1186/s12889-016-3096-8

Willen, S. S. (2019). Fighting for dignity: migrant lives at Israel's margins. *University of Pennsylvania Press.*

Willen, S. S. (2011). Do “Illegal” Im/migrants have a right to health? Engaging ethical theory as social practice at a Tel Aviv Open Clinic. *Medical anthropology quarterly. 25*(3), 303-330. https://doi.org/10.1111/j.1548-1387.2011.01163.x

Zarca, K., Bekkar, Z., Rapp, T., Durand-Zaleski, I., & Feral-Pierssens, A. L. (2025). Hospital admissions for undocumented immigrants: a comparative analysis of French healthcare coverage schemes. *European Journal of Public Health. 35*(4), 687-692. https://doi,org/10.1093/eurpub/ckaf113

Zimmer, M. (2024). The other health care system in Germany: care for people without health insurance. *BMC* *Health Services Research. 24*(1), 1649. https://doi.org/10.1186/s12913-024-12119-0
