# Supplementary material for: Between duty and constraint: a qualitative systematic review of healthcare providers' ethical challenges and moral stressors in caring for undocumented migrants
Source: Int J Qual Stud Health Well-being. 2026 Jul 9;21(1):2701615. doi: 10.1080/17482631.2026.2701615 (PMC13353462; doi:10.1080/17482631.2026.2701615)
Supplement: PRISMA checklist.docx [file ZQHW_A_2701615_SM5348.docx]

| **Section and Topic** | **Item #** | **Checklist item** | **Location where item is reported** |
| --- | --- | --- | --- |
| **TITLE** | | |  |
| Title | 1 | Identify the report as a systematic review. | p. 1  Fulfilled |
| **ABSTRACT** | | |  |
| Abstract | 2 | See the PRISMA 2020 for Abstracts checklist. | p. 1  Partially fulfilled |
| **INTRODUCTION** | | |  |
| Rationale | 3 | Describe the rationale for the review in the context of existing knowledge. | p. 2-3  Fulfilled |
| Objectives | 4 | Provide an explicit statement of the objective(s) or question(s) the review addresses. | p. 3  Fulfilled |
| **METHODS** | | |  |
| Eligibility criteria | 5 | Specify the inclusion and exclusion criteria for the review and how studies were grouped for the syntheses. | p. 4, Table 2 & Figure 2  Fulfilled |
| Information sources | 6 | Specify all databases, registers, websites, organisations, reference lists and other sources searched or consulted to identify studies. Specify the date when each source was last searched or consulted. | p. 3  Fulfilled |
| Search strategy | 7 | Present the full search strategies for all databases, registers and websites, including any filters and limits used. | p. 3, Table 1  Partially fulfilled |
| Selection process | 8 | Specify the methods used to decide whether a study met the inclusion criteria of the review, including how many reviewers screened each record and each report retrieved, whether they worked independently, and if applicable, details of automation tools used in the process. | p. 4 & Figure 2  Fulfilled |
| Data collection process | 9 | Specify the methods used to collect data from reports, including how many reviewers collected data from each report, whether they worked independently, any processes for obtaining or confirming data from study investigators, and if applicable, details of automation tools used in the process. | p. 4-5, Figure 1 & Table 4  Partially fulfilled |
| Data items | 10a | List and define all outcomes for which data were sought. Specify whether all results that were compatible with each outcome domain in each study were sought (e.g. for all measures, time points, analyses), and if not, the methods used to decide which results to collect. | several locations, including the introduction, results, and conceptual scheme, as well as in Figure 1.  Fulfilled |
|  | 10b | List and define all other variables for which data were sought (e.g. participant and intervention characteristics, funding sources). Describe any assumptions made about any missing or unclear information. | p. 5 & Table 4  Mostly fulfilled |
| Study risk of bias assessment | 11 | Specify the methods used to assess risk of bias in the included studies, including details of the tool(s) used, how many reviewers assessed each study and whether they worked independently, and if applicable, details of automation tools used in the process. | p. 4 & Table 3  Mostly fulfilled |
| Effect measures | 12 | Specify for each outcome the effect measure(s) (e.g. risk ratio, mean difference) used in the synthesis or presentation of results. | Not applicable |
| Synthesis methods | 13a | Describe the processes used to decide which studies were eligible for each synthesis (e.g. tabulating the study intervention characteristics and comparing against the planned groups for each synthesis (item #5)). | Figures 1 & 3  Fulfilled, MAXQDA used for this purpose |
|  | 13b | Describe any methods required to prepare the data for presentation or synthesis, such as handling of missing summary statistics, or data conversions. | p. 4-5  Fully addressed in a qualitative context |
|  | 13c | Describe any methods used to tabulate or visually display results of individual studies and syntheses. | Both tabular & visual displays were used  Table 4, & Figures 1 & 3  Fulfilled |
|  | 13d | Describe any methods used to synthesize results and provide a rationale for the choice(s). If meta-analysis was performed, describe the model(s), method(s) to identify the presence and extent of statistical heterogeneity, and software package(s) used. | Not applicable  Fully addressed in a qualitative context  Figure 1 |
|  | 13e | Describe any methods used to explore possible causes of heterogeneity among study results (e.g. subgroup analysis, meta-regression). | Not applicable  Fully addressed in a qualitative context |
|  | 13f | Describe any sensitivity analyses conducted to assess robustness of the synthesized results. | Not applicable  Not performed |
| Reporting bias assessment | 14 | Describe any methods used to assess risk of bias due to missing results in a synthesis (arising from reporting biases). | Not applicable  Not performed |
| Certainty assessment | 15 | Describe any methods used to assess certainty (or confidence) in the body of evidence for an outcome. | Not formally fulfilled |
| **RESULTS** | | |  |
| Study selection | 16a | Describe the results of the search and selection process, from the number of records identified in the search to the number of studies included in the review, ideally using a flow diagram. | p. 5, Figure 2  Fulfilled |
|  | 16b | Cite studies that might appear to meet the inclusion criteria, but which were excluded, and explain why they were excluded. | Figure 2  Partially fulfilled |
| Study characteristics | 17 | Cite each included study and present its characteristics. | Results Section, Table 4, References  Mostly fulfilled |
| Risk of bias in studies | 18 | Present assessments of risk of bias for each included study. | Table 3  Fully addressed in a qualitative context |
| Results of individual studies | 19 | For all outcomes, present, for each study: (a) summary statistics for each group (where appropriate) and (b) an effect estimate and its precision (e.g. confidence/credible interval), ideally using structured tables or plots. | Not applicable  Fully addressed in a qualitative context |
| Results of syntheses | 20a | For each synthesis, briefly summarise the characteristics and risk of bias among contributing studies. | Characteristics (fully addressed in a qualitative context)  Risk of bias (not applicable) |
|  | 20b | Present results of all statistical syntheses conducted. If meta-analysis was done, present for each the summary estimate and its precision (e.g. confidence/credible interval) and measures of statistical heterogeneity. If comparing groups, describe the direction of the effect. | Not applicable |
|  | 20c | Present results of all investigations of possible causes of heterogeneity among study results. | Results & discussion sections  Fully addressed in a qualitative context |
|  | 20d | Present results of all sensitivity analyses conducted to assess the robustness of the synthesized results. | Not applicable |
| Reporting biases | 21 | Present assessments of risk of bias due to missing results (arising from reporting biases) for each synthesis assessed. | Not applicable  Fully addressed in a qualitative context |
| Certainty of evidence | 22 | Present assessments of certainty (or confidence) in the body of evidence for each outcome assessed. | Partially fulfilled  No formal tool was applied |
| **DISCUSSION** | | |  |
| Discussion | 23a | Provide a general interpretation of the results in the context of other evidence. | p. 16-22  Discussion section  Fulfilled |
|  | 23b | Discuss any limitations of the evidence included in the review. | No major limitations of the evidence were identified |
|  | 23c | Discuss any limitations of the review processes used. | p. 23  Fulfilled |
|  | 23d | Discuss implications of the results for practice, policy, and future research. | p. 22-23, Table 5  Fulfilled |
| **OTHER INFORMATION** | | |  |
| Registration and protocol | 24a | Provide registration information for the review, including register name and registration number, or state that the review was not registered. | The review was not registered |
|  | 24b | Indicate where the review protocol can be accessed, or state that a protocol was not prepared. | A protocol was not prepared |
|  | 24c | Describe and explain any amendments to information provided at registration or in the protocol. | Not applicable |
| Support | 25 | Describe sources of financial or non-financial support for the review, and the role of the funders or sponsors in the review. | p. 24  A funding statement is included in the manuscript |
| Competing interests | 26 | Declare any competing interests of review authors. | p. 24  Competing interests, if any, are declared in the manuscript |
| Availability of data, code and other materials | 27 | Report which of the following are publicly available and where they can be found: template data collection forms; data extracted from included studies; data used for all analyses; analytic code; any other materials used in the review. | p. 24  Data will be made available on reasonable request |

*From:*  Page MJ, McKenzie JE, Bossuyt PM, Boutron I, Hoffmann TC, Mulrow CD, et al. The PRISMA 2020 statement: an updated guideline for reporting systematic reviews. BMJ 2021;372:n71. doi: 10.1136/bmj.n71. This work is licensed under CC BY 4.0. To view a copy of this license, visit <https://creativecommons.org/licenses/by/4.0/>

**Notes on items:**

**Item 1:** Identify the report as a systematic review

The title includes the phrase “systematic review”, which fulfills the requirement to explicitly identify the report as a systematic review. Additionally, it provides key information about:

- The main topic: ethical challenges and moral stressors,
- The population of interest: healthcare providers,
- The focus: caring for undocumented migrants,
- The methodological scope: qualitative evidence synthesis.

**Item 2:** See the PRISMA 2020 for Abstracts checklist

The abstract addresses the majority of the items listed in the ”PRISMA 2020 for Abstracts checklist”. Specifically, items 1, 2, 5, 6, 7, 8, and 10 are fully fulfilled, item 4 is partially fulfilled, while items 3, 9, 11, and 12 are not fully addressed. A detailed table summarizing the fulfillment status of each item is provided below.

While efforts were made to cover as many checklist elements as possible, practical limitations related to word count constraints necessitated prioritization. Given the need for clarity, conciseness, and balance across the different sections of the abstract, we focused on ensuring that the purpose, key methodological approach, principal findings, and implications were clearly presented. Expanding the methods section to include detailed eligibility criteria, limitations, and other details would have significantly reduced the space available for other essential components.

It is important to note that although some checklist items are not reported within the abstract due to these constraints, they are comprehensively addressed in the full manuscript.

**Assessment of the 12 PRISMA Abstract Items:**

| **Item** | **Requirement** | **Fulfillment** | **Justification** |
| --- | --- | --- | --- |
| **1** | Identify the report as a systematic review | Yes | “Systematic review” is stated in the title and first sentence of abstract. |
| **2** | Provide an explicit statement of the main objective(s) or question(s) the review addresses | Yes | Clearly stated in the purpose. |
| **3** | Specify the inclusion and exclusion criteria for the review | No | The abstract does not mention inclusion/exclusion criteria. |
| **4** | Specify the information sources (such as databases, registers) used to identify studies and the date when each was last searched | Partially | Databases are mentioned but dates are not included. |
| **5** | Specify the methods used to assess risk of bias in the included studies | Yes | "Quality assessment was conducted using the CASP checklist" is stated. |
| **6** | Specify the methods used to present and synthesize results | Yes | QUAGOL and content analysis methods mentioned. |
| **7** | Give the total number of included studies and participants and summarise relevant characteristics of studies | Yes | "37 qualitative studies" mentioned, with general description. |
| **8** | Present results for main outcomes | Yes | Key findings and themes summarized. |
| **9** | Provide a brief summary of limitations of the evidence | No | No mention of limitations in the abstract. |
| **10** | Provide a general interpretation and implications | Yes | Clear implications about ethical landscape and policy needs are included. |
| **11** | Specify the primary source of funding | No | No funding information is provided in the abstract. |
| **12** | Provide the register name and registration number | No | No registration info is mentioned. |

**Item 3:** Describe the rationale for the review in the context of existing knowledge

The rationale for conducting the review is fully described in the Introduction section. We provide a detailed overview of the existing body of knowledge regarding the healthcare barriers faced by undocumented migrants, while highlighting the lack of comprehensive understanding of the ethical challenges and moral stressors experienced by healthcare providers. We explicitly articulate the uncertainties in the current literature and the absence of systematic reviews synthesizing qualitative evidence on this topic. Additionally, we justify the need for the review by emphasizing the importance of consolidating existing qualitative findings to inform policy reforms, raise awareness, and support provider well-being. Thus, the rationale is clearly contextualized within the framework of existing knowledge and knowledge gaps.

**Item 4:** Provide an explicit statement of the objective(s) or question(s) the review addresses

An explicit and concise statement of the review objective is provided in the Introduction section. The review is guided by the following central research question: "What ethical challenges and moral stressors arise for healthcare providers when delivering care to undocumented migrants?" This formulation clearly conveys the focus and scope of the review in a way that is appropriate for a qualitative evidence synthesis.

While PRISMA recommends expressing objectives using a formal question formulation framework, we note that qualitative systematic reviews often require greater flexibility compared to quantitative reviews. In qualitative syntheses, the emphasis is typically on exploring experiences, perceptions, or processes rather than measuring outcomes or comparing interventions. Accordingly, although we organized our search strategy around major conceptual blocks (undocumented migrants, healthcare providers, and ethical challenges), we did not rigidly apply a question formulation framework. Instead, we adapted the formulation to suit the nature of qualitative inquiry, focusing on the exploration of complex ethical phenomena rather than on interventional effects.

**Item 5:** Specify the inclusion and exclusion criteria for the review and how studies were grouped for the syntheses

The inclusion and exclusion criteria for the review are fully specified in Table 2. The process of study selection and exclusion is further illustrated in the PRISMA flow diagram (Figure 2). Regarding grouping for synthesis, studies were not categorized based on intervention or outcome groups, as is typical in quantitative reviews, but were thematically organized according to major concepts emerging from the qualitative analysis (e.g., experiences, perceptions, attitudes, practices and coping mechanisms, and ethical challenges). This approach is consistent with accepted methods for synthesizing qualitative evidence and reflects necessary adaptations when applying PRISMA standards to qualitative systematic reviews.

**Item 6:** Specify all databases, registers, websites, organisations, reference lists and other sources searched or consulted to identify studies. Specify the date when each source was last searched or consulted

The information sources used to identify studies are detailed in the Methods section and further illustrated in the PRISMA flow diagram (Figure 2). We acknowledge that, while the database names and the date of the last search are reported, one element was not specified: the dates of coverage for each database (e.g., 1946 to present for Medline). This level of detail was not provided in the manuscript. Nevertheless, all major essential elements — including the names of the databases searched, the date of the last search, and the use of reference list searching — are reported, fulfilling the core intent of this PRISMA item.

**Item 7:** Present the full search strategies for all databases, registers and websites, including any filters and limits used

The full search strategy for the PubMed/Medline database is reported in Table 1, structured across three conceptual search blocks relevant to our research question. No formal date limits were applied within the search strategies, although language restriction (English) was incorporated through the eligibility criteria. We acknowledge that full search strategies for the other databases (Embase, CINAHL, and Cochrane Library) are not presented in the manuscript. However, all searches were conducted using comparable search blocks adapted to the controlled vocabularies and indexing terms of each database. No search filters were used or adapted, and no formal peer review (such as PRESS) of the search strategy was conducted. Although this item is only partially fulfilled, all major search concepts, sources, and strategies are transparently reported, enabling readers to assess the comprehensiveness of the search and facilitating potential updating of the review.

**Item 8:** Specify the methods used to decide whether a study met the inclusion criteria of the review, including how many reviewers screened each record and each report retrieved, whether they worked independently, and if applicable, details of automation tools used in the process

This item is addressed in the Methods section (Page 4), which specifies the reviewer roles and the resolution process for inclusion decisions. The PRISMA flow diagram (Figure 2) further details the number of records screened at each stage and provides explicit reasons for study exclusion. No automation tools, machine learning classifiers, or crowdsourcing platforms were used during the study selection process; all screening was conducted manually by the review team.

**Item 9:** Specify the methods used to collect data from reports, including how many reviewers collected data from each report, whether they worked independently, any processes for obtaining or confirming data from study investigators, and if applicable, details of automation tools used in the process

The data collection process is described in the Methods section, where we explain the use of the Qualitative Analysis Guide of Leuven (QUAGOL) framework. This approach includes ten structured stages, with the first five stages focused on data familiarization and preparation for coding (see Figure 1). These stages involved collaborative reading, summarizing, and organizing information from each included study. Although we used a structured data collection form to record study-level characteristics—summarized in Table 4—this tool is not explicitly mentioned in the manuscript. We did not specify the number of reviewers or their individual roles in the data extraction process, as we considered this level of detail less relevant within the collaborative and iterative structure of the QUAGOL approach. No automation tools or translation processes were used.

**Item 10a:** List and define all outcomes for which data were sought. Specify whether all results that were compatible with each outcome domain in each study were sought (e.g. for all measures, time points, analyses), and if not, the methods used to decide which results to collect

This item is fully addressed. As a qualitative evidence synthesis, our review did not predefine outcomes as in quantitative reviews but instead focused on conceptually rich domains relevant to our research question—specifically, ethical challenges and moral stressors experienced by healthcare providers when caring for undocumented migrants. These domains were conceptually defined in the Introduction and guided the analytic process. Using the QUAGOL framework, themes were inductively generated, refined, and organized into a conceptual structure, as detailed in Figure 1. The general meaning of each concept was clarified at the beginning of each thematic section in the Results. We sought to include all findings from each study relevant to the domains of interest, and cross-comparative analysis ensured consistency and depth across the synthesized data.

**Item 10b:** List and define all other variables for which data were sought (e.g. participant and intervention characteristics, funding sources). Describe any assumptions made about any missing or unclear information

This item is mostly fulfilled. As summarized in Table 4, we extracted several study- and participant-level variables. These variables were not presented individually for each study but were aggregated across all included studies. Funding sources and competing interests were not collected, as they were not considered directly relevant to the review objectives. We did not formally document assumptions for missing or unclear information because the majority of studies provided sufficient detail, and no substantial gaps or ambiguities were encountered during data extraction.

**Item 11:** Specify the methods used to assess risk of bias in the included studies, including details of the tool(s) used, how many reviewers assessed each study and whether they worked independently, and if applicable, details of automation tools used in the process

This item is mostly fulfilled. In qualitative evidence syntheses, the assessment of risk of bias is typically framed as a process of evaluating “methodological limitations” or “critical appraisal”, rather than applying the specific concept of “risk of bias” used in quantitative intervention reviews, which focuses on how methodological flaws may distort statistical and numerical findings. We used the Critical Appraisal Skills Programme (CASP) checklist for qualitative research, a widely accepted tool for assessing methodological rigor in qualitative studies. CASP evaluates not only features that may introduce bias, but also elements related to ethical integrity, contextual relevance, reflexivity, and analytic transparency. While PRISMA distinguishes between risk of bias and broader quality constructs, tools like CASP are considered appropriate for qualitative reviews and fulfill this item when clearly reported. As described in the Methods section (“Quality Appraisal”) and summarized in Table 3, we systematically assessed each study using the ten CASP criteria. No studies were excluded based on their quality ratings. No automation tools were used in the quality assessment process.

**Item 12:** Specify for each outcome the effect measure(s) (e.g. risk ratio, mean difference) used in the synthesis or presentation of results

This item is not applicable. In quantitative systematic reviews, this item refers to the statistical constructs used to measure the size or direction of an effect (e.g., risk ratio, mean difference, odds ratio). In contrast, qualitative evidence syntheses do not use effect measures, as they do not quantify change or comparison. Instead, they synthesize patterns, meanings, and conceptual insights derived from textual data. The emphasis is on the depth, richness, and credibility of interpretation rather than statistical magnitude or thresholds. In our review, we applied a thematic and conceptual synthesis approach to explore the ethical challenges and moral stressors experienced by healthcare providers, with no use of statistical effect measures or quantitative comparisons.

**Item 13a:** Describe the processes used to decide which studies were eligible for each synthesis (e.g. tabulating the study intervention characteristics and comparing against the planned groups for each synthesis (item #5))

The concept of “eligibility for synthesis” relates to the conceptual relevance of each included study to the emerging thematic framework. In our review, all studies that met the inclusion criteria (as outlined under Item 5) contributed to the synthesis. Using the QUAGOL framework, we systematically familiarized ourselves with each study, developed narrative summaries, and coded data using MAXQDA software. The coding process grouped relevant text segments by theme across all studies, allowing us to identify which studies contributed to each conceptual domain. These domains — experiences, perceptions, attitudes, practices & coping, and ethical challenges — are visually presented in Figure 3. The process of grouping and synthesizing studies into these domains is further outlined in Figure 1, which illustrates the staged transition from raw data to integrated thematic frameworks. The use of MAXQDA provided structured support for identifying the thematic contributions of each study across the synthesis.

**Item 13b:** Describe any methods required to prepare the data for presentation or synthesis, such as handling of missing summary statistics, or data conversions

This item is fully addressed in the context of a qualitative evidence synthesis. Our review did not involve statistical data conversions or the imputation of missing numerical values, as typically required in quantitative syntheses. Instead, data preparation involved the interpretive organization and structuring of textual data, guided by the QUAGOL framework. This process included re-reading and summarizing the included studies, developing conceptual schemes, and coding data using MAXQDA software. The software supported the systematic categorization and retrieval of coded segments across studies. These qualitative data preparation steps are described in the Methods section and visualized in Figure 1.

**Item 13c:** Describe any methods used to tabulate or visually display results of individual studies and syntheses

We used tabular and visual displays to support transparency and illustrate key characteristics and conceptual patterns across the included studies. Table 4 presents an aggregated summary of study characteristics—including country, data collection method, sector, field, and provider role—rather than individual study findings. Figure 1 visualizes the staged synthesis process based on the QUAGOL framework, demonstrating how data were systematically organized and coded. Figure 3 presents the final conceptual structure, showing the five overarching domains developed inductively through synthesis of the collective findings. These formats were selected to help readers understand the analytic process and thematic outcomes at the synthesis level, rather than on a per-study basis. No statistical plots or study-level effect estimates were applicable.

**Item 13d:** Describe any methods used to synthesize results and provide a rationale for the choice(s). If meta-analysis was performed, describe the model(s), method(s) to identify the presence and extent of statistical heterogeneity, and software package(s) used

This item is fully addressed within the context of a qualitative evidence synthesis. We did not perform a statistical meta-analysis, and effect size modeling, heterogeneity assessment, or quantitative synthesis methods were not applicable. Instead, we employed the QUAGOL framework to guide the thematic synthesis (see Figure 1). MAXQDA software was used to facilitate data management, coding organization, and synthesis across studies. We also incorporated elements of Graneheim and Lundman’s approach to qualitative content analysis to refine coding and ensure depth in thematic interpretation. This combination was selected for its ability to support both case-oriented and cross-case analysis, reflexivity, and interpretive rigor—features especially important for examining ethical challenges in complex healthcare contexts.

**Item 13e:** Describe any methods used to explore possible causes of heterogeneity among study results (e.g. subgroup analysis, meta-regression)

In qualitative reviews, heterogeneity is explored conceptually rather than statistically. Our synthesis examined variation across studies in relation to contextual, professional, and thematic factors. Using the QUAGOL framework, we conducted a cross-case comparison that allowed us to explore how ethical challenges and moral stressors manifested differently across settings, provider roles, and health system contexts. No statistical methods such as subgroup analysis or meta-regression were used, as they are not applicable to qualitative data synthesis.

**Item 13f:** Describe any sensitivity analyses conducted to assess robustness of the synthesized results

No formal sensitivity analyses were conducted. In qualitative evidence synthesis, sensitivity analysis may involve examining the influence of lower-quality studies or reassessing themes through alternative coding strategies. While we did not perform a post-synthesis sensitivity analysis, the robustness of our findings was supported through iterative team coding, re-examination of conceptual schemes, and the use of the QUAGOL framework’s multi-stage synthesis process. Additionally, we conducted a systematic quality appraisal of included studies (Table 3), though no studies were excluded based on quality, and all were retained in the synthesis.

**Item 14:** Describe any methods used to assess risk of bias due to missing results in a synthesis (arising from reporting biases)

Unlike quantitative reviews, qualitative syntheses do not rely on pre-registered outcomes, and we are not aware of any validated tools currently available to assess selective non-reporting in qualitative data. However, as acknowledged in the limitations section, our synthesis was limited to peer-reviewed studies published in English, and we did not include grey literature. These restrictions may have contributed to the exclusion of relevant studies and perspectives, particularly from underrepresented regions or groups. While no formal analysis of publication or reporting bias was performed, all studies meeting the inclusion criteria—regardless of their findings—were considered equally in the synthesis.

**Item 15:** Describe any methods used to assess certainty (or confidence) in the body of evidence for an outcome

We did not apply a formal tool such as GRADE-CERQual to assess confidence in each review finding. Frameworks such as GRADE-CERQual offer structured approaches to evaluate confidence in the body of evidence based on methodological limitations, coherence, adequacy, and relevance. While our review did not use this framework, we ensured analytical rigor and transparency through several strategies: methodological quality appraisal using the CASP checklist (Table 3), a structured multi-stage thematic synthesis using the QUAGOL framework, and collaborative coding. Conceptual coherence was strengthened through constant comparison, and thematic saturation was achieved across the included studies, supporting the completeness of the synthesis. Nevertheless, we acknowledge that no explicit confidence ratings were assigned to individual review findings.

**Item 16a:** Describe the results of the search and selection process, from the number of records identified in the search to the number of studies included in the review, ideally using a flow diagram

This item is fully addressed. The results of the search and selection process are reported in the manuscript and summarized in Figure 2 (PRISMA flow diagram). The flow diagram details each stage of the selection process, including reasons for exclusion, in accordance with PRISMA guidance.

**Item 16b:** Cite studies that might appear to meet the inclusion criteria, but which were excluded, and explain why they were excluded

This item is partially fulfilled. As shown in Figure 2, we provide a summary of the number of full-text articles excluded, categorized by reason (e.g., non-qualitative design, wrong population, scope mismatch). However, we did not include a separate table listing citations and individual justifications for studies that may appear to meet the inclusion criteria. While reasons for exclusion were documented during the screening process, they were not tabulated in the final report. It is also important to note that all studies that met our inclusion criteria were included in the synthesis; no studies were excluded after meeting the eligibility criteria.

**Item 17:** Cite each included study and present its characteristics

This item is mostly fulfilled. All 37 included studies are cited in the manuscript and listed in the reference section. Key characteristics of the included studies are collectively summarized in Table 4 but not presented for each study individually. For the purpose of our review, this approach was considered satisfactory, as our primary objective was to present and explore the concepts, themes, and subthemes that emerged through synthesis. All included studies are cited in association with the relevant themes or concepts they contributed to, thereby ensuring transparency regarding the analytical basis of our findings.

**Item 18:** Present assessments of risk of bias for each included study

This item is fully addressed using an approach appropriate for qualitative evidence synthesis. We assessed the methodological limitations of each included study using the CASP checklist for qualitative research. The results of this appraisal are presented in Table 3, where we provide individual scores for each CASP criterion and an overall quality score for each study. While we did not present verbatim justifications for each judgment, the structured scoring system transparently reflects the methodological rigor of each study and supports the interpretive validity of the synthesis.

In quantitative reviews, the concept of risk of bias typically refers to specific design-related flaws—such as randomization, blinding, or selective outcome reporting—that may systematically distort effect estimates. In contrast, qualitative reviews assess methodological limitations more broadly, encompassing issues like researcher reflexivity, ethical conduct, analytical rigor, and the clarity and credibility of reported findings.

Although PRISMA distinguishes between “risk of bias” and “quality assessment”, in qualitative research these domains are often interdependent and assessed together. The CASP checklist, while technically framed as a quality appraisal tool, includes several items directly related to risk of bias in qualitative findings. In our review, we considered the following CASP questions as most relevant to assessing risk of bias in results:

- Q5: Was the data collected in a way that addressed the research issue?
- Q6: Has the relationship between researcher and participants been adequately considered?
- Q8: Was the data analysis sufficiently rigorous?
- Q9: Is there a clear statement of findings?

These items speak to issues of trustworthiness, credibility, and analytic transparency — all of which are central to evaluating the risk of biased or unsubstantiated interpretations in qualitative evidence.

**Item 19:** For all outcomes, present, for each study: (a) summary statistics for each group (where appropriate) and (b) an effect estimate and its precision (e.g. confidence/credible interval), ideally using structured tables or plots

Our review did not involve any statistical comparisons, group-level summary statistics, or effect estimates. Instead, we presented results thematically, drawing on textual data from each included study. Thematic domains and subthemes are described in the Results section, with each theme supported by direct citations of the studies that contributed relevant data. This approach ensured transparency and traceability of study contributions in line with qualitative review standards, even though no structured tables or statistical plots were used.

**Item 20a:** For each synthesis, briefly summarise the characteristics and risk of bias among contributing studies

Our synthesis was organized thematically, and contributing studies are cited under each of the five major conceptual domains in the Results section. While we did not include an explicit summary table of study characteristics or risk of bias per theme, this approach is not typically applicable in qualitative evidence synthesis. Unlike quantitative reviews, where each synthesis may relate to a distinct outcome and allow for domain-specific bias assessment, qualitative themes often emerge inductively from overlapping sets of studies. A single study may contribute to multiple themes, and thematic findings do not represent fixed outcomes to which effect estimates, or risk of bias adjustments can be applied.

Instead, we ensured methodological robustness and quality by appraising all included studies using the CASP checklist, as presented in Table 3. This allowed us to verify the credibility of our evidence base. Where relevant, our narrative synthesis reflects contextual variations that may shape theme development. We also ensured that themes were grounded in multiple methodologically sound studies, and thematic saturation was achieved, supporting the conceptual strength and transferability of the findings.

**Item 20b:** Present results of all statistical syntheses conducted. If meta-analysis was done, present for each the summary estimate and its precision (e.g. confidence/credible interval) and measures of statistical heterogeneity. If comparing groups, describe the direction of the effect

This item is not applicable. No statistical synthesis or meta-analysis was conducted, as this review synthesized qualitative evidence. Instead of calculating effect estimates or summary statistics, we conducted a thematic synthesis to identify key conceptual domains and subthemes emerging from the included studies. The synthesis was narrative and interpretive in nature, grounded in the QUAGOL framework and supported by iterative analysis and thematic saturation.

**Item 20c:** Present results of all investigations of possible causes of heterogeneity among study results

This item is fulfilled using an approach appropriate for qualitative evidence synthesis. While we did not conduct any statistical subgroup analyses or meta-regressions, we explored conceptual and contextual variation across studies throughout the synthesis. Our thematic findings reflect variations in setting (e.g., governmental vs. humanitarian sector), healthcare provider roles, and broader institutional, legal, and health system contexts. These differences were considered during analysis and interpretation, allowing us to capture how similar ethical challenges may manifest differently depending on contextual factors.

**Item 20d:** Present results of all sensitivity analyses conducted to assess the robustness of the synthesized results

This item is not applicable. Sensitivity analyses in quantitative reviews typically involve re-running statistical models to assess whether results are robust to specific decisions, such as excluding studies at high risk of bias or using alternative effect measures. In qualitative evidence synthesis, by contrast, robustness is assessed through methodological rigor, interpretive depth, and analytical transparency rather than statistical testing.

We did not conduct formal sensitivity analyses, such as removing studies to test the stability of themes. However, we considered the frequency with which themes appeared across studies as one input, but this was not the decisive factor in determining thematic relevance. More important to us was the richness, depth, and conceptual weight of a theme. For example, a theme emerging from only two studies was still included if those studies presented strong, detailed accounts that captured critical ethical experiences or unique insights.

Robustness was further supported through the use of a structured, collaborative synthesis process (QUAGOL), inclusion of methodologically sound studies based on CASP appraisal, and thematic saturation across the data. These strategies collectively ensured the reliability of our findings in a manner consistent with qualitative review standards.

**Item 21:** Present assessments of risk of bias due to missing results (arising from reporting biases) for each synthesis assessed

We did not apply any formal tools or statistical tests to assess risk of bias due to missing results, as these are not typically used in qualitative evidence synthesis. However, we acknowledge that our search was limited to English-language, peer-reviewed articles, and we did not include grey literature. These restrictions may have introduced a risk of missing relevant perspectives, particularly from underrepresented settings or voices.

To minimize potential reporting bias, we applied broad eligibility criteria and included all studies that met the inclusion criteria, regardless of setting, health sector, or field. We emphasized the conceptual richness of themes rather than their frequency. Even themes expressed in only two studies were retained when they reflected analytically rich or ethically significant experiences. This approach helped ensure that a diversity of perspectives was captured and transparently synthesized.

**Item 22:** Present assessments of certainty (or confidence) in the body of evidence for each outcome assessed

This item is addressed using principles aligned with qualitative evidence synthesis, though no formal GRADE-CERQual assessment was conducted. The GRADE-CERQual approach recommends evaluating confidence in each review finding based on four components: (1) methodological limitations, (2) coherence, (3) adequacy of data, and (4) relevance. While we did not explicitly apply the GRADE-CERQual framework, our analytical procedures reflect attention to each of these domains:

- **Methodological limitations** were systematically assessed for all included studies using the CASP checklist (Table 3), allowing us to identify potential weaknesses in design or reporting.
- **Coherence** was addressed through constant comparative analysis and inductive theme refinement within the QUAGOL framework, ensuring a strong alignment between data excerpts and synthesized findings.
- **Adequacy of data** was considered through both the volume and richness of evidence contributing to each theme. Importantly, thematic saturation was used as a guiding principle—ensuring that no new concepts were emerging and that findings were well-supported across studies.
- **Relevance** was considered by including studies from diverse healthcare settings and contexts, and synthesizing data relevant to our research question.

Although we did not assign formal confidence ratings to each theme, we strived for analytical transparency and conceptual robustness. All themes were grounded in multiple methodologically sound studies, but we also retained themes that were drawn from fewer studies when they reflected unique provider experiences. For example, a theme based on only two studies was still included when it represented a distinctive moral or professional tension that was analytically rich and contextually significant.

**Item 23a:** Provide a general interpretation of the results in the context of other evidence

This item is fulfilled. In the Discussion section, we provide a general interpretation of our findings in the context of existing literature. Specifically, we compare the results of our thematic synthesis to prior conceptual, empirical, and normative studies that have examined healthcare providers' experiences and ethical challenges when caring for undocumented migrants. We identify points of agreement and divergence, such as how our findings expand upon previous reviews by highlighting not only systemic barriers but also the relational and moral tensions that healthcare providers face in practice.

In particular, we note that while some previous reviews focus primarily on structural determinants or access to services, our review adds depth by exploring the lived ethical experiences of providers, including their professional dilemmas. We also reflect on how our synthesis builds on and goes beyond existing ethical frameworks, and where appropriate, we speculate on reasons for differences between our findings and those reported in prior research. This integrative approach allows us to situate our results within the broader field of research and offers readers a clear sense of how our synthesis contributes novel insights to the evidence base.

**Item 23b:** Discuss any limitations of the evidence included in the review

We did not identify any major limitations in the body of evidence included in the review. The methodological quality of the included studies was generally high, as assessed using the CASP checklist (see Table 3), and most studies demonstrated strong attention to ethical rigor, clarity of reporting, and analytical depth.

While some of the included studies were based on small samples, we did not consider this a limitation in the context of qualitative research, where the richness and relevance of the data are more important than sample size alone. Thematic adequacy and conceptual saturation were achieved across studies, and even findings emerging from a limited number of studies were retained when they offered distinctive or analytically rich insights. Therefore, the evidence base was deemed sufficiently robust to support a comprehensive and conceptually grounded synthesis.

**Item 23c:** Discuss any limitations of the review processes used

We discuss several limitations related to the review process in the Limitations subsection of the Discussion. These limitations were transparently acknowledged and considered in interpreting the findings.

**Item 23d:** Discuss implications of the results for practice, policy, and future research

In the Discussion section and in Table 5, we explicitly present implications of the review findings for healthcare providers, institutions, policymakers, and researchers. Our recommendations are actionable and aligned with the ethical complexities highlighted by the synthesis, offering a comprehensive roadmap for systemic and practical reform in the delivery of healthcare to undocumented migrants.
