# Supplementary figures and images for: Between duty and constraint: a qualitative systematic review of healthcare providers' ethical challenges and moral stressors in caring for undocumented migrants
Source: Int J Qual Stud Health Well-being. 2026 Jul 9;21(1):2701615. doi: 10.1080/17482631.2026.2701615 (PMC13353462; doi:10.1080/17482631.2026.2701615)

**Conceptual Model**


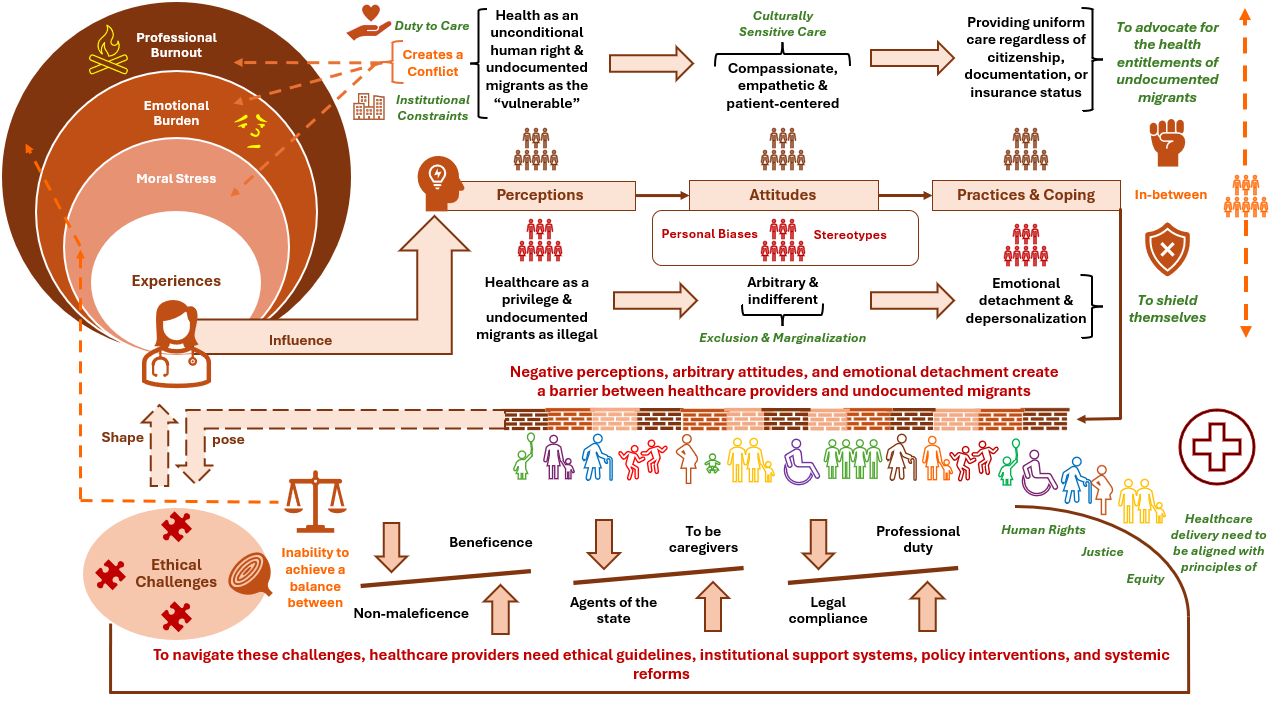

Supplement: Conceptual Model.docx [file ZQHW_A_2701615_SM5349.docx]
