# Supplementary material for: Between duty and constraint: a qualitative systematic review of healthcare providers' ethical challenges and moral stressors in caring for undocumented migrants
Source: Int J Qual Stud Health Well-being. 2026 Jul 9;21(1):2701615. doi: 10.1080/17482631.2026.2701615 (PMC13353462; doi:10.1080/17482631.2026.2701615)
Supplement: Quality assessment of included studies.docx [file ZQHW_A_2701615_SM5350.docx]

Quality assessment of included studies using the CASP checklist for qualitative studies.

| No. | Study | 1. Was there a clear statement of the aims of the research? | 2. Is a qualitative methodology appropriate? | 3. Was the research design appropriate to address the aims of the research? | 4. Was the recruitment strategy appropriate to the aims of the research | 5. Was the data collected in a way that addressed the research issue? | 6. Has the relationship between researcher and participants been adequately considered? | 7. Have ethical issues been taken into consideration? | 8. Was the data analysis sufficiently rigorous? | 9. Is there a clear statement of findings? | 10. How valuable is the research? | Overall assessment |
| --- | --- | --- | --- | --- | --- | --- | --- | --- | --- | --- | --- | --- |
| 1 | Goldade. 2009 | Y | Y | Y | U | Y | Y | N | U | Y | Y | 16 G |
| 2 | Biswas et al. 2011 | Y | Y | Y | U | Y | Y | Y | U | Y | Y | 18 H |
| 3 | Castaneda. 2011 | Y | Y | Y | U | Y | N | N | U | Y | Y | 14 G |
| 4 | Jensen et al. 2011 | Y | Y | Y | Y | U | Y | N | U | Y | Y | 16 G |
| 5 | Willen. 2011 | Y | Y | Y | Y | Y | Y | Y | Y | Y | Y | 20 H |
| 6 | Dauvrin et al. 2012 | Y | Y | Y | Y | Y | U | Y | Y | Y | Y | 19 H |
| 7 | Holmes. 2012 | Y | Y | Y | Y | Y | Y | U | U | Y | Y | 18 H |
| 8 | Marrow. 2012 | Y | Y | U | Y | U | N | Y | N | Y | Y | 14 G |
| 9 | Straβmayr et al. 2012 | Y | Y | Y | Y | Y | U | Y | Y | Y | Y | 19 H |
| 10 | Gullberg et al. 2014 | Y | Y | Y | Y | Y | U | Y | Y | U | U | 17 H |
| 11 | Tiedje et al. 2014 | Y | Y | Y | Y | Y | U | Y | Y | Y | Y | 19 H |
| 12 | Dos Santos. 2015 | Y | Y | U | U | U | N | U | N | Y | Y | 12 M |
| 13 | Teunissen et al. 2015 | Y | Y | Y | Y | Y | U | Y | Y | Y | Y | 19 H |
| 14 | Sandblom et al. 2017 | Y | Y | Y | Y | Y | Y | Y | Y | Y | Y | 20 H |
| 15 | Cervantes et al. 2018 | Y | Y | Y | Y | Y | N | Y | Y | Y | Y | 18 H |
| 16 | Armin. 2019 | Y | Y | Y | Y | U | U | N | N | U | Y | 13 G |
| 17 | Bianchi et al. 2019 | Y | Y | Y | Y | Y | Y | Y | Y | Y | Y | 20 H |
| 18 | Fabi et al. 2019 | Y | Y | Y | Y | Y | N | U | Y | Y | Y | 17 H |
| 19 | Lopez-Domene et al. 2019 | Y | Y | Y | Y | Y | Y | Y | Y | Y | Y | 20 H |
| 20 | Doshi et al. 2020 | Y | Y | Y | Y | Y | Y | Y | Y | Y | Y | 20 H |
| 21 | Sahraoui. 2020 | Y | Y | Y | Y | U | N | Y | N | Y | Y | 15 G |
| 22 | Yu et al. 2020 | Y | Y | Y | Y | Y | Y | Y | Y | Y | Y | 20 H |
| 23 | Granero-Molina et al. 2021 | Y | Y | Y | Y | Y | Y | Y | Y | Y | Y | 20 H |
| 24 | Hoekstra. 2021 | Y | Y | Y | Y | Y | Y | Y | U | Y | Y | 19 H |
| 25 | Lafaut. 2021 | Y | Y | Y | Y | Y | N | Y | Y | Y | Y | 18 H |
| 26 | Midde et al. 2021 | Y | Y | Y | Y | Y | Y | Y | Y | Y | Y | 20 H |
| 27 | Saadi et al. 2021 | Y | Y | U | Y | Y | Y | Y | U | Y | Y | 18 H |
| 28 | Tschirhart et al. 2021 | Y | Y | U | Y | Y | Y | Y | U | Y | Y | 18 H |
| 29 | Granero-Molina et al. 2022 | Y | Y | Y | Y | Y | Y | Y | Y | Y | Y | 20 H |
| 30 | Kvamme et al. 2022 | Y | Y | Y | Y | Y | U | Y | Y | Y | Y | 19 H |
| 31 | Vanobberghen et al. 2022 | Y | Y | Y | Y | Y | Y | Y | N | Y | Y | 18 H |
| 32 | Mladovsky. 2023 | Y | Y | Y | Y | Y | N | Y | N | Y | Y | 16 G |
| 33 | Jiménez-Lasserrotte et al. 2023 | Y | Y | Y | Y | Y | Y | Y | Y | Y | Y | 20 H |
| 34 | Voldner et al. 2023 | Y | Y | Y | Y | U | N | Y | U | Y | Y | 16 G |
| 35 | Worthing et al. 2023 | Y | Y | Y | Y | Y | U | U | U | Y | Y | 17 H |
| 36 | Gely et al. 2023 | Y | Y | U | Y | Y | Y | Y | Y | Y | Y | 19 H |
| 37 | Piccoli et al. 2024 | Y | Y | Y | Y | U | N | N | N | U | Y | 12 M |
| Note: Y = Yes, N = No, U = Unclear, H = High, G = Good, and M = Moderate  Scoring: Yes = 2, Unclear = 1, and No = 0 | | | | | | | | | | | | |
